# Global changes and their environmental stressors have a significant impact on soil biodiversity—A meta-analysis

**DOI:** 10.1016/j.isci.2024.110540

**Published:** 2024-07-20

**Authors:** Helen R.P. Phillips, Erin K. Cameron, Nico Eisenhauer, Victoria J. Burton, Olga Ferlian, Yiming Jin, Sahana Kanabar, Sandhya Malladi, Rowan E. Murphy, Anne Peter, Isis Petrocelli, Christian Ristok, Katharine Tyndall, Wim van der Putten, Léa Beaumelle

**Affiliations:** 1 Department of Terrestrial Ecology, Netherlands Institute of Ecology (NIOO-KNAW), Wageningen, the Netherlands; 2 Department of Environmental Sciences, Saint Mary’s University, Halifax, NS, Canada; 3 Organismal and Evolutionary Biology, University of Helsinki, Helsinki, Finland; 4 Experimental Interaction Ecology Group, German Centre for Integrative Biodiversity Research (iDiv) Halle-Jena-Leipzig, Leipzig, Germany; 5 Institute of Biology, Leipzig University, Leipzig, Germany; 6 Natural History Museum, London, UK; 7 Dresden University of Technology, Dresden, Germany; 8 JUNIA, Health & Environment, Team Environment, Lille, France; 9 Laboratory of Nematology, Wageningen University, Wageningen, the Netherlands; 10 CNRS, Université Paul Sabatier III, Toulouse, France

**Keywords:** Environmental science, Global change, Soil biology

## Abstract

Identifying the main threats to soil biodiversity is crucial as soils harbor ∼60% of global biodiversity. Many previous meta-analyses investigating the impact of different global changes (GCs) on biodiversity have omitted soil fauna or are limited by the GCs studied. We conducted a broad-scale meta-analysis focused on soil fauna communities, analyzing 3,161 effect sizes from 624 publications studying climate change, land-use intensification, pollution, nutrient enrichment, invasive species and habitat fragmentation. Land-use intensification resulted in large reductions in soil fauna communities, especially for the larger-bodied groups. Unexpectedly, pollution caused the largest negative impact on soil biodiversity - particularly worrying due to continually increasing levels of pollution and poor mechanistic understanding of impacts relative to other GCs. Not all GCs and stressors were detrimental; organic-based nutrient enrichment often resulted in positive responses. Including soil biodiversity in large-scale analyses is vital to fully understand the impact of GCs across the different realms.

## Highlights

• Pollution causes the largest negative impact on soil fauna communities
• Land use intensification and climate change also negatively impacted communities
• GC impacts rarely varied with body size, and taxa groups often responded similarly
• Long-term stressors had no greater impact on soil biodiversity than short-term ones

## Graphical abstract

**Figure:**
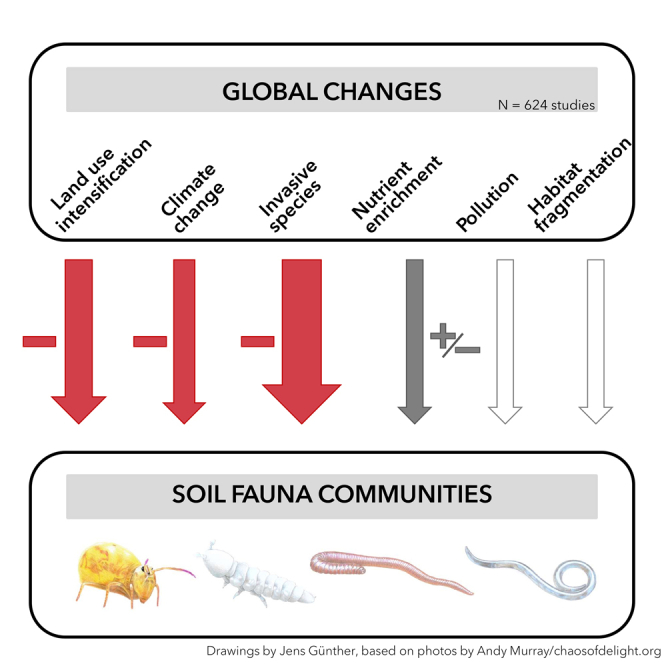

## Introduction

Soil fauna communities represent a significant fraction of global biodiversity, being composed of an extremely diverse set of invertebrate taxa, including earthworms, springtails and nematodes.^1^^,^^2^ Through their activity and their biotic interactions with each other as well as with plants and microbes, soil fauna are essential to nutrient recycling,^3^^,^^4^ soil fertility,^5^ and water infiltration^6^ among other ecosystem functions. They further represent important food source for aboveground biodiversity, with numerous invertebrates and vertebrates depending on this resource.^7^

Human impacts, such as climate change, land use intensification, invasive species and nutrient enrichment (hereafter referred to as global changes; GCs) are causing unprecedented changes in biodiversity, including shifts in taxonomic diversity,^8^ abundances of taxa groups,^9^ and community structure.^10^ These changes can occur at local scales and global scales.^10^^,^^11^^,^^12^^,^^13^ However, investigations of the impacts of GCs across large-scales are often focused on aboveground biodiversity,^2^ despite the fact that soil biodiversity does not respond in the same way as biodiversity in other systems to human impacts.^14^ Therefore, an important step in reaching better predictions of the consequences of human impacts for global biodiversity is through the incorporation of soil biodiversity. Unfortunately, to date, the majority of the global-scale work investigating impacts on soil biodiversity have focused on soil microbial communities^15^^,^^16^ (but see Peng et al.^17^) resulting in a lack of knowledge surrounding the highly diverse soil fauna communities.

Understanding the impact of such GCs on biodiversity in a generalizable way is important to be able to avoid and counteract potential biodiversity changes using scientifically sound policy and land use management.^18^^,^^19^ Meta-analyses and synthesis studies have become a popular way to investigate and compare GC effects.^8^^,^^18^^,^^20^^,^^21^^,^^22^^,^^23^^,^^24^^,^^25^ Meta-analyses and synthesis studies often find that land-use change and climate change are the biggest threats to biodiversity,^13^^,^^18^^,^^26^^,^^27^^,^^28^ and invasive species are also significantly reducing biodiversity.^18^^,^^29^^,^^30^ However, other GCs, such as pollution, are often understudied in aboveground terrestrial biomes,^31^^,^^32^ despite their potential damaging effects^13^ and their ever-growing rates of use.^33^^,^^34^ Currently there is no unifying classification of GCs, in terms of categories or what processes and impacts are captured within each category,^35^ and studies have captured GC impacts by incorporating different stressors. This is especially the case for meta-analyses that have focused solely on soil fauna that tend to address only specific GCs within their analysis, such as climate change^36^ or invasive species.^29^ And while recent synthesis efforts may address multiple GCs (e.g., Peng et al.^17^), they are still limited in scope by focusing on different aspects of climate change and nutrient addition while leaving out other GCs such as invasive species.

GCs can impact local biodiversity through a range of different environmental stressors; for example, climate change resulting in decreased precipitation or increased temperature.^37^ However, stressors within a GC category may not have the same impact on soil biodiversity, in terms of both magnitude and/or directionality; for example, warming and drought, both climate change stressors, can have opposing effects on soil fauna communities.^17^ Conversely, stressors from different GCs may have similar impacts on biodiversity due to similar mechanisms, such as changes in temperature and pollutants that both impact the physiology and metabolism of an organism.^38^ Although the term “stressor” implies a negative effect, GC can result in both decreases and increases in biodiversity.^39^ As a result, here, we follow Orr et al.’s^35^ definition, where stressors are causing any detectable biological change, no matter the direction. Thus, investigating stressor effects, as well as broad-scale GC impacts, may shed light on biodiversity responses. Additionally, classifying GCs based on the similarities of the characteristics and traits of the environmental stressors may allow better predictions across a wider range of GCs.^38^ Potentially providing a framework for exploring potential mechanisms that explain biodiversity responses to certain stressors.^19^^,^^40^

Here, we consider whether the stressor has a “pulse” or a “press” trait (Table 1),^19^^,^^40^^,^^41^ while noting that no classification scheme exists for assigning such traits to individual stressors. “Pulse” stressors are discrete disturbances^19^ that may be extreme in nature and occur less often, such as droughts. Alternatively, pulse stressors may be less severe but occur more regularly; for example, tillage, which can have negative effects on soil fauna.^42^^,^^43^^,^^44^ There is some expectation that soil fauna would be less negatively impacted by pulse stressors given that soil can provide a buffer to protect against fluctuations in climate or adverse conditions,^36^^,^^45^ as well as a physical buffer for organisms not directly dwelling at the surface.^46^ A buffering effect of soil against the pulse stress is especially likely if the stressor does not directly impact the soil environment (e.g., harvesting of aboveground biomass^46^).

**Table 1:** The environmental stressors associated with the five GCs that were analyzed (habitat fragmentation lacked sufficient data to investigate the associated environmental stressors)

Global Change	Environmental stressor	Stressor trait
Climate change	CO_2_ increase	PRESS
	Temperature change	PRESS
	Water availability—drought	PULSE
	Water availability—flood	PULSE
Land-use intensification	Grazing	PRESS
	Organic versus conventional farming	PRESS
	Harvesting	PULSE
	Fire	PULSE
	Tillage	PULSE
Pollution	Metals	PRESS
	Pesticides	PRESS
Nutrient enrichment	Synthetic fertilizers	PRESS
	Ca-liming + wood ash	PRESS
	Compost	PRESS
	Manure + slurry	PRESS
	Mixture	PRESS
	Other organic fertilizers	PRESS
	Residue + mulch	PRESS
	Sludge (including biosolids)	PRESS
Invasive species	Aboveground animal	PRESS
	Belowground animal	PRESS
	Plants-non-woody	PRESS
	Plants-woody	PRESS
Habitat fragmentation	NA	NA

All environmental stressors were classified as either a “press” stressor or a “pulse” stressor.

“Press” stressors are more continuous in nature than pulse stressors.^19^ Therefore, after a certain time frame, press stressors result in gradual species loss.^46^ For example, pollutants can generally be considered as press stressors in soils. Pollutants, such as heavy metals resulting from industrial or urban activities or persistent pesticides, can remain in the soil for centuries because they are not degraded.^47^^,^^48^ And although several pesticides are degraded in shorter time frames, their regular applications over the crop season may represent a press stressor for soil communities. Over longer time frames, it is likely that press stressors would change soil physico-chemical properties, resource availability, and habitat structure, as is seen with invasions of new species that directly affect the soil environment^49^ or with additions of organic amendments (such as manure, compost, and sludge).^50^ This eventually can change the soil environment strongly enough to cause compositional changes in soil fauna communities.

This expectation of stress effects on soil biodiversity is not necessarily in line with effects seen in aboveground communities, where organisms may be more directly impacted by pulse stressors due to having a limited number of habitats that can provide a buffer.^51^ For example, droughts can have immediate consequences for the physiology of an aboveground organism, resulting in mortality.^52^^,^^53^ Alternatively, harvesting aboveground biomass removes or degrades either a part or all the habitats required for aboveground organisms, thereby also reducing populations.^52^ Therefore, using the framework of pulse versus press events, and determining the differences in impact mechanisms in the GCs and environmental stressors, we can start to see the importance of ensuring the explicit inclusion of soil biodiversity into GC research.

The impacts of the environmental stressors are likely to be context dependent,^37^ for example, depending on the habitat type.^54^ As soil pH plays a key role in the diversity of many soil organisms,^55^^,^^56^ it may also influence the response of soil fauna to GCs and stressors. Impacts may also depend on the soil taxa being studied.^36^^,^^57^^,^^58^^,^^59^ Due to the huge diversity within the soil, soil organisms are often classified into three body size categories from micro-fauna (<100 μm), to meso-fauna (>100 μm to 2 mm) up to macro-fauna (>2 mm).^60^ Within these groups, it has been assumed that traits, such as microhabitat requirements, dispersal capabilities, and reproductive rates, are sufficiently similar to influence invertebrates’ responses to external pressures.^61^^,^^62^ Larger organisms may be more sensitive to the impacts of stressors than micro- and meso-fauna,^42^ because they have longer generation times and require larger microhabitats.^63^ As most soil micro-fauna is considered aquatic, their responses may also differ from those seen in meso- and macro-fauna.^60^

We conducted a meta-analysis to compare the effects of six GCs (climate change, land-use intensification, pollution, nutrient enrichment, invasive species, and habitat fragmentation) and their associated environmental stressors on micro, meso, and macro soil-fauna. We hypothesize that pulse stressors will have less impact on soil biodiversity than press GCs and stressors. Given that different contexts are likely to affect the impact of GCs and their environmental stressors, we hypothesize that impact of GCs and stressors will vary across the different body size categories, with macro-fauna showing the largest responses.

## Results

### Dataset overview

**Figure 1:**
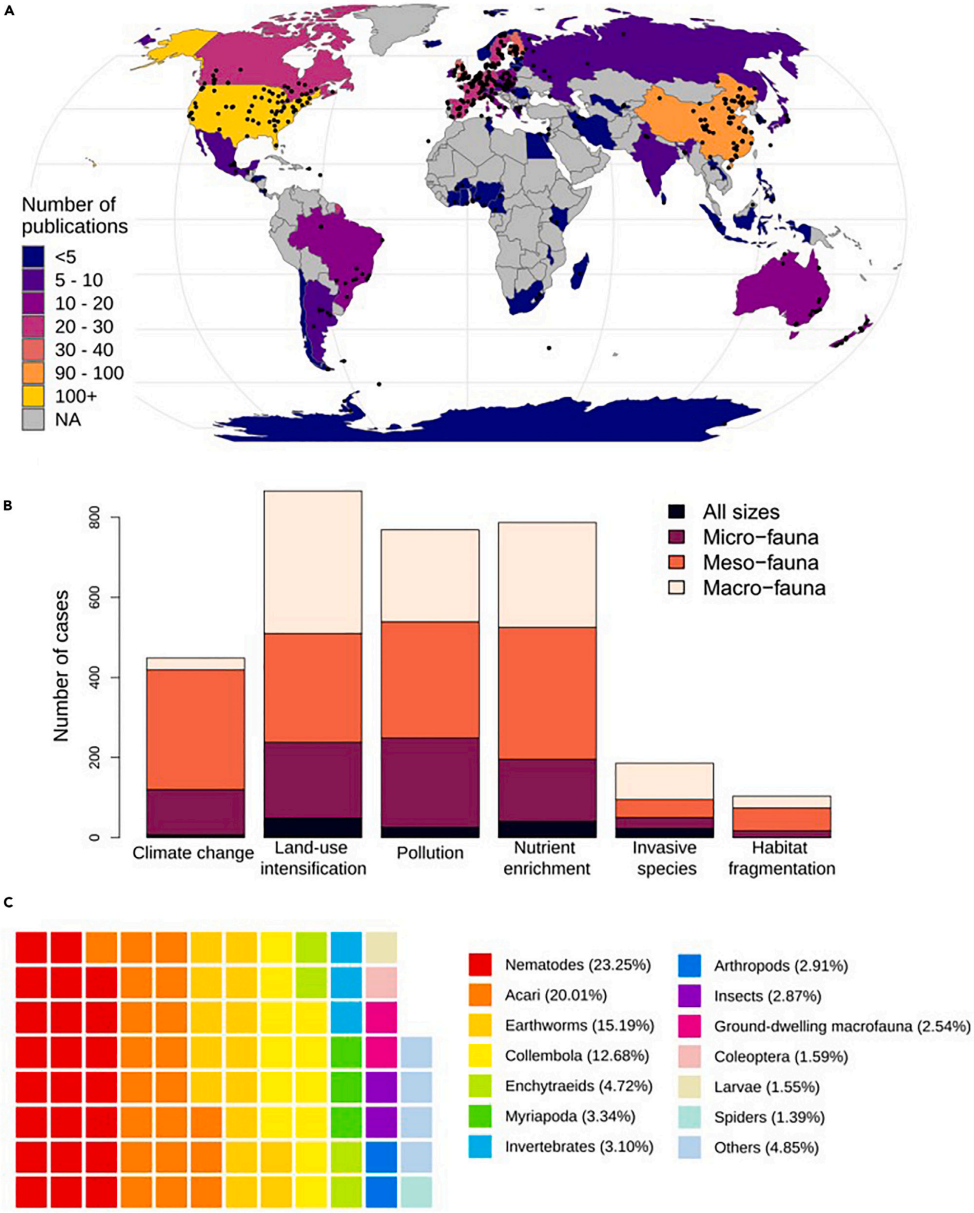
Distribution of studies and cases included in the meta-analysis (A) Spatial distribution of publications used in the analysis. Colors show the number of studies from each country that were included in the analysis. Gray areas indicate countries where no studies had been conducted that were included in this analysis. Black dots indicate location of studies where GPS coordinates were available (84% of studies). (B) The number of cases (i.e., an effect size with associated information on GC, environmental stressor, and other information) classified as each GC and each body size category. (C) Taxonomic distribution of cases included in this analysis. Colors distinguish different taxonomic groups only. Taxonomic group assignment was based on information provided in the original publication, and thus are at different taxonomic levels but classified based on the lowest possible taxonomic level (e.g., “Invertebrates” when the publication only referred to data at no specific level, versus Arthropods where cases could only be identified to phylum level, and “Insects” when cases were specified to class level). Data were only suitable when the community was measured above family level (except in the case of Enchytraeids). “Others” refers to 10 other groups. Groupings were based on Global Soil Biodiversity Atlas (GSBA;^64^) (Table S2).

In total, 3,161 cases were available for modeling from 624 published articles (see Figure S2 for PRISMA [preferred reporting items for systematic reviews and meta-analyses] diagram; Figure S3; see supplementary references). The cases were distributed across the globe; however, large numbers of cases were from the USA (100 papers and 15.5% of cases) and China (96 papers and 11.2% of cases) (Figure 1A). Land-use intensification (*n* = 876), pollution (*n* = 769), and nutrient enrichment (*n* = 789) were the most represented GCs, with habitat fragmentation (*n* = 104) having the least number of cases. The majority of cases (1,068 cases) are from woody habitats (either natural or plantation) and 1,006 cases are from agricultural systems. Grasslands (from any biome, including both natural and semi-natural) constituted 695 cases. Most cases were in relation to meso-fauna, which included micro-arthropods (*n* = 1,293). However, macro-fauna and micro-fauna were also well represented (*n* = 1,001 and *n* = 731, respectively; Figure 1B). Nematodes were the most well-studied taxonomic group, accounting for 23.25% of the cases, followed by Acari and earthworms (20.01% and 15.19%, respectively; Figure 1C). Just under 95% of the papers contained data from field experiments.

### Main GC model

**Figure 2:**
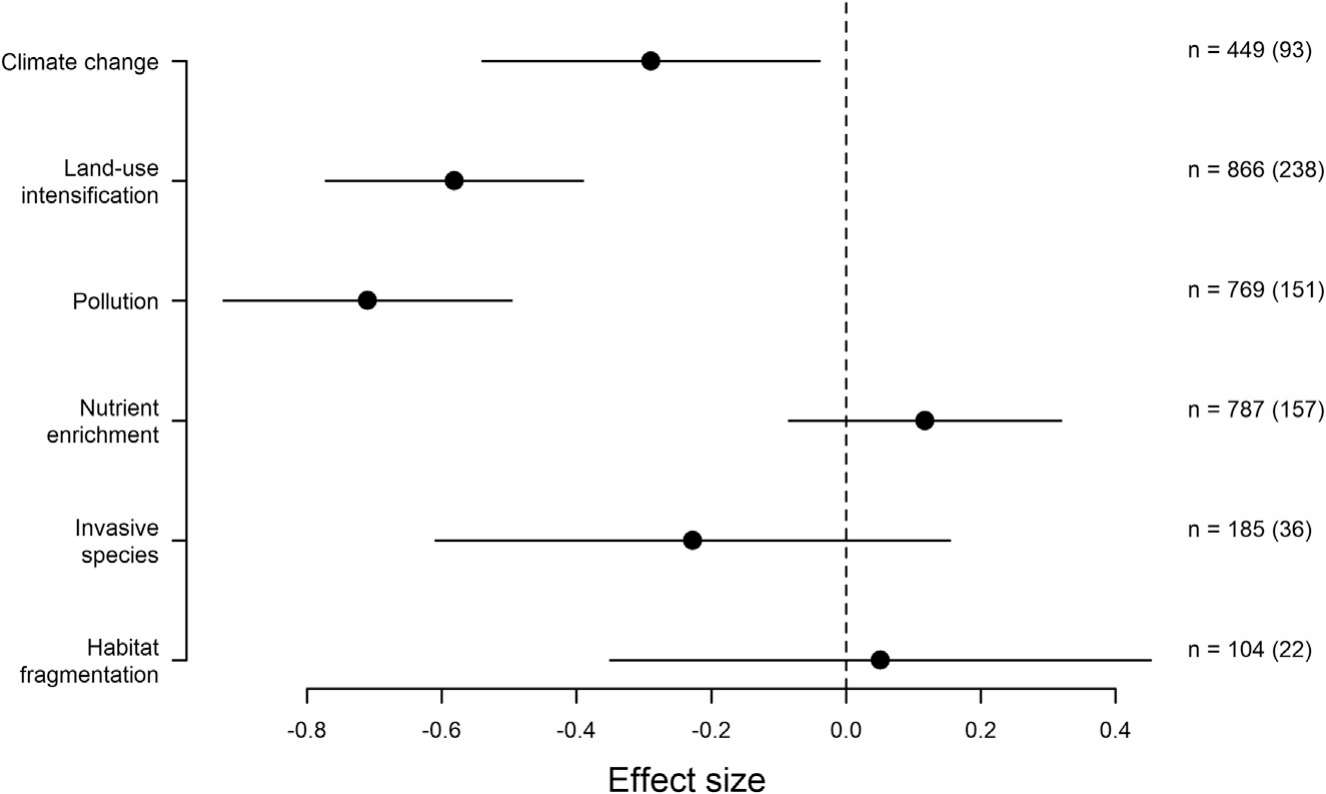
Change in soil communities in response to global changes (GCs) Hedges’ g was used as the effect size. Negative effect sizes indicate that the GC causes a reduction in biodiversity (measured using abundance, biomass, taxonomic richness, and Shannon’s diversity metrics), and a positive effect size indicates an increase in biodiversity. Error bars indicate 95% confidence intervals. Effect sizes where error bars do not cross the dashed vertical zero line, are significantly different from zero. The values of *n* indicate the number of cases of each GC in the model, with values in parentheses indicating the number of publications.

For the main model with the six main GCs, body size was removed from the model, as both the interactive and the main effect. Thus, the effect of the different GCs was the only remaining term in the model (Figure 2). The I^2^ of the model was 86.39% (percentage of the total variability due to true between-studies variability). 48.50% of the total variance was estimated to be due to between-cluster heterogeneity and 36.87% due to within-cluster heterogeneity. Only 1.01% of the total variance was due to the crossed effects of measurement type, indicating minimal heterogeneity as a result from the different community metrics of abundance, biomass, taxonomic richness, and Shannon’s diversity, indicating that it is less likely that the modeling approach used hid directionality shifts of the different community metrics to GC impacts. The remaining 13.61% was sampling variance.

Pollution had a significant negative impact on biodiversity (here we use the term “biodiversity” to refer to the soil community, when accounting for the different community metrics—abundance, biomass, taxonomic richness, and Shannon’s diversity—used in the analysis), the largest effect of all the GCs (estimate = −0.71; ±95% CIs = −0.92, −0.50; *p* value < 0.0001). Land-use intensification (estimate = −0.58; ±95% CIs = −0.77, −0.39; *p* value = < 0.0001) and climate change (estimate = −0.29; ±95% CIs = −0.54, −0.04; *p* value = 0.01) also had a significant negative effect, while habitat fragmentation, invasive species, and nutrient enrichment did not have a significant effect.

### Environmental stressor models

**Figure 3:**
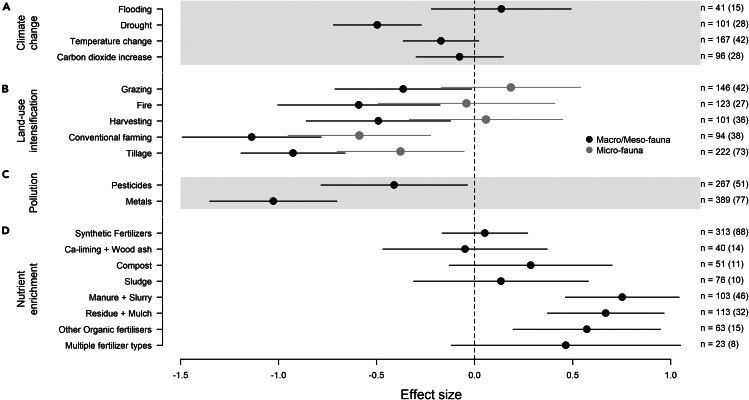
Change in soil communities in response to environmental stressors Change in soil communities in response to environmental stressors associated with (A) climate change, (B) land-use intensification, (C) pollution, and (D) nutrient enrichment. In (B), the estimate for macro- and meso-fauna is shown in black, and micro-fauna in gray as body size remained as an additive term in the model. Hedges’ g was used as the effect size in all models. Negative effect sizes indicate that the environmental stressor causes a reduction in biodiversity (measured using abundance, biomass, taxonomic richness, and Shannon’s diversity metrics), and a positive effect size indicates an increase in biodiversity. Error bars indicate 95% confidence intervals. Effect sizes where error bars do not cross the dashed vertical zero line, are significantly different from zero. The values of *n* indicate the number of cases of each stressor in the model, with values in parentheses indicating the number of publications. Gray shading is for enhancing readability only.

When focusing on the environmental stressors model relating to climate change, body size was removed as an effect from the model (both as an interactive effect and a main effect). The only climate change environmental stressor that had a significant effect on biodiversity was the detrimental impact of drought (estimate = −0.52; ±95% CIs = −0.76, −0.28; *p* value < 0.0001; Figure 3A). The effects of CO_2_ increase and temperature change (where 158 of the 167 effect sizes were based on increasing temperatures), although trending toward a negative impact, were not significant. The impact of increased water (absolute amount, rates, or number of events) through floods trended toward a positive impact on biodiversity but, again, was not significant (although also had the least amount of data, *n* = 41 cases).

For the environmental stressors model investigating different stressors related to land-use intensification, the effect of body size remained in the model, although as an additive effect only. Overall, macro-fauna was more negatively impacted by each of the land-use intensification stressors, while micro-fauna were impacted less (estimate = 0.55; ±95% CIs = 0.24, 0.86; *p* value < 0.001). Meso-fauna was not significantly different from macro-fauna, and thus was not presented separately (Figure 3B).

For both macro-/meso- and micro-fauna, the change from an organic system to an inorganic system had the biggest negative impact on biodiversity (estimate = −1.14; ±95% CIs = −1.49, −0.78; *p* value < 0.0001, for macro-fauna), with the increase in tillage (i.e., comparing reduced tillage practices to conventional tillage) having the second biggest negative impact (estimate = −0.93; ±95% CIs = 1.19, −0.66; *p* value < 0.0001, for macro-fauna). The impact of increased fire (intensity or frequency), harvesting, and grazing also had significant negative impacts, although to a lesser extent (and not a significant effect when adjusting environmental stressor estimates for micro-fauna).

Within the model focused on pollution stressors, the main and interactive effect of body size was removed from the model. The effect of pollutant type was significant, with both pesticides (estimate = −0.41; ±95% CIs = −0.78, −0.04; *p* value = 0.03) and metals (estimate = −1.03; ±95% CIs = −1.35, −0.70; *p* value < 0.0001) having a significantly negative effect on soil fauna communities (Figure 3C). Further inspection of the raw effect sizes, when accounting for different sources of metals and pesticides (using FAO [Food and Agriculture Organization] categories;^47^) show that there was variation of the effect sizes within each category (Figure S4). Notably, effect sizes of metals from mining/smelting demonstrate the greatest variation, often being more negative.

For the nutrient enrichment model, as with most of the models, the body size variable was removed as both an interactive and main effect, leaving just the impact of different nutrient enrichment stressors (Figure 3D). Of the 8 different stressors, five did not have any significant impact on biodiversity (synthetic fertilizers, Ca-liming + wood ash, compost, sludge, and multiple fertilizer types) but all trended toward a positive impact, except Ca-liming + wood ash. The impacts of manure + slurry, other organic fertilizers, and residue + mulch, were all similar, and all significantly positive (estimate = 0.75; ±95% CIs = 0.46, 1.04; *p* value < 0.001, estimate = 0.57; ±95% CIs = 0.20, 0.95; *p* value = 0.003, estimate = 0.67; ±95% CIs = 0.37; 0.97, *p* value < 0.001, respectively).

For the invasive species environmental stressors model, as with the pollution model, both the terms for the body size and the different types of invasive species were removed from the model completely. However, in line with the main model, the overall intercept of the models was not significantly different from zero (estimate = −0.15; ±95% CIs = −0.55, 0.25; *p* value = 0.47).

### Taxonomic, habitat type, and soil pH models

**Figure 4:**
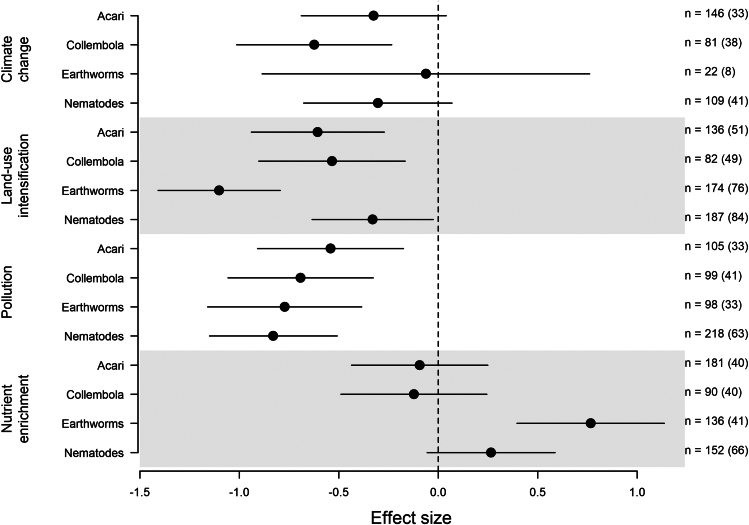
Change in four soil taxa groups (Acari, Collembola, earthworms, and nematodes) in response to four global changes (GCs) Hedges’ g was used as the effect size. Negative effect sizes indicate that the GC causes a reduction, and a positive effect size indicates an increase in that taxa group (measured using abundance, biomass, taxonomic richness, and Shannon’s diversity metrics). Error bars indicate 95% confidence intervals. Effect sizes where error bars do not cross the dashed vertical zero line, are significantly different from zero. The values of *n* indicate the number of cases of each taxa group within each GC in the model, with values in parentheses indicating the number of publications. Gray shading is for enhancing readability only.

The final taxonomic model (containing Acari, Collembola, earthworms, and nematodes data) retained the interaction between the taxonomic group and the GC. Just over half of the groups showed a significant negative decline in biodiversity with the GCs (9 coefficients out of 16; Figure 4; Table S3). Only earthworms showed a significant increase in biodiversity with the impact of nutrient enrichment, which contrasted with the other three taxonomic groups that showed no significant impact from nutrient enrichment. A similar model investigating the interaction between taxonomic group and environmental stressor showed that while the magnitude of the impact of each stressor varied across the groups, the directionality of the significant effect sizes was consistent (Figure S5).

Finally, for the habitat model and the soil pH model, where the model contained the habitat type variable and soil pH (respectively), the six-level GCs variable, and the interaction between the two, both habitat type and soil pH were removed from the model from the interaction and the main effect.

### Publication bias

The funnel plots show the asymmetry in the data (discussed further below), particularly in the case of land-use intensification (Figure S6). In addition, in the data for the main model, as well as the data for the models focused on the stressors of land-use intensification, nutrient enrichment, and to a lesser extent climate change, there is a lack of small effect sizes that have high precision (i.e., the tip of the funnel) (Figure S6).

Using the approach given in Nakagawa et al.,^65^ all models showed bias related to small-study effects but no evidence of decline effects/time-lag bias, despite the possibility of a decline effect over time occurring within the wider ecological literature,^66^ we found no effect of publication year on any of the models. So, regardless of the small-scale study effects present, the effect sizes that are used within the analysis are representative of the field over time.

Accounting for the biases in the dataset resulting from the small-study effects had minimal impact on the main messages. Pollution still caused the largest negative decline in biodiversity, followed by land-use intensification (Table 2). However, when adjusting for bias, climate change no longer had a significant impact, and nutrient enrichment had a positive significant effect (Table 2). For the environmental stressor models within each GC, the biggest changes were within the land-use intensification model, where once adjusted for the biases, only organic vs. inorganic and tillage had a significantly negative impact on biodiversity. The estimates for all other environmental stressors no longer remained significant (Table 2). For climate change, drought still had a significant negative impact on biodiversity (Table 2). For nutrient enrichment, manure + slurry and residue + mulch still had a significant positive impact, although the “other organic fertilizers” no longer remained significant (Table 2). For invasive species and pollution, where the models were intercept only models, the intercept for pollution remained significant, whereas the non-significant intercept for invasive species became positively significant when bias was accounted for (Table 2).

**Table 2:** Adjusted and unadjusted effect sizes (Hedges’ g) and CIs for all models (GCs and environmental stressors)

	GC/Environmental Stressor	Adjusted effect size	Adjusted CIs	Unadjusted effect size	Unadjusted CIs
GC main model	Climate change	−0.13	[−0.38,0.12]	−0.29	[−0.54,−0.04]
	Land-use intensification	−0.41∗	[−0.60,−0.22]	−0.58∗	[−0.77,−0.39]
	Pollution	−0.51∗	[−0.72,−0.29]	−0.71∗	[−0.92,−0.50]
	Nutrient enrichment	0.30∗	[0.10,0.51]	0.12	[−0.08,0.32]
	Invasive species	−0.11	[−0.48,0.26]	−0.23	[−0.61,0.15]
	Habitat fragmentation	0.18	[−0.21,0.57]	0.05	[−0.35,0.45]
Climate change	Flooding	0.31	[−0.05,0.68]	0.12	[−0.25,0.49]
	Drought	−0.31∗	[−0.55,−0.07]	−0.52∗	[−0.76,−0.28]
	Temperature change	0.006	[−0.22,0.21]	−0.20	[−0.41,0.02]
	CO_2_ increase	0.07	[−0.17,0.32]	−0.10	[−0.34,0.14]
Land-use intensification	Grazing	0.07	[−0.27,0.30]	−0.36∗	[−0.71,−0.01]
	Fire	−0.10	[−0.49,0.30]	−0.59∗	[−1.00,−0.18]
	Harvesting	−0.01	[−0.36,0.34]	−0.49∗	[−0.86,−0.12]
	Organic vs. Conventional	−0.67∗	[−1.01,−0.33]	−1.14∗	[−1.49,−0.78]
	Tillage	−0.41∗	[−0.67,−0.15]	−0.93∗	[−1.19,−0.66]
Pollution	Pesticides	−0.37∗	[−0.73,−0.002]	−0.41∗	[−0.78,−0.04]
	Metals	−0.91∗	[−1.23,−0.59]	−1.03∗	[−1.35,−0.70]
Nutrient enrichment	Synthetic fertilizers	−0.18	[−0.42,0.06]	0.05	[−0.16,0.27]
	Ca-liming + wood ash	−0.27	[−0.70,0.16]	−0.05	[−0.47,0.37]
	Compost	0.01	[−0.43,0.44]	0.29	[−0.13,0.70]
	Sludge (including biosolids)	−0.07	[−0.53,0.38]	0.13	[−0.31,0.58]
	Manure + slurry	0.48∗	[0.16,0.79]	0.75∗	[0.46,1.04]
	Residue + mulch	0.43∗	[0.11,0.75]	0.67∗	[0.37,0.97]
	Other organic fertilizers	0.34	[−0.05,0.73]	0.57∗	[0.20,0.95]
	Multiple fertilizer types	0.21	[−0.39,0.81]	0.47	[−0.12,1.05]
	Invasive species (all stressors mean)	0.52∗	[0.05, 0.98]	−0.15	[−0.55, 0.25]

Effect sizes were adjusted using methods in a study by Nakagawa et al.^65^ to account for small-study effects.

∗ Effect sizes indicate statistically significant from zero.

## Discussion

We examined the impact of a wide array of GCs on a wide range of soil fauna groups, considerably extending the scope of previous meta-analyses that have focused on soil fauna.^17^^,^^29^^,^^43^^,^^67^ In doing so, we identified that pollution and land-use intensification had the greatest negative impacts on soil biodiversity (abundance, richness, biomass, and Shannon’s diversity). Climate change also had a negative, albeit smaller, impact, although after adjusting for biases within the publications, this significant effect was lost. For GCs where environmental stressor was a significant predictor, there was no strong indication that press stressors had greater impact on soil communities, except in the case of land-use intensification. Overall, the effect of the GCs did not vary with context, as there was no effect of habitat type on the estimate, and rarely was there an effect of the different body size classes. Studying the impact of pollutants may not be possible with aboveground organisms, due to the lack of primary studies focused on pollutant impacts,^28^ but by focusing on soil biodiversity, we are able to understand how detrimental environmental pollution is relative to all other GCs. The only GC that comes close is land use intensification.

### Climate change

Climate change is often referred to as one of the biggest threats to biodiversity.^13^^,^^27^^,^^68^ In this meta-analysis, despite the general decline in soil fauna biodiversity, climate change was not the greatest threat. However, a more nuanced view may be needed. Based on aboveground organisms, it might be expected that temperature changes would result in a decline in biodiversity.^21^^,^^27^^,^^28^^,^^69^ Although recent projections indicate that by 2100, soils could be 4.5°C warming to 1 m depth,^70^ our results support the hypothesis that soils can buffer the impacts of temperature change at least on the relatively short-term (also found in Peng et al.^17^ and Cordero et al^71^). Indeed, the observed decline was due to clear negative effects of reduced water availability, which has previously been shown in other meta-analyses to be a strong driver of soil biodiversity.^17^ This is cause for concern, as areas of climate change-induced drought are increasing.^72^ Thus, it is likely that the detrimental impact of drought, as well as depleted soil fauna community will result in reduced ecosystem function and services in those areas,^73^^,^^74^ thus human populations in those areas may experience 2-fold impacts.

Although we categorized drought as a pulse stressor and expected less impact on soil biodiversity than changes in temperature and CO_2_, it was impossible to quantitatively compare the strength or duration of the drought manipulations (relative to the normal levels) across the different studies with the information available. For example, the shortest treatment duration was 45 days, but involved 100% water removal for the full duration.^75^ Whereas the longest treatment (13 years from the start of the treatment until sampling of the soil fauna presented in the original publication^76^) only had drought conditions for 1 to 2 months a year, reducing soil moisture by 35–50% during this time. Thus, determining intensity across such a variety of manipulations is problematic. If the drought manipulations were too severe (too extreme or too long), there is the potential for the buffering capacity of the soil to be significantly reduced,^45^ resulting in biodiversity loss.^71^ In a similar vein, the temperature manipulations may also not be an appropriate length to be considered a press stressor. Blankinship et al.^36^ found that longer climate-change treatments resulted in more significant effects, although in their study, the effect was positive.

Considering the length of the treatment application using an alternative modeling structure may provide further insights. However, often temperature changes occur simultaneously with water-regime changes, and previous studies have demonstrated that if soil water is available for soil organisms, the effects of other stressors, such as temperature increases, can be buffered against.^17^^,^^77^^,^^78^ Thus, it would be prudent to consider the synergistic effects of many of the stressors simultaneously (discussed further below) to fully understand the most detrimental components of climate change for soil biodiversity.

### Land-use intensification

Land-use intensification was the second strongest GC impacting soil fauna communities. When looking at the different environmental stressors of land-use intensification, all were significantly negatively impacting soil fauna, although the shift from an organic system to a conventional system, a press stressor, and the intensification of tillage practices, a pulse stressor, resulted in the greatest decrease. Previous meta-analyses have also found intensification of tillage practices to be detrimental to soil fauna.^43^^,^^44^ Tillage causes direct mortality and destruction of habitat, which exposes soil organisms to predators.^79^ However, effects can also be indirect and longer term, through a reduction of soil structure (limiting access to nutrient resources, especially for smaller organisms, and increasing exposure to an environment that may result in desiccation), changes in plant community composition altering resource quality, and reduction in soil organic matter at the surface.^44^ This highlights the weakness of using just a single trait of an environmental stressor, and shows the need to consider more than one characteristic in future studies.^19^^,^^40^

It is difficult to pin-point the exact agricultural practices that promote soil biodiversity in organic systems, because organic farming specifications depend on countries, crops, and other factors.^80^^,^^81^ Based on previous meta-analyses,^82^ the decline in biodiversity may be the result of the shift in nutrient addition regimes, as it is probable that the strong decline was most associated with the change from organic fertilizers and reduction in mulch. The addition/retention of mulch (plus other soil amendments) and organic fertilizers were particularly beneficial in this analysis, as well as others.^82^ Individually, these components promote soil biodiversity (see below), and in concert could provide even greater benefits to the soil community. However, as the addition of pesticides (singularly within the pollutants category) also significantly negatively impacted soil communities, the shift away from high pesticide use cannot be ruled out as a contributor to the positivity of organic agricultural systems. What is interesting to note is the effect of the different land-use intensification types interacted with the body size of the organisms, with macro-fauna (predominantly comprised of earthworms) being most negatively impacted. Given that earthworms mostly benefitted from the addition of nutrient enrichment, there is some indication that, at least for earthworms, the nutrient enrichment aspect of organic agriculture may be the most important (Figure 4).

### Pollution

Of critical concern is the strong impact of pollution on soil biodiversity. We found that both metals and pesticides had a strong impact on soil biodiversity. The fact that metals did have such strong impacts on biodiversity is not necessarily surprising. Typically, studies focused on metal pollutants were conducted in landscapes with a long history of pollution associated with mining and smelting activities.^32^^,^^83^^,^^84^ The sustained toxicity of the soil may not only result in direct mortality but may also prevent recovery of the soil fauna populations,^46^ thus resulting in large soil biodiversity loss. Additionally, authors often investigated gradients of metal pollution across a large range of concentrations,^85^^,^^86^^,^^87^ and given that our meta-analysis cases were based on the most intense comparison, this may also result in the larger effect size for metal pollutants. Indeed, pesticide studies mostly applied pesticides at the recommended application rates that may represent much less intense levels than metal pollution gradients. In addition, although metals in soil are “press” stressors, many pesticides can be degraded over time so their classification as a “press” stressor may not be correct for all the studies included here. However, as long-term studies of pesticide degradation times are lacking for all the types of pesticides covered by this meta-analysis,^48^ a dichotomy in the response of press/pulse stressors may have held true if we were able to classify pesticides into their most appropriate press/pulse categories.

Additional insight would also be likely if we were able to further determine or classify the concentration of pollutants or, alternatively, compare different types of pollutants at equivalent levels. In traditional dose-effect theory in ecotoxicology, the dose of the pollutant is typically one of the strongest drivers of biological response. For instance, Chauvin et al.^23^ focused their meta-analysis solely on nematodes and heavy metal pollutants and were able to determine that increasing concentrations of heavy metals resulted in more pronounced declines in biodiversity. However, Beaumelle et al.^39^ found that across terrestrial and aquatic systems, decomposers’ community responses to pollutants did not change with pollutant levels but were generally negative. The community-level response may be affected by combined direct toxic and indirect effects mediated by changes in species interactions, yielding not so straightforward dose-response curves at this ecological scale. Moreover, primary studies often addressed the combined effects of multiple pollutants that may have interactive effects causing even further deviation from classical dose-effect theory.^88^

In general, the impact of pollutants on terrestrial biodiversity is particularly understudied,^28^^,^^32^^,^^89^ and although there are several studies on the impact of pollutants on soil biodiversity, there are still large gaps in our knowledge.^32^ As there was a limited number of pollutant stressors that could be analyzed in this meta-analysis, we hope that future studies will address other types of pollutants (such as microplastics, hydrocarbons, and emerging pollutants^32^) in order to understand the role they may play in biodiversity change. Given the clear negative effects reported here, our results thus call for more research focusing on the mechanisms that lead to community-level responses, on the impacts of a broader range of pollutant types, and more consistent ways of standardizing pollutant levels in primary studies. Our results also reinforce recent calls for coordinated global monitoring and policy-actions based on scientific evidence and call for a proper integration of soil biodiversity in such initiatives.^33^^,^^90^

### Nutrient enrichment

Nutrient enrichment did not have an overall significant effect on soil fauna communities. However, the effects on soil fauna did vary depending on the nutrient-based environmental stressors. Given that all nutrient stressors were considered to be press stressors, this once again highlights that the use of just a single trait does not capture the complexity of the stressors, especially when it is unclear how long each nutrient type remained in the soil before degrading. Organic-based amendments and fertilizers increased soil biodiversity, and several mechanisms may explain this pattern. It may be due to the increased carbon in the soil.^91^ Liu et al.^82^ found that inorganic nitrogen fertilizers simplified nematode communities but organic fertilizers (for example, crop residue) increased carbon in the soil and had positive impacts on the nematode community. However, it may also be because the organic-based fertilizers provide the nutrient resources at a slower rate,^92^ as well as create micro-habitats for a variety of soil organisms.^50^ The fact that we did not find a significant decline in biodiversity with synthetic fertilizers was somewhat surprising, although this result has been found in another meta-analyses.^17^

Beaumelle et al.,^39^ in their meta-analysis, found that nutrient addition had positive effects on soil biodiversity at low doses, and effects became negative at higher concentrations. Thus, if concentration amount was not taken into consideration, the overall mean effect size was neutral. Additionally, de Graaff et al.^44^ found that synthetic fertilizers had a significant negative effect on soil fauna but were no longer significant when the duration of the application was increased (>5 years). Similar results were found in Murchie et al.,^93^ where long-term application of synthetic fertilizers did not negatively impact earthworms. Both hypothesized that any effect on soil biodiversity was indirect, as the increased nitrogen in the soil benefited the crops, and thus in the longer term the increased plant biomass in the soil resulted in increased carbon, ultimately benefiting the soil community.^94^ Without looking into the concentration and application rates, or the temporal aspects of the treatment, all of which are hard to standardize across such a wide variety of studies, we would not be able to test these mechanisms, but these may explain the lack of a significant decline with synthetic fertilizers.

### Context-dependency of the impacts

The effect of the GCs did not vary across different habitat types, soil pH, or organism body size classes for most of our analyses.

A number of GC meta-analyses have found no significant effect of habitat type. For example, Pressler et al.^95^ typically found no significant differences of fire effects on soil fauna communities across different habitats (forest, grassland, and shrubland, referred to as biomes). Similarly, Blankinship et al.^36^ found no effect of habitat type when investigating the effects of CO_2_ increase and warming but did find the effects of altered precipitation were greater when in forested habitats. Therefore, while the categorizations used for the different habitats may be masking effects, there are prior expectations that habitat type is less important when investigating GC impacts on soil fauna communities.

While many meta-analysis investigate the impact of GCs on soil properties such as pH,^49^^,^^96^ few use soil pH as a covariate within their models. In a meta-analysis investigating the impact of tillage on earthworms, Briones and Schmidt^43^ used pH within their models and found that effect sizes only differed when soil pH values were low (<5.5). Given that 75% of our effect sizes that had pH values were >5 (minimum = 2.9, maximum = 10.6, median = 6.2) it is unclear whether effects of pH would have been detectable in our analysis.

The lack of significance for body size was also highlighted when we analyzed only the four most abundant taxonomic groups (Acari, Collembola, nematodes, and earthworms), which represent all three body size classes. The patterns for nutrient enrichment were the most surprising, where earthworms responded differently from the other three taxa, driven by their strong positive response to “residue + mulch” and sludge (Figure S5). For the other GCs, there were limited differences. However, looking at community composition or functional and trophic groups instead of taxa or body size groupings may tease apart other context-dependencies.^82^^,^^97^^,^^98^ For example, previous work has shown various responses to environmental stressors and GCs depending on the different functional types of earthworms.^94^^,^^97^^,^^99^

In other analyses, community composition of soil microbial communities were more affected than metrics of alpha- and beta-diversity^15^^,^^82^ to different GCs. Incorporating such metrics of soil fauna communities may be possible, especially in the case of nematodes and earthworms, as communities are often reported at the level of functional types. In addition, the community metrics used in this meta-analysis may not have captured all the changes that occur to soil communities in invaded ecosystems, resulting in the non-significant changes. For example, Abgrall et al.^54^ established that soil communities responded more similarly within trophic groups, and accounting for trophic group (and habitat type) within the meta-analytic framework was needed to see the impact of invasive species on soil fauna abundance.

### Limitations and future directions

Across the entirety of the dataset, as well as within each GC, there was a distinct lack of positive large effect sizes, resulting in asymmetry. Yet, it is difficult to fully understand the type(s) of bias that has caused the asymmetry.^100^ It is most likely that the bias is a result of certain experiments never being published,^101^^,^^102^ through selective reporting from either authors or journals.^103^ However, it is possible that the GCs rarely have a positive effect on soil biodiversity. Thus, in that instance the “bias” is not a true bias, and in theory the results should not be adjusted, as done here. Although we are unable to establish the underlying cause of the bias, adjusting for the bias had minimal significant impact on the outcomes of the meta-analysis (Table 2). The results that did significantly change, either by no longer being a significant impact or becoming significant, were those with the least amount of data, and therefore least robust coefficients.

As far as we are aware, no meta-analysis has previously used trait characteristics of the GC, when investigating GC and stressor impacts. Using the “pulse” and “press” traits to describe the environmental stressors helped to introduce the mechanisms, and highlight the similarities, that may result in biodiversity change, regardless of the GC they are associated with. However, the lack of consistency in results may be due to the simplistic nature of using just a single trait for each stressor. Indeed, Rillig et al.^40^ proposed 30 different traits to characterize environmental stressors, a classification scheme that they found worked well when applied to a soil microbial experiment. Orr et al.^19^ further stated that stressors should not only be classified based on their traits but also sources, temporal overlap, mode of action, and co-tolerance to determine their similarities and aid predictions. Unfortunately, there is currently no classification scheme that assigns environmental stressors to such a wide range of different traits, which would be needed to fully realize the benefits of this approach.

As not all GCs and environmental stressors had enough data to enable in depth investigation, further questions remain as to the specific impacts of habitat fragmentation and invasive species on soil fauna. It would be expected that both habitat fragmentation and invasive species alter the soil enough to impact many soil organisms.^29^^,^^30^^,^^49^ Alternatively, more moderators may be needed to capture the heterogeneity (e.g., habitat type^54^), and/or more complex interactions between the moderators.^104^

The breadth of this meta-analysis provides us with insight and comparisons that have previously not been possible. However, it also reduces our ability to “zoom” into specific effects,^25^ such as different doses of GCs (stressor concentrations and amounts), temporal aspects, or community composition shifts. For example, by focusing only on pesticides in their meta-analysis, Beaumelle et al.^67^ were able to determine that the recommended dosage for commercially available pesticides applied to soil communities still resulted in biodiversity loss. Such analysis is not possible across many GCs and stressors due to the variety of treatments that can be applied, which may vary in intensity depending on landscape history. Future studies could focus on the duration of the treatment to ascertain whether this is a proxy for intensity across the different GCs and stressors, or how rates of change of the stressors may vary magnitude.^105^ Additionally, future work with a focus on additional diversity indices, such as community composition and beta diversity metrics, may also be insightful. We hope that by highlighting the relative lack of data, these gaps may be overcome in the future with additional studies.

One aspect of the study of GC that is being highlighted in the scientific literature as an important avenue of research is the potential impact of two or more GCs simultaneously.^17^^,^^106^^,^^107^^,^^108^ Many meta-analyses that looked at the additive and interactive effects of GCs on biodiversity have found that the interactions between GCs are important.^15^^,^^16^^,^^17^^,^^106^^,^^109^^,^^110^ Although it might be anticipated that the interaction between GCs may result in greater negative impacts than expected based on the singular GCs,^110^ there is the potential for the presence of two GCs to result in less of a negative impact than expected.^16^^,^^17^^,^^109^ Given the changing world that we are in, and that GCs rarely act singularly on biodiversity,^108^ this would be the most important avenue to study further, and is indeed possible within a meta-analysis framework when factorial data are present.^15^^,^^16^^,^^109^^,^^111^

### Conclusion

Overall, many of the GCs and environmental stressors studied here reduced soil biodiversity, irrespective of the body size of the organisms or habitat type. However, there are notable exceptions where a biodiversity increase occurred, namely with addition of more organic-based nutrient enrichments. Given the profound decline of soil biodiversity due to pollution, a GC that is understudied in aboveground literature, we emphasize the need to increase research on its impact across all realms. By classifying GCs and environmental stressors in terms of pulse or press traits, we were able to link the similarities between the different stressors in terms of their mechanisms with the ways they may impact soil fauna communities. As the responses of soil organisms to GCs and environmental stressors differed from published responses of aboveground organisms, soil biodiversity needs to be explicitly included into large-scale analyses of GC impacts.

## STAR★Methods

### Key resources table

**Table:** 

REAGENT or RESOURCE	SOURCE	IDENTIFIER
**Deposited data**		

Raw and analyzed data	This paper	https://doi.org/10.5281/zenodo.6903152

**Software and algorithms**		

Abstrackr	Wallace et al. 2012	https://abstrackr.cebm.brown.edu/
Web Plot Digitizer		https://apps.automeris.io/wpd/
metafor package	Viechtbauer 2010	https://doi.org/10.18637/jss.v036.i03
R	R Core Team 2021	https://cran.r-project.org/
Analysis Code	This paper	https://github.com/helenphillips/GCimpactsSB

### Resource availability

#### Lead contact

Further information and requests for resources and reagents should be directed to and will be fulfilled by the lead contact, Helen Phillips (helen.phillips@helsinki.fi).

#### Materials availability

This study did not generate new materials.

#### Data and code availability

All data have been deposited in a Zenodo repository (https://doi.org/10.5281/zenodo.6903152) and are publicly available as of the date of publication. Full reference list, including DOIs, are available in the Supplementary Materials. All original code is available from GitHub (https://github.com/helenphillips/GCimpactsSB) and is publicly available as of the date of publication. Any additional information required to reanalyze the data reported in this paper is available from the lead contact upon request.

### Method details

Six GCs were considered for the meta-analysis: climate change, land-use intensification, pollution, nutrient enrichment, invasive species, and habitat fragmentation. These were chosen based on the main drivers of biodiversity loss presented in other large-scale works.^13^^,^^18^^,^^112^ However, we note that these GC categories are not on the same hierarchy in terms of cause and effect but are inline with the literature, which has a lack of consistent terminology.^35^ For example, we include CO_2_ together with temperature change in the climate change category, as the primary literature often tests the direct effects of CO_2_ on biodiversity (e.g., FACE experiments), rather than as the driving factor (i.e., changing temperatures). We also note that we did not include ‘land use change/transformation’ as a GC, despite its prevalence within the literature. The number of possible land transitions across the globe, plus the need to rank the transitions by their intensity, which is not always possible, makes this specific portion of the literature not suitable for this meta-analysis framework. But given the importance of this aspect of GC we hope that such a meta-analysis is undertaken in the future. Instead, following the definition by IPBES, we only considered land use intensification and habitat fragmentation, but as separate categories to further distinguish them from land use transformation.

#### Literature search

A Web of Science search was performed on 15^th^ October 2018 searching all available literature in the Core Collection using a combination of search terms that captured groups of soil organisms ‘AND’ metrics of biodiversity ‘AND’ GC (see supplementary materials Data S1 for full list of search terms). This resulted in 24,979 records considered for screening (see Figure S2 for PRISMA diagram).

#### Screening of titles, abstracts, and main text

Titles and abstracts were screened for suitability. Suitable abstracts mentioned at least one group of soil fauna measured in at least one ‘control’ site, and one site impacted by a GC (either due to experimental design in the field, laboratory or greenhouse, or a gradient occurring across a landscape). To aid in screening of titles and abstracts, we used a machine-learning algorithm in the program Abstrackr^113^ alongside human-screening. While the abstracts and titles were being manually screened, all papers were being dynamically assigned confidence scores by Abstrackr. After the manual screening of 9,535 abstracts (of which 6,143 were irrelevant and 3,389 were included), the Abstrackr confidence score was 0.58 or under for the remaining 15,444 articles, a low enough value to indicate the remaining articles were not relevant for the meta-analysis. This cut-off value of 0.58 was chosen based on a quality control procedure in which we randomly sampled 5% of the records within each 0.1 band of confidence scores, and screened their titles to check that they ‘may be’ suitable or were “definitely not” suitable. The cut-off confidence score was then based on the point where the number of ‘definitely not’ suitable papers was the majority of the titles within a 0.01 band. Thus, the 15,444 articles were not considered further.

The full texts of the 3,389 papers with relevant abstracts and titles were then manually screened. In order to be suitable for the analysis the article needed to have (1) measured at least one soil fauna group (e.g., earthworms, macro-fauna, oribatid mites) with an alpha-based metric. Studies that focused on specific species, or manipulated communities were not included. (2) Captured the impact of one or several GCs according to our GC-specific inclusion criteria (see supplementary materials), and (3) presented the necessary data (mean values, variance, n’s) to allow us to calculate an effect size for the meta-analysis. Control sites didn’t have to have zero GC impact, instead they could be sites that were less impacted by the GC. However, it was important that the control and treatment sites were within the same type of land use and/or habitat (e.g., comparing the impact of GC within agriculture land or within forested land), to prevent confounding effect of land use change. There was no requirements for number of replicates, or plot size.

As no definition, catalog, or list exists of organisms considered ‘soil biodiversity’,^64^ soil fauna was determined based on sampling protocol. Suitable sampling methods included soil cores, hand-sorting excavated soil blocks, or mustard extraction. Pitfall traps on their own were not considered suitable, as these data are more representative of activity densities of ground-dwelling invertebrates.^114^ However, if the pitfall traps were associated with another method targeting the soil, they were considered suitable.^115^ Additionally, while soil organisms could be the target organisms or the non-target organism in pollution (specifically pesticide) studies, they were predominantly the non-target organism.

#### Data extraction

Data, such as the control and treatment means, variances, and sampling effort were extracted either from the text, tables, or figures using Web Plot Digitizer (https://apps.automeris.io/wpd/). To help ensure consistency in data extraction, all project members followed a data extraction protocol created at the start of the project (see supplementary materials Data S2). In addition, data extracted from nearly all articles were checked by a second individual.

For the control and treatment means, we extracted data related to shifts in the soil community. While we extracted a variety of different community metrics, in this analysis we focused on metrics of abundance, biomass (both measured as the total of the soil group being measured), taxonomic richness (either species or genus), and Shannon diversity (an abundance-based community evenness metric^116^), as they are standard measurements to investigate GC impacts,^18^^,^^36^^,^^43^ widely used (accounting for 95.78% of the data points we extracted), are considered as Essential Biodiversity Variables (“Community abundance”^117^) and are related to ecosystem function.^118^ Here, we use the term ‘biodiversity’ to refer to the soil community, when accounting for the different community metrics used in the analysis (see ‘Model Structure’ below).

When data were presented as a time-series, the last time point in the series was used for the control and treatment means to maximize independence.^49^^,^^119^^,^^120^ When a gradient of impacts was presented, e.g., different fertilizer levels, the most extreme level was used as the treatment. Data were extracted at the highest level of taxonomic resolution possible but above family level (except enchytraeids, which are an important group commonly reported only at family level). When presented as functional groups (as is often seen in nematode and earthworm studies), the data were pooled together into the taxonomic group.

Each study could provide more than one comparison between a control and treatment, henceforth referred to as a ‘case’. Most commonly, primary papers presented data for multiple community metrics (for example, abundance, biomass and species richness), different taxonomic groups, different GCs or environmental stressors, or from different habitat types. Each comparison was extracted as an individual case. Where a paper presented multiple GCs/environmental stressors, each was extracted independently from any other.

#### Global change variables

Each case was assigned to one of six main GCs (land-use intensification, habitat fragmentation, climate change, invasive species, pollution, and nutrient enrichment) based on our own classification system (as set out in the data extraction protocol) as well as the intention of the original paper. We considered nutrients and pollution separately, given that we had different hypotheses for their effects, and following Beaumelle et al.^39^ Here, pollution refers to a change in concentration of substances such as metals, pesticides, and other chemicals, although we acknowledge that pollution due to excess nutrients occurs as well. This classification also ensured that descriptions and motivations within the original paper was also followed, for example, so that a treatment that applied an input to increase nutrients and/or promote plant growth, was classified as a nutrient enrichment.

In addition, depending on the main GC, each case was also categorized with one environmental stressor (Table 1). The stressor depended on the main GC, but captured how the GC impacted the local biodiversity. The full list of stressors for all GCs, including those not used in the modeling, is provided in Table S1. Although the intensity of the treatment was extracted (e.g., rate of application), the treatments were too varied within and across the different stressors to harmonize or compare. Each environmental stressor was then categorized as either a ‘pulse’ or ‘press’ stressor (Table 1). This was based on the ecological understanding of the stressor in relation to what is expected at the global scale, as well as the majority of the data in the stressor (e.g., most temperature change was longer term, mean = 3.5 years of treatment, so was assigned as a press stressor, as opposed to shorter term ‘heat wave’ events that would have resulted in being assigned as a pulse stressor).

#### Predictor variables

For each case, data for model moderators, such as the taxonomic group, body size, soil pH, and habitat type were collected. Taxonomic names were harmonized to those used by the GSBA^64^ (Table S2). From the GSBA harmonization, each taxonomic group was assigned to a body-size category (micro-, meso-, macro-fauna) if that had not already been done by the original author. Body-size categories were also based on Orgiazzi et al..^64^ Microarthropods were assigned to meso-fauna, macro-arthropods and macro-invertebrates were assigned to macro-fauna. Any cases where authors did not classify the data to a taxonomic or size unit (e.g., described as ‘All soil fauna’), were classed into one category, “All Sizes”. The type of habitat the samples were from was also classified into one of six predefined classifications; agriculture, grassland, woody, cold/dry, wetlands, or artificial.

#### Effect sizes

Prior to calculating the effect size for each case, variances that were not already expressed as a standard deviation, were transformed. For cases where means were zero, variances were assumed to also be zero when missing. In two publications, non-zero (<0.1) standard deviations had been rounded to zero, these were set to 0.01 (in one publication the abundances were <0.1 individuals m^2^x10^3^, and in the other publication abundances were between 0 and 50 individuals). Cases that were missing non-zero variances in either the control or treatment were removed from the analysis, as standard deviations are needed to calculate the effect size used. As the majority of the data missing variances had been excluded during the earlier screening stages, imputation of missing variances (e.g., Kambach et al.^121^ Nakagawa et al;^122^) was deemed inappropriate.

For each case in the dataset, Hedges’ g,^120^ a standardized mean difference, was calculated using the metafor package.^123^ A standardized mean difference is required when different cases are on different scales,^101^ and this is a common effect size calculated in ecological meta-analyses.^101^ In addition, Hedges’ g is appropriate when zeros are present within either the control or treatment means (*n* = 127 zeros in control mean, and *n* = 159 zeros in treatment mean).^101^

Four effect sizes were removed from the database due to being extreme outliers of the dataset. The median effect size of the full dataset was −0.11 and the median variance was 0.42. Three of the removed data points had effect sizes smaller than −50 (ranging from −95 to −233), with variances >400 (ranging from 455 to 4538). The fourth datapoint that we removed had an effect size of 184, and variance of 568. Removal of the four data points had no impact on results. All four outliers were caused by relatively extreme changes in abundances and richness between the control and treatment.

#### Model structure

Multi-level random effects models were used for all models,^124^ using the metafor package.^123^ All models had a study-level ID (a unique ID to each primary literature source), with a unique case ID nested within it (each case, regardless of study or GC, was given a unique ID) as random effects. In addition, the community metric (abundance, biomass, taxonomic richness, Shannon diversity) was used as a crossed-effect within the random effects.^125^ Our dataset was heavily skewed toward effect sizes based on abundance data (71% of cases) preventing a robust model during *a priori* explorations when the community metric was used as a fixed effect interacting with other variables of interest. While the ideal modeling structure would have the community metric as a fixed effect, this current approach allows the variation associated with the community metric to still be accounted for. However, it was noted that although the community metric used in each case resulted in significantly different impacts on the effect size, all resulted in a negative impact (Figure S1).

For most of the models, the fixed effects were all structured similarly. For each model, the variable of interest (i.e., GC or the environmental stressor) was interacted with body size. The interaction was then tested with a Wald-Type test (‘anova’ function in metafor), and removed if *p* > 0.01. If the interaction was removed the singular effects were then tested, and removed if *p* > 0.01. Main effects were retained if the interaction was retained. The same process was repeated using methods analogous to the Knapp-Hartung method for model selection, namely using an F-distribution within the ‘anova’ function in the metafor package. However, model selection results did not change, thus are not presented. All models used a compound symmetry variance structure, and were fitted with restricted maximum-likelihood. Additionally, each effect size was weighted by the inverse of the variance-covariance matrix of the sampling errors (the default weighting in the metafor package).

Five broad groups of models were created. Firstly, a model was created with the six main GCs (hereafter, ‘main model’), where the six main GCs were the variable of interest, and the entire dataset was used. Secondly, using only the data for one GC at time (i.e., just data relating to climate change), a model was created where the variable of interest was the environmental stressor (Table 1; ‘environmental stressor models’). Not all data for each GC was used in the environmental stressor models, as some environmental stressors had too little to provide robust coefficients. Thirdly, to determine how responses vary across taxonomic groups, the dataset was subset to the four most represented taxonomic groups: Acari, Collembola, earthworms, and nematodes. These were not only well represented across the six GCs, but well distributed across all environmental stressors within the GCs. These four groups were then used as the variable of interest in one model (‘taxonomic model’); however, body size was not included in this model. To determine if the habitat type influenced the response, a model was created using the main six-level GC classification, habitat type, and the interaction between the two (‘habitat model’). Finally, a fifth model was created with soil pH (‘soil pH model’) as the variable of interest. Data on soil pH was only available for 1715 cases, and as Habitat Fragmentation only had 3 data points with pH it was not included as a GC within the model. Model simplification occurred as previously described.

#### Publication bias

Funnel plots were produced from the final main model and all the environmental stressor models, and therefore account for any covariates that were retained in the model. Funnel plots were produced using the ‘funnel’ function in the metafor package.^123^

To evaluate and address publication biases in the meta-analysis, we applied a two-step process based on methods in Nakagawa et al.^65^ The first step involves establishing whether publication bias, in terms of small-study effects and decline effects/time-lag bias, were present in the datasets. Small-study effects in meta-analyses are where studies with lower sample sizes have the largest effects,^126^ while a decline effect is when effect sizes vary with time, often reducing in magnitude over time.^101^ The second step involves correcting the model estimates for the biases that have been found. The two-step process was undertaken based on the main model, as well as each of the environmental stressor models.

For the first step, multi-level random effects models were created for the main model and the environmental stressor models with the same random-effects structure as above. The response variable was the standardized effect sizes, with moderators of the standard error of each effect size (i.e., the square root of the sampling variance of the effect size) and the year the original data were published (publication year, as a centered variable, i.e., centered on the mean year), both testing for biases. Other moderators included the GC, or the environmental stressor, depending on the model, and body size if it was a significant variable in the original model. No interactions were included among the moderators. A significant slope in the standard error predictor variable would indicate presence of small-study effects. A significant slope for the publication year variable, would indicate decline effects/time-lag bias.

Whenever one of the publication bias predictors (standard error or publication year) was significant, we applied the second step of the process and fitted a multi-level random effects model to adjust model estimates to account for these biases. As in the first step, a multi-level random effects model was created with the same random effects and response variable. Predictor variable would include the sampling variance of the effect size, publication year (centered on the mean year), GC or environmental stressor (depending on the dataset being tested), and body size if it was a significant variable in the original model. The coefficients from this model could then be compared with the original model, to ascertain whether results remain robust when accounting for biases.

All analyses were performed in R.^127^ The dataset used in the analysis is available: https://doi.org/10.5281/zenodo.6903152. The R code for data preparation and analysis, as well as the protocol for paper screening and data extraction can also be accessed: https://github.com/helenphillips/GCimpactsSB.

## Supplemental information

Document S1. Figures S1–S5, Tables S1 and S3, Data S1 and S2

Table S2. Taxonomic classifications Names of all taxonomic groups present in the dataset as given in the primary article (original taxonomic group), and the harmonized names from the Global Soil Biodiversity Atlas. Each harmonized name was then assigned to a body size category.
