# Supplementary material for: Global changes and their environmental stressors have a significant impact on soil biodiversity—A meta-analysis
Source: iScience. 2024 Jul 20;27(9):110540. doi: 10.1016/j.isci.2024.110540 (PMC11387903; doi:10.1016/j.isci.2024.110540)
Supplement: Document S1. Figures S1–S5, Tables S1 and S3, Data S1 and S2 [file mmc1.pdf]

iScience, Volume *27*

Supplemental information

Global changes and their environmental stressors have a significant impact on soil biodiversity—A meta-analysis

Helen R.P. Phillips, Erin K. Cameron, Nico Eisenhauer, Victoria J. Burton, Olga Ferlian, Yiming Jin, Sahana Kanabar, Sandhya Malladi, Rowan E. Murphy, Anne Peter, Isis Petrocelli, Christian Ristok, Katharine Tyndall, Wim van der Putten, and Léa Beaumelle

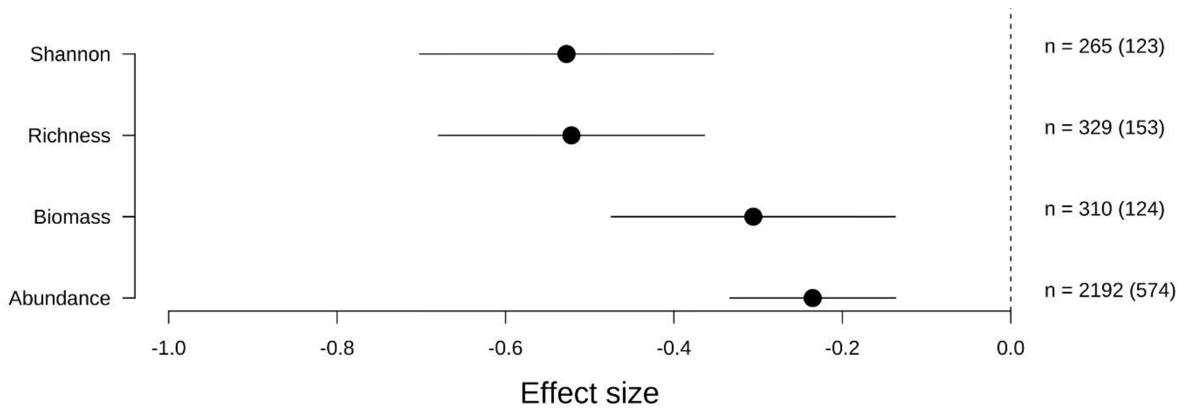

Figure S1: Impact of the community metric on effect size. Hedges' g was used as the effect size. Negative effect sizes indicate that the biodiversity measured using each metric has a negative impact from global change. A positive effect size indicates an increase in biodiversity for each measurement. Error bars indicate 95% confidence intervals. Effect sizes where error bars do not cross the dashed vertical zero line, are significantly from zero. The values of n indicate the number of cases of within each measurement type in the model, with values in parentheses indicating the number of publications.

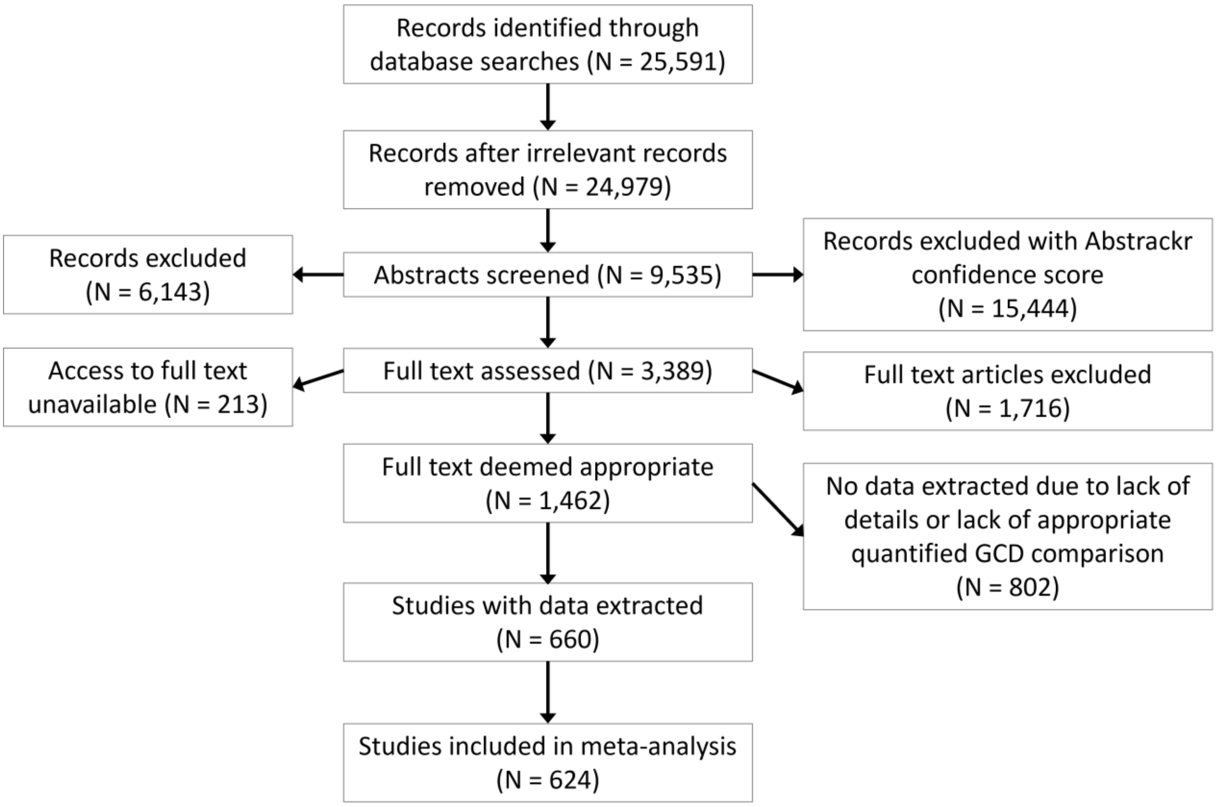

Figure S2: PRISMA diagram representing the workflow of the literature search.

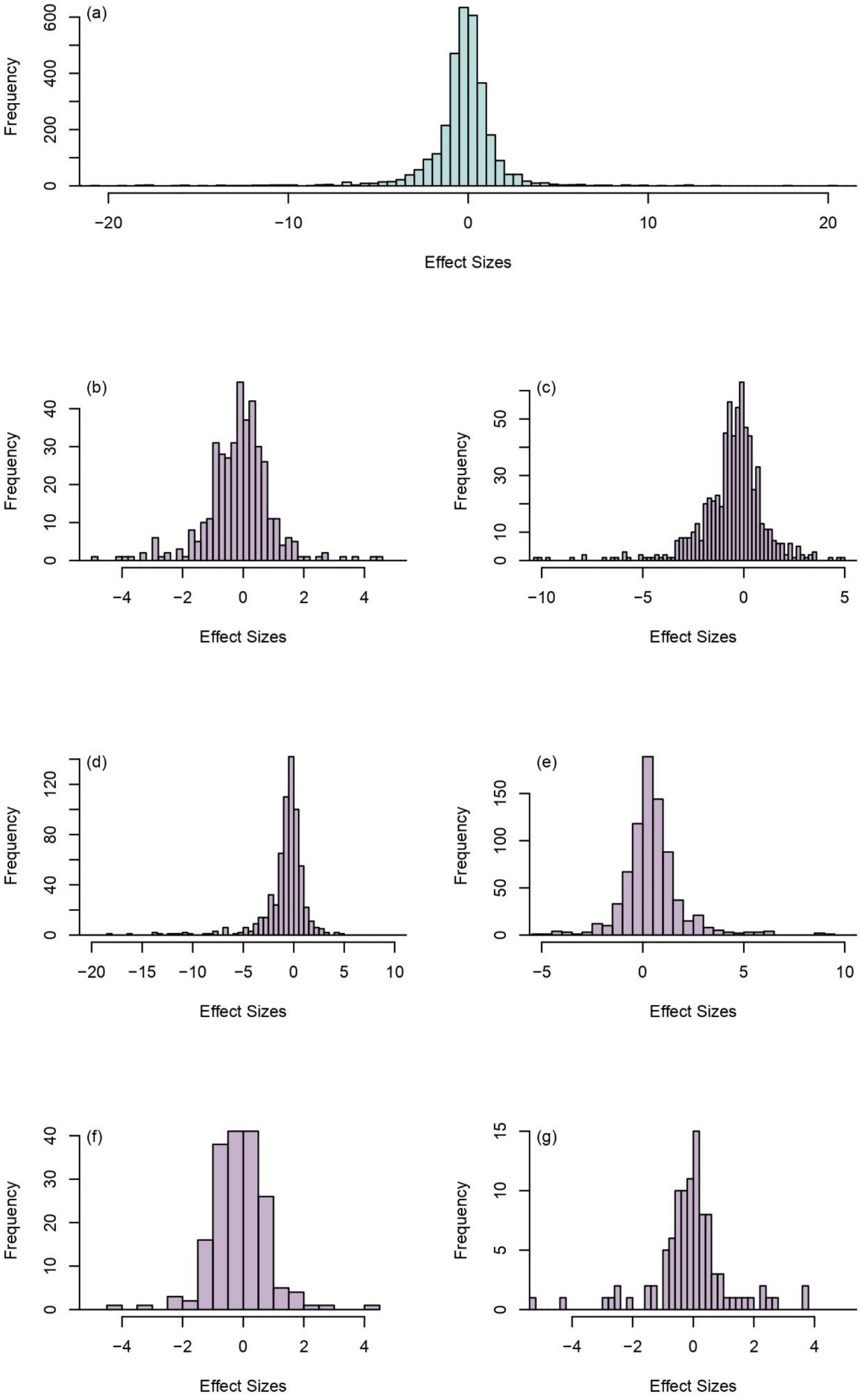

(a)
Frequency
0
200
400
600
−20
−10
0
10
20
Effect Sizes
(b)
Frequency
0
10
20
30
40
−4
−2
0
2
4
Effect Sizes
(c)
Frequency
0
10
30
50
−10
−5
0
5
Effect Sizes
(d)
Frequency
0
40
80
120
−20
−15
−10
−5
0
5
10
Effect Sizes
(e)
Frequency
0
50
100
150
−5
0
5
10
Effect Sizes
(f)
Frequency
0
10
20
30
40
−4
−2
0
2
4
Effect Sizes
(g)
Frequency
0
5
10
15
−4
−2
0
2
4
Effect Sizes

Figure S3: Distribution of the effect sizes for (a) the full model of GCDs, and the environmental stressors associated with (b) climate change, (c) land use intensification, (d) pollution, (e) nutrient enrichment, (f) invasive species and (g) habitat fragmentation. To aid readability, extreme outliers have been excluded from the figures.

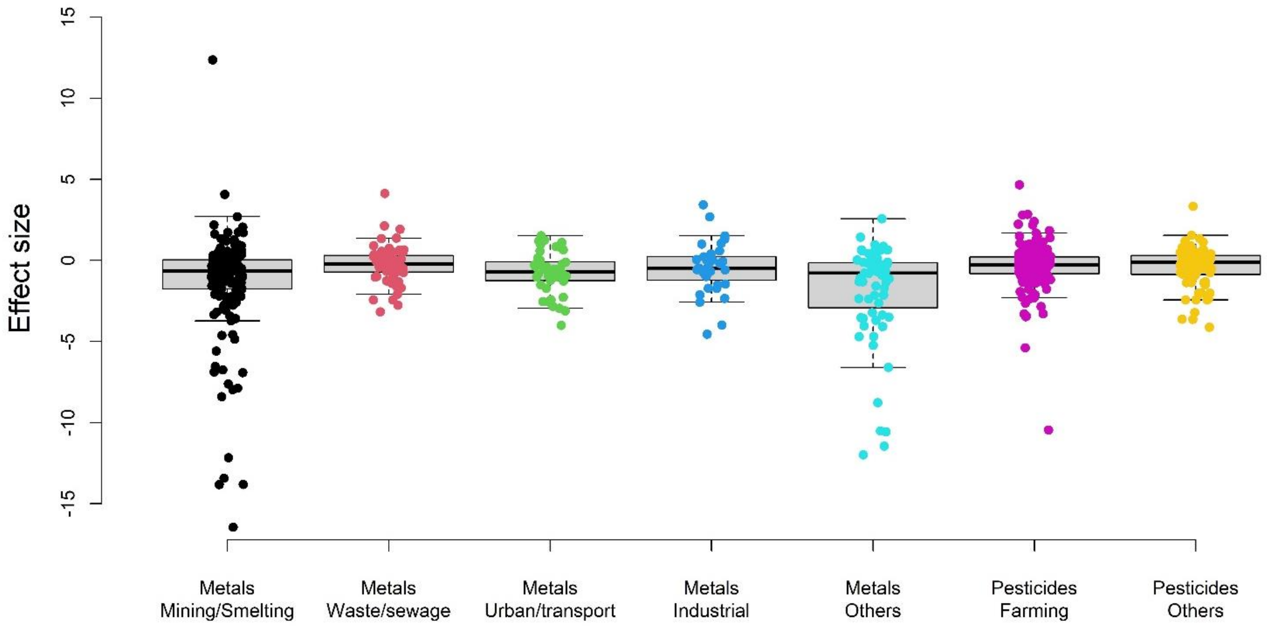

Figure S4: Raw effect sizes for the environmental stressors associated with pollution. Note, the y-axis has been truncated to -20 to 20, thus 8 effect sizes are not shown (three effect sizes < -20 from Metals - Mining/Smelting, Metals – Others and Pesticides – Others: five effect sizes > 20 from Metals - Mining/Smelting).

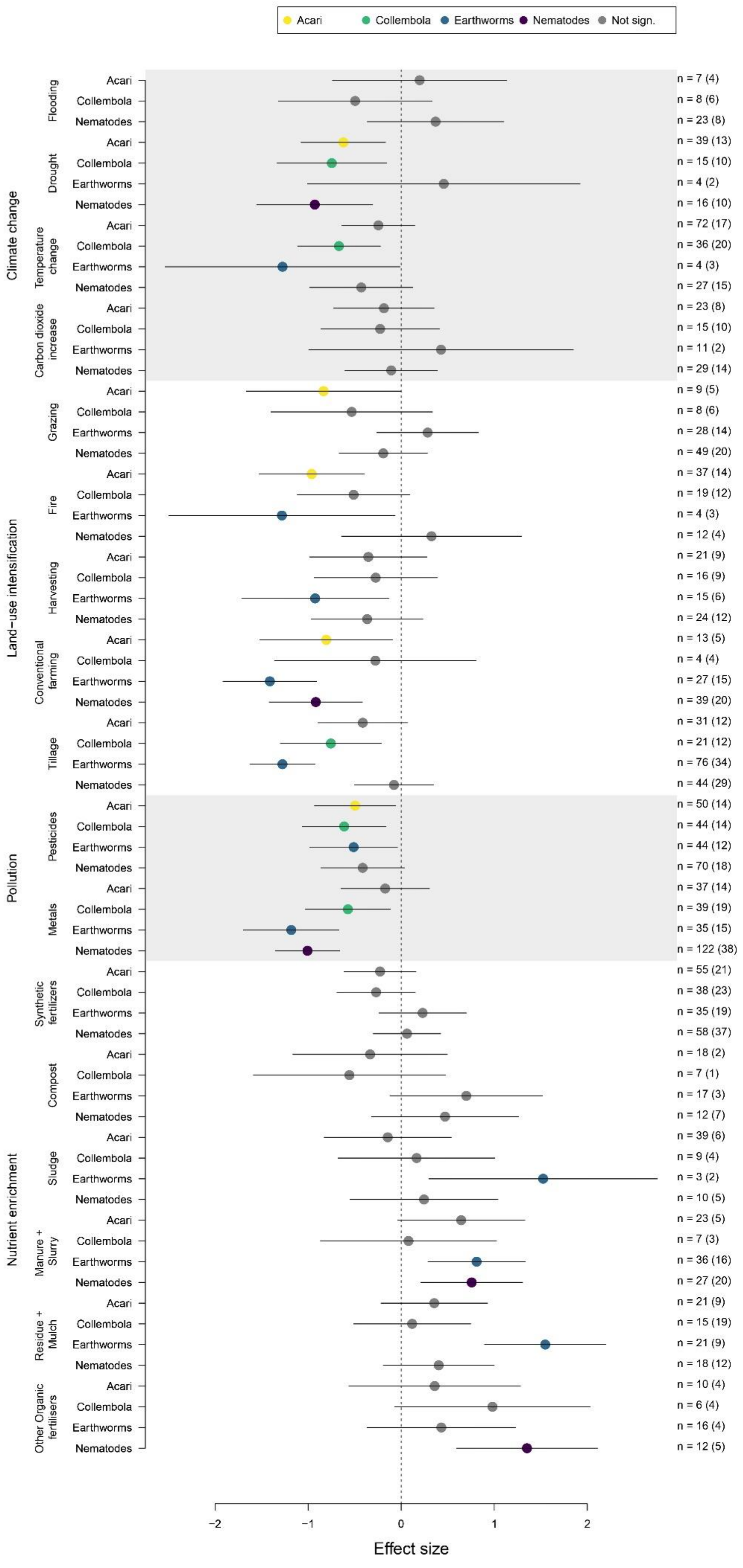

Acari
Collembola
Earthworms
Nematodes
Not sign.
Climate change
Flooding
Drought
Temperature change
Carbon dioxide increase
Land−use intensification
Grazing
Fire
Harvesting
Conventional farming
Tillage
Pollution
Pesticides
Metals
Nutrient enrichment
Synthetic fertilizers
Compost
Sludge
Manure + Slurry
Residue + Mulch
Other Organic fertilisers
n = 7 (4)
n = 8 (6)
n = 23 (8)
n = 39 (13)
n = 15 (10)
n = 4 (2)
n = 16 (10)
n = 72 (17)
n = 36 (20)
n = 4 (3)
n = 27 (15)
n = 23 (8)
n = 15 (10)
n = 11 (2)
n = 29 (14)
n = 9 (5)
n = 8 (6)
n = 28 (14)
n = 49 (20)
n = 37 (14)
n = 19 (12)
n = 4 (3)
n = 12 (4)
n = 21 (9)
n = 16 (9)
n = 15 (6)
n = 24 (12)
n = 13 (5)
n = 4 (4)
n = 27 (15)
n = 39 (20)
n = 31 (12)
n = 21 (12)
n = 76 (34)
n = 44 (29)
n = 50 (14)
n = 44 (14)
n = 44 (12)
n = 70 (18)
n = 37 (14)
n = 39 (19)
n = 35 (15)
n = 122 (38)
n = 55 (21)
n = 38 (23)
n = 35 (19)
n = 58 (37)
n = 18 (2)
n = 7 (1)
n = 17 (3)
n = 12 (7)
n = 39 (6)
n = 9 (4)
n = 3 (2)
n = 10 (5)
n = 23 (5)
n = 7 (3)
n = 36 (16)
n = 27 (20)
n = 21 (9)
n = 15 (19)
n = 21 (9)
n = 18 (12)
n = 10 (4)
n = 6 (4)
n = 16 (4)
n = 12 (5)
−2
−1
0
1
2
Effect size

Figure S5: Change in the biodiversity of four soil taxa groups (Acari, Collembola, earthworms, and nematodes) in response to the stressors of four global changes (GCs). Hedges' g was used as the effect size. Negative effect sizes indicate that the stressor causes a reduction in biodiversity, and a positive effect size indicates an increase in biodiversity. Error bars indicate 95% confidence intervals. Effect sizes where error bars do not cross the dashed vertical zero line, are significantly different from zero. Effect sizes which are not significantly different from zero are dark grey, other colours indicate the different taxa groups. The values of n indicate the number of cases of each taxa group within each stressor category in the model, with values in parentheses indicating the number of publications. Grey shading in background is for enhancing readability only. Note, there was no data representing earthworms impacted by Flooding, so no effect size is shown.

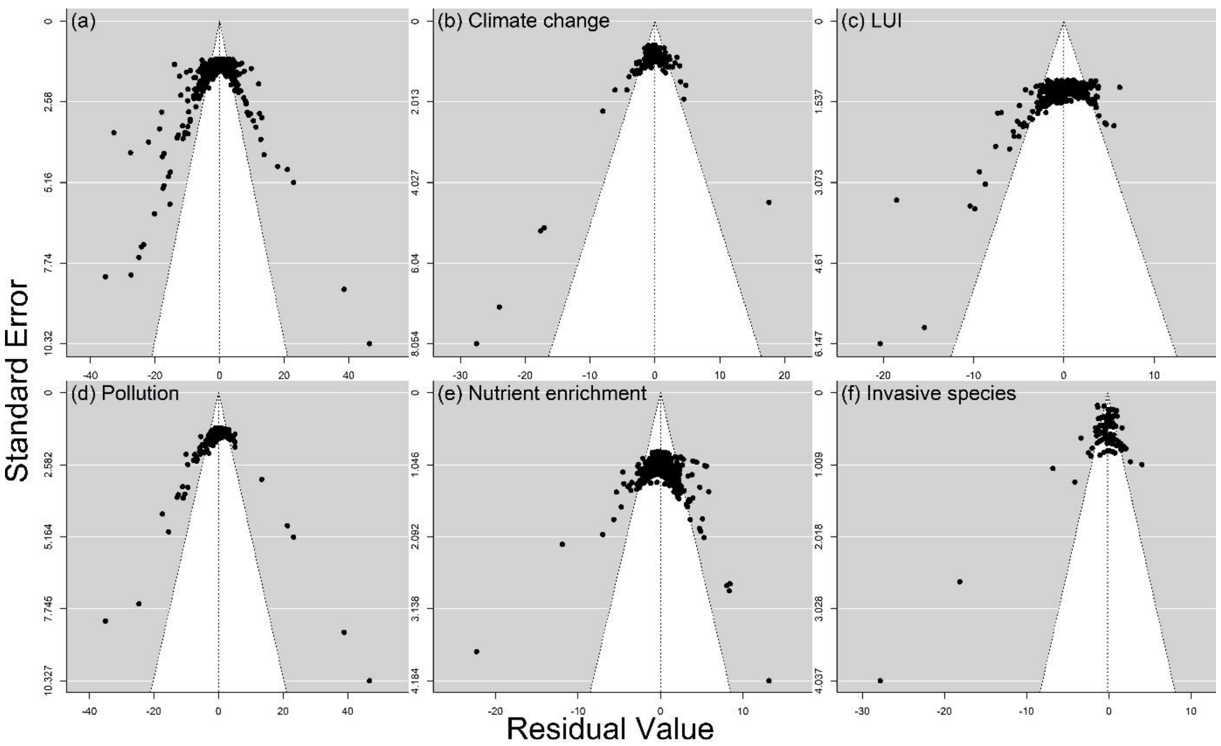

Figure S5: Funnel plots of data used in (a) the full model of GCDs, and the environmental stressors associated with (b) climate change, (c) land use intensification, (d) pollution, (e) nutrient enrichment and (f) invasive species. A funnel plot that is symmetrical would indicate no publication bias.

Table S1: Full list of GCDs and their associated stressors. Not all stressors had enough data to be included in the environmental stressor models, stressors in italics were excluded from the environmental stressor models, but not the main GCD model. Examples column provides some details of the type of data included in each stressor, but is not an exhaustive list.

**Global Change**	**Environmental stressor**	**Examples**
Climate change	CO_2 increase	Increase in amount
	Temperature change	Increase in amount, duration. Changes in number of freeze/thaw cycles
	Water availability – Drought	Decrease in water amount, rate, duration
	Water availability – Flood	Increase in water amount, rate, duration
	O_3 increase	Increase in amount
	Precipitation and Temperature change	Both changes in precipitation and temperature
	UVB Radiation	Increase in amount
Land use intensification	Grazing	Vertebrate and invertebrates, foliage removal (as proxy)
	Organic versus conventional farming	Combination of intensive agricultural practices (e.g. tillage, synthetic fertilizers) compared to more extensive practices used in organic farming (e.g. no tillage, organic fertilizers)
	Harvesting	Logging, selective harvesting, thinning, clear cut
	Fire	Controlled burns, wildfires, changes in intensity, amount, duration
	Tillage	Reduced, shallow depth, conventional
	Degradation	General disturbance and degradation (non-descript)
	Human population	Rural to urban comparisons
	Management	Weeding, planting rate, planting mechanism, landscape practices
	Irrigation	Specific water management (unrelated to climate change)
	Mono- versus poly-culture	Mixed agriculture (plantations, crops) compared to monoculture
Pollution	Metals	Lead (Pb), Cadmium (Cd), Zinc (Zn)
	Pesticides	Insecticides (e.g. neonicotinoids), Herbicides (e.g. glyphosate), Fungicides (e.g. carbendazim), fumigants
	Antibiotics	Bactericides, formaldehyde
	Endocrine disruptors	Bisphenol A, phtalates
	Radionuclides	Uranium, radiation
	Nanoparticles	Carbon nanomaterials
	PAHs	Accidental fuel or oil spills
	Pharmaceuticals	Ivermectin
	Salinization	Salt (chloride, sodium)

	Sulphate	Acid sulphate pollution
Nutrient enrichment	Synthetic fertilizers	N, P, K, NPK, compound fertilizers
	Ca-liming + Wood ash	Calcium carbonate, Ca(OH)2
	Compost	Vermicompost, farm compost, garden waste
	Manure + Slurry	Farmyard manure, animal manure, green manure, cattle slurry
	Mixture	Two or more different types of fertilizer
	Other Organic fertilisers	Sugars, bone meal, soybean byproducts
	Residue + Mulch	Straw, pine, clover residue, bark mulch, rice straw mulch
	Sludge (including Biosolids)	Sewage sludge, dried sludge
	Biochar	Wood biochar
Invasive species	Aboveground animal	Vertebrates, invertebrates
	Belowground animal	Invertebrates
	Plants - non-woody	*Microstegium* spp, *Spartina* spp, *Heracleum* spp
	Plants – woody	*Ailanthus* spp, *Robinia* spp
	Plants – mixture	Two or more invasive plants at a site
Habitat fragmentation	*Edge effects*	Distance from edge
	Fragmentation per se	Number of patches (constant area)
	Habitat amount	Decrease in area
	Isolation	Distance to source
	Corridors/Connectivity	Fragments connected or not

Table S3: Estimates, confidence intervals, z-values and p-values from the model showing the impacts of GCs on four taxonomic groups; acari, collembola, earthworms and nematodes. ':' indicates the interaction between the taxonomic group and the GC.

Term	Coefficient	CIs	z-value	p-value
Climate	-0.32	[-0.69,0.04]	-1.75	0.08
LUI	-0.61	[-0.94,-0.27]	-3.55	0.0004
Pollution	-0.54	[-0.91,-0.17]	-2.89	0.004
Nutrient enrichment	-0.09	[-0.44,0.25]	-0.53	0.59
Invasive species	-0.32	[-1.10,0.45]	-0.82	0.41
Habitat fragmentation	-0.51	[-1.22,0.19]	-1.42	0.15
Collembola	-0.30	[-0.63,0.04]	-1.75	0.08
Earthworms	0.26	[-0.59,1.11]	0.61	0.54
Nematodes	0.02	[-0.40,0.44]	0.10	0.92
LUI:Collembola	0.37	[-0.11,0.85]	1.50	0.13
LUI:Earthworms	-0.76	[-1.68,0.16]	-1.62	0.11
LUI:Nematodes	0.26	[-0.29,0.80]	0.92	0.36
Pollution:Collembola	0.15	[-0.34,0.63]	0.60	0.55
Pollution:Earthworms	-0.49	[-1.43,0.44]	-1.03	0.30
Pollution:Nematodes	-0.31	[-0.88,0.26]	-1.07	0.29
Nutrient enrichment:Collembola	0.27	[-0.19,0.73]	1.15	0.25
Nutrient enrichment:Earthworms	0.60	[-0.34,1.54]	1.25	0.21
Nutrient enrichment:Nematodes	0.34	[-0.20,0.88]	1.22	0.22
Invasive species:Collembola	0.14	[-0.74,1.01]	0.31	0.76
Invasive species:Earthworms	0.64	[-0.67,1.96]	0.96	0.34
Invasive species:Nematodes	-0.26	[-1.30,0.78]	-0.49	0.62
Habitat fragmentation:Collembola	0.79	[-0.02,1.60]	1.91	0.06
Habitat fragmentation:Earthworms	-0.07	[-1.42,1.27]	-0.11	0.91
Habitat fragmentation:Nematodes	0.09	[-0.84,1.02]	0.19	0.85

Data S1: Search terms

TS=(“global change” OR “environmental change” OR disturbance* OR stress*)

TS=(“land-use” OR “landuse” OR “land use” OR “agricultural intensi*” OR forest* OR agricultur* OR grassland* OR pasture* OR meadow* OR agroforest* OR plantation* OR urban* OR farm* OR abandon* OR fallow* OR graz* OR arable OR till* OR ploug* OR habitat degrad*” OR “habitat destruct*” OR logg* OR deforest* OR ((“land use” OR “landuse” OR “land-use OR cropland OR agricultur*) AND (intensi* OR expansion)))

TS=(“habitat loss” OR “habitat fragment*” OR “edge effect*” OR fragment*)

TS = (“climat* change” OR drought OR temperature* OR warming OR heat* OR precipitation* OR rain* OR flood* OR irrigation OR moisture OR watering OR fire OR “carbon dioxide” OR CO2)

TS=(pollut* OR contamin* OR toxi* OR metal* OR asbestos OR radionuclide* OR radioactiv* OR pharmaceutic* OR “emerging contamin*” OR “synthetic organic chem*” OR “personal care product*” OR plastic* OR “polycyclic aromatic hydrocarbon*” OR pesticide* or herbicide* or fungicide* or molluscide* or nematicide* or insecticide* OR agrochemical* OR “oil spill” OR “brine spill” OR “petrol spill” OR mining OR smelting OR “industrial activit*” OR “waste disposal” OR wastewater OR sludge OR sewage)

TS=(“nitrogen deposition” OR “nutrient deposition” OR “atmospheric deposition” OR *eutroph* OR fertili* OR “nutrient* enrichment” OR “nutrient pollut*”)

TS=(invad* OR exotic OR alien OR invas* OR non$native OR peregrine OR introduc* OR non$indigenous)

TS=(“species richness” OR richness OR “number of species” OR “number of taxa” OR diversity OR biomass OR Shannon* OR evenness OR abundance OR density OR communit*)

AND

TS=(
(((soil OR below$ground OR below-ground) AND (biota OR fauna OR micro$fauna OR macro$fauna OR meso$fauna OR animal* OR arthropod* OR invert* OR “inverte* decomposer*” OR detritivore* OR macroarthropod* OR rotifer* OR mite* OR acari* OR protozoa* OR tardigrad* OR isopod* OR protist* OR micro-arthropod* OR microarthropod* OR ciliat* OR termit*)) OR nematod* OR oligochaet* OR annelid* OR collembol* OR springtail* OR earthworm* OR enchytrae* OR lumbricid* OR “soil biodiversity” OR “below$ground biodiversity” OR “soil divers*” OR “below$ground divers*”))

The search retrieved 25,591 records, that were further filtered to remove obviously irrelevant papers using the Web of Science categories (MARINE FRESHWATER BIOLOGY OR OCEANOGRAPHY OR LIMNOLOGY were excluded) and non-primary research articles (REVIEW OR MEETING ABSTRACT OR NOTE OR NEWS ITEM OR RETRACTED PUBLICATION OR BIBLIOGRAPHY OR DATABASE REVIEW).

Supplemental References

1. A'Bear, A. D., Boddy, L., & Jones, T. H. (2013). Bottom-up determination of soil collembola diversity and population dynamics in response to interactive climatic factors. *Oecologia*, *173*(3), 1083–1087. doi:10.1007/s00442-013-2662-3
2. Abail, Z., & Whalen, J. K. (2018). Corn residue inputs influence earthworm population dynamics in a no-till corn-soybean rotation. *Applied Soil Ecology: A Section of Agriculture, Ecosystems & Environment*, *127*, 120–128. doi:10.1016/j.apsoil.2018.03.013
3. Al-Assiuty, A.-N. I. M., Khalil, M. A., Ismail, A.-W. A., van Straalen, N. M., & Ageba, M. F. (2014). Effects of fungicides and biofungicides on population density and community structure of soil oribatid mites. *The Science of the Total Environment*, *466–467*, 412–420. doi:10.1016/j.scitotenv.2013.07.063
4. Amossé, J., Bart, S., Péry, A. R. R., & Pelosi, C. (2018). Short-term effects of two fungicides on enchytraeid and earthworm communities under field conditions. *Ecotoxicology*, *27*(3), 300–312. doi:10.1007/s10646-018-1895-7
5. Andersen, L., Kühn, B. F., Bertelsen, M., Bruus, M., Larsen, S. E., & Strandberg, M. (2013). Alternatives to herbicides in an apple orchard, effects on yield, earthworms and plant diversity. *Agriculture, Ecosystems & Environment*, *172*, 1–5. doi:10.1016/j.agee.2013.04.004
6. Andrés, P., Moore, J. C., Simpson, R. T., Selby, G., Cotrufo, F., Denef, K., … Wall, D. H. (2016). Soil food web stability in response to grazing in a semi-arid prairie: The importance of soil textural heterogeneity. *Soil Biology & Biochemistry*, *97*, 131–143. doi:10.1016/j.soilbio.2016.02.014
7. Andrés, P., Mateos, E., Tarrasón, D., Cabrera, C., & Figuerola, B. (2011). Effects of digested, composted, and thermally dried sewage sludge on soil microbiota and mesofauna. *Applied Soil Ecology: A Section of Agriculture, Ecosystems & Environment*, *48*(2), 236–242. doi:10.1016/j.apsoil.2011.03.001
8. Andrés, P., & Domene, X. (2005). Ecotoxicological and fertilizing effects of dewatered, composted and dry sewage sludge on soil mesofauna: a TME experiment. *Ecotoxicology*, *14*(5), 545–557. doi:10.1007/s10646-005-0006-8
9. Andrés, P. (1999). Ecological risks of the use of sewage sludge as fertilizer in soil restoration: effects on the soil microarthropod populations. *Land Degrad. Dev., 10*, 67-77. https://doi.org/10.1002/(SICI)1099-145X(199901/02)10:1<67::AID-LDR322>3.0.CO;2-H
10. Andresen, L. C., Konestabo, H. S., Maraldo, K., Holmstrup, M., Ambus, P., Beier, C., & Michelsen, A. (2011). Organic matter flow in the food web at a temperate heath under multifactorial climate change. *Rapid Communications in Mass Spectrometry: RCM*, *25*(11), 1485–1496. doi:10.1002/rcm.4907
11. Andriuzzi, W. S., Pulleman, M. M., Cluzeau, D., & Pérès, G. (2017). Comparison of two widely used sampling methods in assessing earthworm community responses to agricultural intensification. *Applied Soil Ecology: A Section of Agriculture, Ecosystems & Environment*, *119*, 145–151. doi:10.1016/j.apsoil.2017.06.011
12. Andriuzzi, W. S., Keith, A. M., Bardgett, R. D., & van der Wal, R. (2013). Soil nematode assemblage responds weakly to grazer exclusion on a nutrient-rich seabird island. *European Journal of Soil Biology*, *58*, 38–41. doi:10.1016/j.ejsobi.2013.05.009
13. Arai, M., Miura, T., Tsuzura, H., Minamiya, Y., & Kaneko, N. (2018). Two-year responses of earthworm abundance, soil aggregates, and soil carbon to no-tillage and fertilization. *Geoderma*, *332*, 135–141. doi:10.1016/j.geoderma.2017.10.021

14. Aslam, T. J., Benton, T. G., Nielsen, U. N., & Johnson, S. N. (2015). Impacts of eucalypt plantation management on soil faunal communities and nutrient bioavailability: trading function for dependence? *Biology and Fertility of Soils*, *51*(5), 637–644. doi:10.1007/s00374-015-1003-6
15. Aström, J., & Pärt, T. (2013). Negative and matrix-dependent effects of dispersal corridors in an experimental metacommunity. *Ecology*, *94*(1), 72–82. doi:10.1890/11-1795.1
16. Attignon, S. E., Lachat, T., Sinsin, B., Nagel, P., & Peveling, R. (2005). Termite assemblages in a West-African semi-deciduous forest and teak plantations. *Agriculture, Ecosystems & Environment*, *110*(3–4), 318–326. doi:10.1016/j.agee.2005.04.020
17. Atwood, L. W., Mortensen, D. A., Koide, R. T., & Smith, R. G. (2018). Evidence for multi-trophic effects of pesticide seed treatments on non-targeted soil fauna. *Soil Biology & Biochemistry*, *125*, 144–155. doi:10.1016/j.soilbio.2018.07.007
18. Auclerc, A., Nahmani, J., Aran, D., Baldy, V., Callot, H., Gers, C., … Guérold, F. (2012). Changes in soil macroinvertebrate communities following liming of acidified forested catchments in the Vosges Mountains (North-eastern France). *Ecological Engineering*, *42*, 260–269. doi:10.1016/j.ecoleng.2012.02.024
19. Ayuke, F. O., Brussaard, L., Vanlauwe, B., Six, J., Lelei, D. K., Kibunja, C. N., & Pulleman, M. M. (2011). Soil fertility management: Impacts on soil macrofauna, soil aggregation and soil organic matter allocation. *Applied Soil Ecology: A Section of Agriculture, Ecosystems & Environment*, *48*(1), 53–62. doi:10.1016/j.apsoil.2011.02.001
20. Azpilicueta, C. V., Aruani, M. C., Chaves, E., & Reeb, P. D. (2014). Soil nematode responses to fertilization with ammonium nitrate after six years of unfertilized apple orchard. *Revista de Investigacion Agraria [Spanish Journal of Agricultural Research]*, *12*(2), 353. doi:10.5424/sjar/2014122-4634
21. Badejo, M.A. (1994). Effect of accidental fire on soil mite density in a forest reserve in Nigeria. *Exp Appl Acarol 18*, 703–710. https://doi.org/10.1007/BF00051537
22. Bai, X., Zhao, S., & Duo, L. (2017). Impacts of carbon nanomaterials on the diversity of microarthropods in turfgrass soil. *Scientific Reports*, *7*(1), 1779. doi:10.1038/s41598-017-01920-z
23. Bai, P.-H., Liu, Q.-Z., Li, X.-Y., Liu, Y.-B., & Zhang, L.-L. (2018). Response of the wheat rhizosphere soil nematode community in wheat/walnut intercropping system in Xinjiang, Northwest China. *Applied Entomology and Zoology*, *53*(3), 297–306. doi:10.1007/s13355-018-0557-9
24. Bakonyi, G., Nagy, P., & Kádár, I. (2003). Long-term effects of heavy metals and microelements on nematode assemblage. *Toxicology Letters*, *140–141*, 391–401. doi:10.1016/s0378-4274(03)00035-3
25. Baldivieso-Freitas, P., Blanco-Moreno, J. M., Gutiérrez-López, M., Peigné, J., Pérez-Ferrer, A., Trigo-Aza, D., & Sans, F. X. (2018). Earthworm abundance response to conservation agriculture practices in organic arable farming under Mediterranean climate. *Pedobiologia*, *66*, 58–64. doi:10.1016/j.pedobi.2017.10.002
26. Bao, X., Li, Q., Hua, J., Zhao, T., & Liang, W. (2014). Interactive effects of elevated ozone and UV-B radiation on soil nematode diversity. *Ecotoxicology*, *23*(1), 11–20. doi:10.1007/s10646-013-1146-x
27. Barbercheck, M. E., Neher, D. A., Anas, O., El-Allaf, S. M., & Weicht, T. R. (2009). Response of soil invertebrates to disturbance across three resource regions in North Carolina. *Environmental Monitoring and Assessment*, *152*(1–4), 283–298. doi:10.1007/s10661-008-0315-5
28. Bardgett, R. D., Leemans, D. K., Cook, R., & Hobbs, P. J. (1997). Seasonality of the soil biota of grazed and ungrazed hill grasslands. *Soil Biology & Biochemistry*, *29*(8), 1285–1294. doi:10.1016/s0038-0717(97)00019-9
29. Barratt, B. I. P., Tozer, P. A., Wiedemer, R. L., Ferguson, C. M., & Johnstone, P. D. (2006). Effect of fire on microarthropods in New Zealand indigenous grassland. *Rangeland Ecology & Management*, *59*(4), 383–391. doi:10.2111/05-190r1.1

30. Barros, E., Neves, A., Blanchart, E., Fernandes, E. C. M., Wandelli, E., & Lavelle, P. (2003). Development of the soil macrofauna community under silvopastoral and agrosilvicultural systems in Amazonia. *Pedobiologia*, *47*(3), 273–280. doi:10.1078/0031-4056-00190
31. Battigelli, Jeffrey P., Spence, J. R., Langor, D. W., & Berch, S. M. (2004). Short-term impact of forest soil compaction and organic matter removal on soil mesofauna density and oribatid mite diversity. *Canadian Journal of Forest Research*, *34*(5), 1136–1149. doi:10.1139/x03-267
32. Battigelli, J. P., McIntyre, G. S., Broersma, K., & Krzic, M. (2003). Impact of cattle grazing on prostigmatid mite densities in grassland soils of southern interior British Columbia. *Canadian Journal of Soil Science*, *83*(5), 533–535. doi:10.4141/s02-064
33. Beck, L., Römbke, J., Ruf, A., Prinzing, A., & Woas, S. (2004). Effects of diflubenzuron and Bacillus thuringiensis var. kurstaki toxin on soil invertebrates of a mixed deciduous forest in the Upper Rhine Valley, Germany. *European Journal of Soil Biology*, *40*(1), 55–62. doi:10.1016/j.ejsobi.2003.08.003
34. Bedano, J. C., Domínguez, A., Arolfo, R., & Wall, L. G. (2016). Effect of Good Agricultural Practices under no-till on litter and soil invertebrates in areas with different soil types. *Soil & Tillage Research*, *158*, 100–109. doi:10.1016/j.still.2015.12.005
35. Belnap, J., Phillips, S. L., Sherrod, S. K., & Moldenke, A. (2005). Soil Biota can change after exotic plant invasion: Does this affect ecosystem processes? *Ecology*, *86*(11), 3007–3017. doi:10.1890/05-0333
36. Belnap, J. & Phillips, S.L. (2001). Soil biota in an ungrazed grassland: Response to annual grass (Bromus tectorum) invasion. *Ecological Applications, 11*: 1261-1275. https://doi.org/10.1890/1051-0761(2001)011[1261:SBIAUG]2.0.CO;2
37. Bengtsson, J., Persson, T., & Lundkvist, H. (1997). Long-term effects of logging residue addition and removal on macroarthropods and enchytraeids. *The Journal of Applied Ecology*, *34*(4), 1014. doi:10.2307/2405290
38. Bengtsson, J., Lundkvist, H., Saetre, P., Sohlenius, B., & Solbreck, B. (1998). Effects of organic matter removal on the soil food web: Forestry practices meet ecological theory. *Applied Soil Ecology: A Section of Agriculture, Ecosystems & Environment*, *9*(1–3), 137–143. doi:10.1016/s0929-1393(98)00067-5
39. Berch, Shannon M., Brockley, R. P., Battigelli, J. P., Hagerman, S., & Holl, B. (2006). Impacts of repeated fertilization on components of the soil biota under a young lodgepole pine stand in the interior of British Columbia. *Canadian Journal of Forest Research*, *36*(6), 1415–1426. doi:10.1139/x06-037
40. Berch, Shannon Marie, Battigelli, J. P., & Hope, G. D. (2007). Responses of soil mesofauna communities and oribatid mite species to site preparation treatments in high-elevation cutblocks in southern British Columbia. *Pedobiologia*, *51*(1), 23–32. doi:10.1016/j.pedobi.2006.12.001
41. Bert, V., Lors, C., Ponge, J.-F., Caron, L., Biaz, A., Dazy, M., & Masfaraud, J.-F. (2012). Metal immobilization and soil amendment efficiency at a contaminated sediment landfill site: a field study focusing on plants, springtails, and bacteria. *Environmental Pollution*, *169*, 1–11. doi:10.1016/j.envpol.2012.04.021
42. Beyer, S., Kinnear, A., Hutley, L. B., McGuinness, K., & Gibb, K. (2011). Assessing the relationship between fire and grazing on soil characteristics and mite communities in a semi-arid savanna of northern Australia. *Pedobiologia*, *54*(3), 195–200. doi:10.1016/j.pedobi.2011.03.002
43. Bezkorovaynaya, I. N. (2014). Forest-tundra soil invertebrate communities under conditions of technogenic pollution. *Contemporary Problems of Ecology*, *7*(6), 708–713. doi:10.1134/s199542551406002x

44. Binet, F., Hallaire, V., & Curmi, P. (1997). Agricultural practices and the spatial distribution of earthworms in maize fields. Relationships between earthworm abundance, maize plants and soil compaction. *Soil Biology & Biochemistry*, *29*(3–4), 577–583. doi:10.1016/s0038-0717(96)00182-4
45. Bird, S. B., Coulson, R. N., & Fisher, R. F. (2004). Changes in soil and litter arthropod abundance following tree harvesting and site preparation in a loblolly pine (Pinus taeda L.) plantation. *Forest Ecology and Management*, *202*(1–3), 195–208. doi:10.1016/j.foreco.2004.07.023
46. Bishop, H. O., Grieve, I. C., Chudek, J. A., & Hopkins, D. W. (2008). Liming upland grassland: the effects on earthworm communities and the chemical characteristics of carbon in casts. *European Journal of Soil Science*, *59*(3), 526–531. doi:10.1111/j.1365-2389.2007.01009.x
47. Blanchart, E., & Julka, J.M. (1997). Influence of forest disturbance on earthworm (Oligochaeta) communities in the Western Ghats (South India). *Soil Biology and Biochemistry, 29 (3-4),* 303-306. doi: 10.1016/S0038-0717(96)00094-6.
48. Blanchart, E., Villenave, C., Viallatoux, A., Barthès, B., Girardin, C., Azontonde, A., & Feller, C. (2006). Long-term effect of a legume cover crop (Mucuna pruriens var. utilis) on the communities of soil macrofauna and nematofauna, under maize cultivation, in southern Benin. *European Journal of Soil Biology*, *42*, S136–S144. doi:10.1016/j.ejsobi.2006.07.018
49. Blanchet, G., Gavazov, K., Bragazza, L., & Sinaj, S. (2016). Responses of soil properties and crop yields to different inorganic and organic amendments in a Swiss conventional farming system. *Agriculture, Ecosystems & Environment, 230*, 116-126. doi:10.1016/j.agee.2016.05.032.
50. Bloemers, G. F., Hodda, M., Lambshead, P. J. D., Lawton, J. H., & Wanless, F. R. (1997). The effects of forest disturbance on diversity of tropical soil nematodes. *Oecologia*, *111*(4), 575–582. doi:10.1007/s004420050274
51. Bohlen, P. J., Edwards, W. M., & Edwards, C. A. (1995). Earthworm community structure and diversity in experimental agricultural watersheds in Northeastern Ohio. *Plant and Soil*, *170*(1), 233–239. doi:10.1007/bf02183069
52. Bokhorst, S., Huiskes, A., Convey, P., van Bodegom, P. M., & Aerts, R. (2008). Climate change effects on soil arthropod communities from the Falkland Islands and the Maritime Antarctic. *Soil Biology & Biochemistry*, *40*(7), 1547–1556. doi:10.1016/j.soilbio.2008.01.017
53. Bokhorst, Stef, (Ciska) Veen, G. F., Sundqvist, M., De Long, J. R., Kardol, P., & Wardle, D. A. (2018). Contrasting responses of springtails and mites to elevation and vegetation type in the sub-Arctic. *Pedobiologia*, *67*, 57–64. doi:10.1016/j.pedobi.2018.02.004
54. Bokhorst, S., Phoenix, G. K., Bjerke, J. W., Callaghan, T. V., Huyer-Brugman, F., & Berg, M. P. (2012). Extreme winter warming events more negatively impact small rather than large soil fauna: shift in community composition explained by traits not taxa. *Global Change Biology*, *18*(3), 1152–1162. doi:10.1111/j.1365-2486.2011.02565.x
55. Bokhorst, Stef, Phoenix, G. K., Berg, M. P., Callaghan, T. V., Kirby-Lambert, C., & Bjerke, J. W. (2015). Climatic and biotic extreme events moderate long-term responses of above- and belowground sub-Arctic heathland communities to climate change. *Global Change Biology*, *21*(11), 4063–4075. doi:10.1111/gcb.13007
56. Borgström, P., Bommarco, R., Strengbom, J., & Viketoft, M. (2018). Above- and belowground insect herbivores mediate the impact of nitrogen eutrophication on the soil food web in a grassland ecosystem. *Oikos (Copenhagen, Denmark)*, *127*(9), 1272–1279. doi:10.1111/oik.04763
57. Boutsis, G., Stamou, G., & Argyropoulou, M. (2011). Short term effects of soil disinfection with metham sodium and organic alternatives on nematode communities. *Community Ecology: CE*, *12*(2), 161–170. doi:10.1556/comec.12.2011.2.3

58. Bouwman, L. A., Bloem, J., Römkens, P. F. A. M., & Japenga, J. (2005). EDGA amendment of slightly heavy metal loaded soil affects heavy metal solubility, crop growth and microbivorous nematodes but not bacteria and herbivorous nematodes. *Soil Biology & Biochemistry*, *37*(2), 271–278. doi:10.1016/j.soilbio.2004.07.039
59. Bouwman, L. A., Bloem, J., Römkens, P. F. A., Boon, G. T., & Vangronsveld J. (2001). Beneficial effects of the growth of metal tolerant grass on biological and chemical parameters in copper- and zinc contaminated sandy soils. *Minerva Biotecnologica 13*(1), 19-26.
60. Brennan, A., Fortune, T., & Bolger, T. (2006). Collembola abundances and assemblage structures in conventionally tilled and conservation tillage arable systems. *Pedobiologia*, *50*(2), 135–145. doi:10.1016/j.pedobi.2005.09.004
61. Brévault, T., Bikay, S., Maldès, J. M., & Naudin, K. (2007). Impact of a no-till with mulch soil management strategy on soil macrofauna communities in a cotton cropping system. *Soil & Tillage Research*, *97*(2), 140–149. doi:10.1016/j.still.2007.09.006
62. Briar, S. S., Grewal, P. S., Somasekhar, N., Stinner, D., & Miller, S. A. (2007). Soil nematode community, organic matter, microbial biomass and nitrogen dynamics in field plots transitioning from conventional to organic management. *Applied Soil Ecology: A Section of Agriculture, Ecosystems & Environment*, *37*(3), 256–266. doi:10.1016/j.apsoil.2007.08.004
63. Briar, S. S., Fonte, S. J., Park, I., Six, J., Scow, K., & Ferris, H. (2011). The distribution of nematodes and soil microbial communities across soil aggregate fractions and farm management systems. *Soil Biology & Biochemistry*, *43*(5), 905–914. doi:10.1016/j.soilbio.2010.12.017
64. Briar, S. S., Culman, S. W., Young-Mathews, A., Jackson, L. E., & Ferris, H. (2012). Nematode community responses to a moisture gradient and grazing along a restored riparian corridor. *European Journal of Soil Biology*, *50*, 32–38. doi:10.1016/j.ejsobi.2011.11.006
65. Briones, M. J. I., Ostle, N. J., McNamara, N. P., & Poskitt, J. (2009). Functional shifts of grassland soil communities in response to soil warming. *Soil Biology & Biochemistry*, *41*(2), 315–322. doi:10.1016/j.soilbio.2008.11.003
66. Brown, G. G., Benito, N. P., Pasini, A., Sautter, K. D., de F Guimarães, M., & Torres, E. (2003). No-tillage greatly increases earthworm populations in Paraná state, Brazil. *Pedobiologia*, *47*(5–6), 764–771. doi:10.1078/0031-4056-00256
67. Broza, M., & Izhaki, I. (1997). Post-fire arthropod assemblages in Mediterranean forest soils in Israel. *International Journal of Wildland Fire*, *7*(4), 317. doi:10.1071/wf9970317
68. Bueno, C. G., & Jiménez, J. J. (2014). Livestock grazing activities and wild boar rooting affect alpine earthworm communities in the Central Pyrenees (Spain). *Applied Soil Ecology: A Section of Agriculture, Ecosystems & Environment*, *83*, 71–78. doi:10.1016/j.apsoil.2014.04.013
69. Burtis, J. C., Fahey, T. J., & Yavitt, J. B. (2014). Impact of invasive earthworms on Ixodes scapularis and other litter-dwelling arthropods in hardwood forests, central New York state, USA. *Applied Soil Ecology: A Section of Agriculture, Ecosystems & Environment*, *84*, 148–157. doi:10.1016/j.apsoil.2014.07.005
70. Butenko, K. O., Gongalsky, K. B., Korobushkin, D. I., Ekschmitt, K., & Zaitsev, A. S. (2017). Forest fires alter the trophic structure of soil nematode communities. *Soil Biology & Biochemistry*, *109*, 107–117. doi:10.1016/j.soilbio.2017.02.006
71. Byrne, L. B., Bruns, M. A., & Kim, K. C. (2008). Ecosystem properties of urban land covers at the aboveground–belowground interface. *Ecosystems (New York, N.Y.)*, *11*(7), 1065–1077. doi:10.1007/s10021-008-9179-3

72. Callaham, M. (2003). Macroinvertebrates in North American tallgrass prairie soils: effects of fire, mowing, and fertilization on density and biomass. *Soil Biology & Biochemistry*, *35*(8), 1079–1093. doi:10.1016/s0038-0717(03)00153-6
73. Camann, M., Lamoncha, K., & Gillette, N. (2012). Oribatid mite community decline two years after low-intensity burning in the southern cascade range of California, USA. *Forests*, *3*(4), 959–985. doi:10.3390/f3040959
74. Camann, M. A., Gillette, N. E., Lamoncha, K. L., & Mori, S. R. (2008). Response of forest soil Acari to prescribed fire following stand structure manipulation in the southern Cascade Range. *Canadian Journal of Forest Research*, *38*(5), 956–968. doi:10.1139/x07-241
75. Cameron, E. K., Knysh, K. M., Proctor, H. C., & Bayne, E. M. (2013). Influence of two exotic earthworm species with different foraging strategies on abundance and composition of boreal microarthropods. *Soil Biology & Biochemistry*, *57*, 334–340. doi:10.1016/j.soilbio.2012.07.010
76. Cao, Z., Han, X., Hu, C., Chen, J., Zhang, D., & Steinberger, Y. (2011). Changes in the abundance and structure of a soil mite (Acari) community under long-term organic and chemical fertilizer treatments. *Applied Soil Ecology: A Section of Agriculture, Ecosystems & Environment*, *49*, 131–138. doi:10.1016/j.apsoil.2011.06.003
77. Cardoso, M. S. O., Pedrosa, E. M. R., Rolim, M. M., Oliveira, L. S. C., & Santos, A. N. (2016). Relationship between nematode assemblages and physical properties across land use types. *Tropical Plant Pathology*, *41*(2), 107–114. doi:10.1007/s40858-016-0075-7
78. Carrascosa, Marina, Sánchez-Moreno, S., & Alonso-Prados, J. L. (2015). Effects of organic and conventional pesticides on plant biomass, nematode diversity and the structure of the soil food web. *Nematology: International Journal of Fundamental and Applied Nematological Research*, *17*(1), 11–26. doi:10.1163/15685411-00002849
79. Carrascosa, M., Sánchez-Moreno, S., & Alonso-Prados, J. L. (2014). Relationships between nematode diversity, plant biomass, nutrient cycling and soil suppressiveness in fumigated soils. *European Journal of Soil Biology*, *62*, 49–59. doi:10.1016/j.ejsobi.2014.02.009
80. Carter, M. R., & Noronha, C. (2007). Soil micro-arthropod communities and microbial parameters in the potato ridge under two field management systems on sandy loams in Atlantic Canada. *Canadian Journal of Soil Science*, *87*(4), 399–404. doi:10.4141/cjss06013
81. Castellanos-Navarrete, A., Rodríguez-Aragonés, C., de Goede, R. G. M., Kooistra, M. J., Sayre, K. D., Brussaard, L., & Pulleman, M. M. (2012). Earthworm activity and soil structural changes under conservation agriculture in central Mexico. *Soil & Tillage Research*, *123*, 61–70. doi:10.1016/j.still.2012.03.011
82. Castro, J., Lago, M. C. F., Briones, M. J. I., Gallego, P. P. & Barreal, M. E. (2015). Effects of agricultural practices on soil fauna communities in kiwifruit plantations. *Acta Hortic.* 1096, 267-273. doi: 10.17660/ActaHortic.2015.1096.29
83. Čerevková, A., & Renčo, M. (2009). Soil nematode community changes associated with windfall and wildfire in forest soil at the High Tatras National Park, Slovak Republic. *Helminthologia*, *46*(2), 123–130. doi:10.2478/s11687-009-0024-9
84. Čerevková, A., Miklisová, D., & Cagáň, Ľ. (2017). Effects of experimental insecticide applications and season on soil nematode communities in a maize field. *Crop Protection (Guildford, Surrey)*, *92*, 1–15. doi:10.1016/j.cropro.2016.10.007
85. Čerevková, A., & Cagáň, Ľ. (2013). The influence of Western corn rootworm seed coating and granular insecticides on the seasonal fluctuations of soil nematode communities in a maize field. *Helminthologia*, *50*(3), 205–214. doi:10.2478/s11687-013-0132-4

86. Cesarz, S., Ciobanu, M., Wright, A. J., Ebeling, A., Vogel, A., Weisser, W. W., & Eisenhauer, N. (2017). Plant species richness sustains higher trophic levels of soil nematode communities after consecutive environmental perturbations. *Oecologia*, *184*(3), 715–728. doi:10.1007/s00442-017-3893-5
87. Chang, L., Wu, H., Wu, D., & Sun, X. (2013). Effect of tillage and farming management on Collembola in marsh soils. *Applied Soil Ecology: A Section of Agriculture, Ecosystems & Environment*, *64*, 112–117. doi:10.1016/j.apsoil.2012.11.007
88. Chauvat, M., & Ponge, J.-F. (2002). Colonization of heavy metal-polluted soils by collembola: preliminary experiments in compartmented boxes. *Applied Soil Ecology: A Section of Agriculture, Ecosystems & Environment*, *21*(2), 91–106. doi:10.1016/s0929-1393(02)00087-2
89. Chelinho, S., Domene, X., Andrés, P., Natal-da-Luz, T., Norte, C., Rufino, C., … Sousa, J. P. (2014). Soil microarthropod community testing: A new approach to increase the ecological relevance of effect data for pesticide risk assessment. *Applied Soil Ecology: A Section of Agriculture, Ecosystems & Environment*, *83*, 200–209. doi:10.1016/j.apsoil.2013.06.009
90. Chen, X., Daniell, T. J., Neilson, R., O'Flaherty, V., & Griffiths, B. S. (2014). Microbial and microfaunal communities in phosphorus limited, grazed grassland change composition but maintain homeostatic nutrient stoichiometry. *Soil Biology & Biochemistry*, *75*, 94–101. doi:10.1016/j.soilbio.2014.03.024
91. Chen, H., Li, B., Fang, C., Chen, J., & Wu, J. (2007). Exotic plant influences soil nematode communities through litter input. *Soil Biology & Biochemistry*, *39*(7), 1782–1793. doi:10.1016/j.soilbio.2007.02.011
92. Chen, G., Qin, J., Shi, D., Zhang, Y., & Ji, W. (2009). Diversity of soil nematodes in areas polluted with heavy metals and polycyclic aromatic hydrocarbons (PAHs) in Lanzhou, China. *Environmental Management*, *44*(1), 163–172. doi:10.1007/s00267-008-9268-2
93. Chen, D., Lan, Z., Hu, S., & Bai, Y. (2015). Effects of nitrogen enrichment on belowground communities in grassland: Relative role of soil nitrogen availability vs. soil acidification. *Soil Biology & Biochemistry*, *89*, 99–108. doi:10.1016/j.soilbio.2015.06.028
94. Chen, D., Lan, Z., Bai, X., Grace, J. B., & Bai, Y. (2013). Evidence that acidification-induced declines in plant diversity and productivity are mediated by changes in below-ground communities and soil properties in a semi-arid steppe. *The Journal of Ecology*, *101*(5), 1322–1334. doi:10.1111/1365-2745.12119
95. Choudhary, M., Jat, H. S., Datta, A., Yadav, A. K., Sapkota, T. B., Mondal, S., … Jat, M. L. (2018). Sustainable intensification influences soil quality, biota, and productivity in cereal-based agroecosystems. *Applied Soil Ecology: A Section of Agriculture, Ecosystems & Environment*, *126*, 189–198. doi:10.1016/j.apsoil.2018.02.027
96. Choudhary, M., Datta, A., Jat, H. S., Yadav, A. K., Gathala, M. K., Sapkota, T. B., … Ladha, J. K. (2018). Changes in soil biology under conservation agriculture based sustainable intensification of cereal systems in Indo-Gangetic Plains. *Geoderma*, *313*, 193–204. doi:10.1016/j.geoderma.2017.10.041
97. Christopher, C. C., & Cameron, G. N. (2012). Effects of Invasive Amur Honeysuckle (Lonicera maackii) and White-Tailed Deer (Odocoileus virginianus) on Litter-Dwelling Arthropod Communities. *The American Midland Naturalist*, *167*(2), 256–272. doi:10.1674/0003-0031-167.2.256
98. Clapperton, M. J., Kanashiro, D. A., & Behan-Pelletier, V. M. (2002). Changes in abundance and diversity of microarthropods associated with Fescue Prairie grazing regimes. *Pedobiologia*, *46*(5), 496–511. doi:10.1078/0031-4056-00155
99. Cole, L., Buckland, S. M., & Bardgett, R. D. (2008). Influence of disturbance and nitrogen addition on plant and soil animal diversity in grassland. *Soil Biology & Biochemistry*, *40*(2), 505–514. doi:10.1016/j.soilbio.2007.09.018

100. Cole, Lisa, Buckland, S. M., & Bardgett, R. D. (2005). Relating microarthropod community structure and diversity to soil fertility manipulations in temperate grassland. *Soil Biology & Biochemistry*, *37*(9), 1707–1717. doi:10.1016/j.soilbio.2005.02.005
101. Coll, P., Coll, P., Le Cadre, E., Coll, P., Le Cadre, E., & Villenave, C. (2012). How are nematode communities affected during a conversion from conventional to organic farming in southern French vineyards? *Nematology: International Journal of Fundamental and Applied Nematological Research*, *14*(6), 665–676. doi:10.1163/156854112x624195
102. Coll, P., Le Cadre, E., Blanchart, E., Hinsinger, P., & Villenave, C. (2011). Organic viticulture and soil quality: A long-term study in Southern France. *Applied Soil Ecology: A Section of Agriculture, Ecosystems & Environment*. doi:10.1016/j.apsoil.2011.07.013
103. Convey, P., & Wynn-Williams, D. D. (2002). Antarctic soil nematode response to artificial climate amelioration. *European Journal of Soil Biology*, *38*(3–4), 255–259. doi:10.1016/s1164-5563(02)01155-x
104. Convey, P., Abbandonato, H., Bergan, F., Beumer, L. T., Biersma, E. M., Bråthen, V. S., … Coulson, S. J. (2015). Survival of rapidly fluctuating natural low winter temperatures by High Arctic soil invertebrates. *Journal of Thermal Biology*, *54*, 111–117. doi:10.1016/j.jtherbio.2014.07.009
105. Coors, A., Edwards, M., Lorenz, P., Römbke, J., Schmelz, R. M., Topp, E., … Lapen, D. R. (2016). Biosolids applied to agricultural land: Influence on structural and functional endpoints of soil fauna on a short- and long-term scale. *The Science of the Total Environment*, *562*, 312–326. doi:10.1016/j.scitotenv.2016.03.226
106. Cornwell, E. (2014). Effects of different agricultural systems on soil quality in Northern Limón province, Costa Rica. *Revista de Biología Tropical, 62*(3), 887-897.
107. Cortet, J., Ronce, D., Poinsot-Balaguer, N., Beaufreton, C., Chabert, A., Viaux, P., & Paulo Cancela de Fonseca, J. (2002). Impacts of different agricultural practices on the biodiversity of microarthropod communities in arable crop systems. *European Journal of Soil Biology*, *38*(3–4), 239–244. doi:10.1016/s1164-5563(02)01152-4
108. Cotrufo, M. F., Soong, J., Vandegehuchte, M. L., Nguyen, T., Denef, K., Ashley Shaw, E., … Wall, D. H. (2014). Naphthalene addition to soil surfaces: A feasible method to reduce soil micro-arthropods with negligible direct effects on soil C dynamics. *Applied Soil Ecology: A Section of Agriculture, Ecosystems & Environment*, *74*, 21–29. doi:10.1016/j.apsoil.2013.09.008
109. Coulson, S. J., Leinaas, H. P., Ims, R. A., & Sovik, G. (2000). Experimental manipulation of the winter surface ice layer: the effects on a High Arctic soil microarthropod community. *Ecography*, *23*(3), 299–306. doi:10.1034/j.1600-0587.2000.d01-1638.x
110. Coyle, D. R., Zalesny, J. A., Zalesny, R. S., Jr, & Wiese, A. H. (2011). Irrigating poplar energy crops with landfill leachate negatively affects soil micro- and meso-fauna. *International Journal of Phytoremediation*, *13*(9), 845–858. doi:10.1080/15226514.2011.552927
111. Creamer, R. E., Rimmer, D. L., & Black, H. I. J. (2008). Do elevated soil concentrations of metals affect the diversity and activity of soil invertebrates in the long-term? *Soil Use and Management*, *24*(1), 37–46. doi:10.1111/j.1475-2743.2007.00131.x
112. Crittenden, S. J., Huerta, E., de Goede, R. G. M., & Pulleman, M. M. (2015). Earthworm assemblages as affected by field margin strips and tillage intensity: An on-farm approach. *European Journal of Soil Biology*, *66*, 49–56. doi:10.1016/j.ejsobi.2014.11.007
113. Čuchta, P., Miklisová, D., & Kováč, L. (2013). The impact of disturbance and ensuing forestry practices on Collembola in monitored stands of windthrown forest in the Tatra National Park (Slovakia). *Environmental Monitoring and Assessment*, *185*(6), 5085–5098. doi:10.1007/s10661-012-2927-z

114. Culik, M. P., de Souza, J. L., & Ventura, J. A. (2002). Biodiversity of Collembola in tropical agricultural environments of Espírito Santo, Brazil. *Applied Soil Ecology: A Section of Agriculture, Ecosystems & Environment*, *21*(1), 49–58. doi:10.1016/s0929-1393(02)00048-3
115. Curry, J. P., Doherty, P., Purvis, G., & Schmidt, O. (2008). Relationships between earthworm populations and management intensity in cattle-grazed pastures in Ireland. *Applied Soil Ecology: A Section of Agriculture, Ecosystems & Environment*, *39*(1), 58–64. doi:10.1016/j.apsoil.2007.11.005
116. D'Annibale, A., Sechi, V., Larsen, T., Christensen, S., Krogh, P. H., & Eriksen, J. (2017). Does introduction of clover in an agricultural grassland affect the food base and functional diversity of Collembola? *Soil Biology & Biochemistry*, *112*, 165–176. doi:10.1016/j.soilbio.2017.05.010
117. D'Hose, T., Cougnon, M., De Vliegher, A., Vandecasteele, B., Viaene, N., Cornelis, W., … Reheul, D. (2014). The positive relationship between soil quality and crop production: A case study on the effect of farm compost application. *Applied Soil Ecology: A Section of Agriculture, Ecosystems & Environment*, *75*, 189–198. doi:10.1016/j.apsoil.2013.11.013
118. Dam, M., Vestergård, M., & Christensen, S. (2012). Freezing eliminates efficient colonizers from nematode communities in frost-free temperate soils. *Soil Biology & Biochemistry*, *48*, 167–174. doi:10.1016/j.soilbio.2012.01.017
119. Dam, M., & Christensen, S. (2015). Defoliation reduces soil biota - and modifies stimulating effects of elevated CO2. *Ecology and Evolution*, *5*(21), 4840–4848. doi:10.1002/ece3.1739
120. Dam, M., Bergmark, L., & Vestergård, M. (2017). Elevated CO2 increases fungal-based micro-foodwebs in soils of contrasting plant species. *Plant and Soil*, *415*(1–2), 549–561. doi:10.1007/s11104-017-3191-3
121. da Silva Moço, M. K., da Gama-Rodrigues, E. F., da Gama-Rodrigues, A. C., Machado, R. C. R., & Baligar, V. C. (2009). Soil and litter fauna of cacao agroforestry systems in Bahia, Brazil. *Agroforestry Systems*, *76*(1), 127–138. doi:10.1007/s10457-008-9178-6
122. Dávalos, A., Simpson, E., Nuzzo, V., & Blossey, B. (2015). Non-consumptive effects of native deer on introduced earthworm abundance. *Ecosystems (New York, N.Y.)*, *18*(6), 1029–1042. doi:10.1007/s10021-015-9881-x
123. Davidson, A. W., & Potter, D. A. (1995). Response of plant-feeding, predatory, and soil-inhabiting invertebrates to Acremonium endophyte and nitrogen fertilization in tall fescue turf. *Journal of Economic Entomology*, *88*(2), 367–379. doi:10.1093/jee/88.2.367
124. Dawes-Gromadzki, T. Z. (2007). Short-term effects of low intensity fire on soil macroinvertebrate assemblages in different vegetation patch types in an Australian tropical savanna. *Austral Ecology*, *32*(6), 663–668. doi:10.1111/j.1442-9993.2007.01752.x
125. de Aquino, A. M., Ferreira da Silva, R., Mercante, F. M., Fernandes Correia, M. E., de Fátima Guimarães, M., & Lavelle, P. (2008). Invertebrate soil macrofauna under different ground cover plants in the no-till system in the Cerrado. *European Journal of Soil Biology*, *44*(2), 191–197. doi:10.1016/j.ejsobi.2007.05.001
126. de Goede, R. G. M. (1996). Effects of sod-cutting on the nematode community of a secondary forest of Pinus sylvestris L. *Biology and Fertility of Soils*, *22*(3), 227–236. doi:10.1007/s003740050103
127. De Long, J. R., Laudon, H., Blume-Werry, G., & Kardol, P. (2016). Nematode community resistant to deep soil frost in boreal forest soils. *Pedobiologia*, *59*(5–6), 243–251. doi:10.1016/j.pedobi.2016.10.001
128. De Long, J. R., Dorrepaal, E., Kardol, P., Nilsson, M.-C., Teuber, L. M., & Wardle, D. A. (2016). Contrasting responses of soil microbial and nematode communities to warming and plant functional group removal across a post-fire boreal forest successional gradient. *Ecosystems*, *19*(2), 339–355. doi:10.1007/s10021-015-9935-0

129. de Rouw, A., Huon, S., Soulileuth, B., Jouquet, P., Pierret, A., Ribolzi, O., … Chantharath, B. (2010). Possibilities of carbon and nitrogen sequestration under conventional tillage and no-till cover crop farming (Mekong valley, Laos). *Agriculture, Ecosystems & Environment*, *136*(1–2), 148–161. doi:10.1016/j.agee.2009.12.013

130. De Silva, P. M. C. S., Pathiratne, A., van Straalen, N. M., & van Gestel, C. A. M. (2010). Chlorpyrifos causes decreased organic matter decomposition by suppressing earthworm and termite communities in tropical soil. *Environmental Pollution*, *158*(10), 3041–3047. doi:10.1016/j.envpol.2010.06.032

131. De Smedt, P., Wuyts, K., Baeten, L., De Schrijver, A., Proesmans, W., De Frenne, P., … Verheyen, K. (2016). Complementary distribution patterns of arthropod detritivores (woodlice and millipedes) along forest edge-to-interior gradients. *Insect Conservation and Diversity*, *9*(5), 456–469. doi:10.1111/icad.12183

132. Debeljak, M., Cortet, J., Demšar, D., Krogh, P. H., & Džeroski, S. (2007). Hierarchical classification of environmental factors and agricultural practices affecting soil fauna under cropping systems using Bt maize. *Pedobiologia*, *51*(3), 229–238. doi:10.1016/j.pedobi.2007.04.009

133. Déchêne, A. D., & Buddle, C. M. (2009). Effects of experimental forest harvesting on oribatid mite biodiversity. *Forest Ecology and Management*, *258*(7), 1331–1341. doi:10.1016/j.foreco.2009.06.033

134. Degens, B. P., Schipper, L. A., Claydon, J. J., Russell, J. M., & Yeates, G. W. (2000). Irrigation of an allophanic soil with dairy factory effluent for 22 years: responses of nutrient storage and soil biota. *Soil Research*, *38*(1), 25. doi:10.1071/sr99063

135. Delgado, J. D., Arroyo, N. L., Arévalo, J. R., & Fernández-Palacios, J. M. (2013). Road edge effects on litter invertebrate communities of subtropical forests. *Journal of Natural History*, *47*(3–4), 203–236. doi:10.1080/00222933.2012.743610

136. Deru, J. G. C., Bloem, J., de Goede, R., Keidel, H., Kloen, H., Rutgers, M., … van Eekeren, N. (2018). Soil ecology and ecosystem services of dairy and semi-natural grasslands on peat. *Applied Soil Ecology: A Section of Agriculture, Ecosystems & Environment*, *125*, 26–34. doi:10.1016/j.apsoil.2017.12.011

137. Desgarennes, D., Carrion, G., & López-Lima, J. D. (2018). Integrated management reduces Globodera rostochiensis abundance and enhances nematode community composition. *Archiv Fur Acker- Und Pflanzenbau Und Bodenkunde*, *64*(1), 1–12. doi:10.1080/03650340.2017.1322195

138. Dey, A. K., & Chaudhuri, P. (2014). Earthworm community structure of pineapple (Ananas comosus) plantations under monoculture and mixed culture in West Tripura, India. *Tropical Ecology 55*(1). doi: 10.5455/ijmsph.2014.260920141

139. Dighton, J., Helmisaari, H.-S., Maghirang, M., Smith, S., Malcolm, K., Johnson, W., … Kukkola, M. (2012). Impacts of forest post thinning residues on soil chemistry, fauna and roots: Implications of residue removal in Finland. *Applied Soil Ecology: A Section of Agriculture, Ecosystems & Environment*, *60*, 16–22. doi:10.1016/j.apsoil.2012.02.023

140. Dmowska E. (2007). Nematode communities along the transect shelterbelt - ecotone - crop field. *Polish Journal of Ecology, 55*(4), 665-680.

141. Doamba, S. W. M. F., Savadogo, P., & Nacro, H. B. (2014). Effects of burning on soil macrofauna in a savanna-woodland under different experimental fuel load treatments. *Applied Soil Ecology: A Section of Agriculture, Ecosystems & Environment*, *81*, 37–44. doi:10.1016/j.apsoil.2014.04.005

142. Domínguez, A., Bedano, J. C., Becker, A. R., & Arolfo, R. V. (2014). Organic farming fosters agroecosystem functioning in Argentinian temperate soils: Evidence from litter decomposition and soil

fauna. *Applied Soil Ecology: A Section of Agriculture, Ecosystems & Environment*, *83*, 170–176. doi:10.1016/j.apsoil.2013.11.008

143. Domínguez, A., & Bedano, J. C. (2016). The adoption of no-till instead of reduced tillage does not improve some soil quality parameters in Argentinean Pampas. *Applied Soil Ecology: A Section of Agriculture, Ecosystems & Environment*, *98*, 166–176. doi:10.1016/j.apsoil.2015.10.014

144. Dong, Z., Hou, R., Chen, Q., Ouyang, Z., & Ge, F. (2013). Response of soil nematodes to elevated temperature in conventional and no-tillage cropland systems. *Plant and Soil*, *373*(1–2), 907–918. doi:10.1007/s11104-013-1846-2

145. Dorel, M., Lakhia, S., Pététin, C., Bouamer, S., & Risède, J.-M. (2010). No-till banana planting on crop residue mulch: effect on soil quality and crop functioning. *Fruits*, *65*(2), 55–68. doi:10.1051/fruits/20010001

146. Dosso, K., Konaté, S., Aidara, D., & Linsenmair, K. E. (2010). Termite diversity and abundance across fire-induced habitat variability in a tropical moist savanna (Lamto, Central Côte d'Ivoire). *Journal of Tropical Ecology*, *26*(3), 323–334. doi:10.1017/s0266467410000015

147. Dress, W. J., & Boerner, R. E. J. (2004). Patterns of microarthropod abundance in oak–hickory forest ecosystems in relation to prescribed fire and landscape position. *Pedobiologia*, *48*(1), 1–8. doi:10.1016/j.pedobi.2003.03.001

148. Dubie, T. R., Greenwood, C. M., Godsey, C., & Payton, M. E. (2011). Effects of tillage on soil microarthropods in winter wheat. *The Southwestern Entomologist*, *36*(1), 11–20. doi:10.3958/059.036.0102

149. Duncan, K., Jennings, E., Buck, P., Wells, H., Kolhatkar, R., Sublette, K., … Todd, T. (2003). Multi-species ecotoxicity assessment of petroleum-contaminated soil. *Soil & Sediment Contamination*, *12*(2), 181–206. doi:10.1080/713610969

150. Duo, L., He, L., & Zhao, S. (2017). The impact of modified nanoscale carbon black on soil nematode assemblages under turfgrass growth conditions. *European Journal of Soil Biology*, *80*, 53–58. doi:10.1016/j.ejsobi.2017.04.004

151. Dutoit, T., Decaens, T., & Alard, D. (1997). Successional changes and diversity of soil macrofaunal communities on chalk grasslands in Upper-Normandy (France). *Acta Oecologica*, *18*(2), 135–149. doi:10.1016/s1146-609x(97)80070-7

152. Eisenhauer, N., Cesarz, S., Koller, R., Worm, K., & Reich, P. B. (2012). Global change belowground: impacts of elevated CO2, nitrogen, and summer drought on soil food webs and biodiversity. *Global Change Biology*, *18*(2), 435–447. doi:10.1111/j.1365-2486.2011.02555.x

153. Emmerling, C. (2001). Response of earthworm communities to different types of soil tillage. *Applied Soil Ecology: A Section of Agriculture, Ecosystems & Environment*, *17*(1), 91–96. doi:10.1016/s0929-1393(00)00132-3

154. Eo, J., Park, K.-C., & Park, B.-B. (2012). Short-term effects of organic waste amendments on soil biota: responses of soil food web under eggplant cultivation. *Soil Research*, *50*(5), 436. doi:10.1071/sr12013

155. Eriksen-Hamel, N. S., Speratti, A. B., Whalen, J. K., Légère, A., & Madramootoo, C. A. (2009). Earthworm populations and growth rates related to long-term crop residue and tillage management. *Soil & Tillage Research*, *104*(2), 311–316. doi:10.1016/j.still.2009.04.006

156. Ernst, G., & Emmerling, C. (2009). Impact of five different tillage systems on soil organic carbon content and the density, biomass, and community composition of earthworms after a ten year period. *European Journal of Soil Biology*, *45*(3), 247–251. doi:10.1016/j.ejsobi.2009.02.002

157. Errouissi, F., Ben Moussa-Machraoui, S., Ben-Hammouda, M., & Nouira, S. (2011). Soil invertebrates in durum wheat (Triticum durum L.) cropping system under Mediterranean semi arid

conditions: A comparison between conventional and no-tillage management. *Soil & Tillage Research*, *112*(2), 122–132. doi:10.1016/j.still.2010.12.004
158. Estevez, B., N'Dayegamiye, A., & Coderre, D. (1996). The effect on earthworm abundance and selected soil properties after 14 years of solid cattle manure and NPKMg fertilizer application. *Canadian Journal of Soil Science*, *76*(3), 351–355. doi:10.4141/cjss96-042
159. Faber, F., Wachter, E., & Zaller, J. G. (2017). Earthworms are little affected by reduced soil tillage methods in vineyards. *Plant, Soil and Environment*, *63*(6), 257–263. doi:10.17221/160/2017-pse
160. Falco, L. B., Sandler, R., Momo, F., Di Ciocco, C., Saravia, L., & Coviella, C. (2015). Earthworm assemblages in different intensity of agricultural uses and their relation to edaphic variables. *PeerJ*, *3*, e979. doi:10.7717/peerj.979
161. Farská, J., Prejzková, K., & Rusek, J. (2013). Spruce monoculture establishment affects functional traits of soil microarthropod communities. *Biologia*, *68*(3), 479–486. doi:10.2478/s11756-013-0168-4
162. Farská, J., Prejzková, K., & Rusek, J. (2014). Management intensity affects traits of soil microarthropod community in montane spruce forest. *Applied Soil Ecology: A Section of Agriculture, Ecosystems & Environment*, *75*, 71–79. doi:10.1016/j.apsoil.2013.11.003
163. Favretto, M. R., Paoletti, M. G., Caporali, F., Nannipieri, P., Onnis, A., & Tomei, P. E. (1992). Invertebrates and nutrients in a Mediterranean vineyard mulched with subterranean clover (Trifolium subterraneum L.). *Biology and Fertility of Soils*, *14*(3), 151–158. doi:10.1007/bf00346055
164. Felten, D., & Emmerling, C. (2011). Effects of bioenergy crop cultivation on earthworm communities—A comparative study of perennial (Miscanthus) and annual crops with consideration of graded land-use intensity. *Applied Soil Ecology: A Section of Agriculture, Ecosystems & Environment*, *49*, 167–177. doi:10.1016/j.apsoil.2011.06.001
165. Fenoglio, S., Cucco, M., Bo, T., Gay, P. & Malacarne, G. (2007). Colonisation by ground and edaphic invertebrates of soil patches with different pollution levels. *Periodicum Biologorum, 109*(1), 37-45.
166. Ferris, H., Venette, R. C., & Lau, S. S. (1996). Dynamics of nematode communities in tomatoes grown in conventional and organic farming systems, and their impact on soil fertility. *Applied Soil Ecology: A Section of Agriculture, Ecosystems & Environment*, *3*(2), 161–175. doi:10.1016/0929-1393(95)00071-2
167. Fiera, C. (2009). Biodiversity of Collembola in urban soils and their use as bioindicators for pollution. *Pesquisa Agropecuaria Brasileira*, *44*(8), 868–873. doi:10.1590/s0100-204x2009000800010
168. Fisk, M. C., Kessler, W. R., Goodale, A., Fahey, T. J., Groffman, P. M., & Driscoll, C. T. (2006). Landscape variation in microarthropod response to calcium addition in a northern hardwood forest ecosystem. *Pedobiologia*, *50*(1), 69–78. doi:10.1016/j.pedobi.2005.11.001
169. Fonte, S. J., Winsome, T., & Six, J. (2009). Earthworm populations in relation to soil organic matter dynamics and management in California tomato cropping systems. *Applied Soil Ecology: A Section of Agriculture, Ecosystems & Environment*, *41*(2), 206–214. doi:10.1016/j.apsoil.2008.10.010
170. Fonte, S. J., Barrios, E., & Six, J. (2010). Earthworms, soil fertility and aggregate-associated soil organic matter dynamics in the Quesungual agroforestry system. *Geoderma*, *155*(3–4), 320–328. doi:10.1016/j.geoderma.2009.12.016
171. Förster, B., Garcia, M., Francimari, O., & Römbke, J. (2006). Effects of carbendazim and lambda-cyhalothrin on soil invertebrates and leaf litter decomposition in semi-field and field tests under tropical conditions (Amazônia, Brazil). *European Journal of Soil Biology*, *42*, S171–S179. doi:10.1016/j.ejsobi.2006.07.011

172. Fountain, M., Hopkin, S. (2004). A Comparative Study of the Effects of Metal Contamination on Collembola in the Field and in the Laboratory. *Ecotoxicology 13*, 573–587. https://doi.org/10.1023/B:ECTX.0000037194.66321.2c
173. Fountain, M., Hopkin, S. (2004). Biodiversity of Collembola in Urban Soils and the Use of Folsomia candida to Assess Soil 'Quality'. *Ecotoxicology 13*, 555–572. https://doi.org/10.1023/B:ECTX.0000037192.70167.00
174. Frainer, A., & Duarte, M. M. (2009). Soil invertebrates in southern Brazilian Araucaria forest - grassland mosaic: differences between disturbed and undisturbed areas. *Iheringia. Serie Zoologia*, *99*(3), 307–312. doi:10.1590/s0073-47212009000300013
175. Francini, G., Liiri, M., Männistö, M., Stark, S., & Kytöviita, M.-M. (2014). Response to reindeer grazing removal depends on soil characteristics in low Arctic meadows. *Applied Soil Ecology: A Section of Agriculture, Ecosystems & Environment*, *76*, 14–25. doi:10.1016/j.apsoil.2013.12.003
176. Fraser, P. M., Schon, N. L., Piercy, J. E., Mackay, A. D., & Minor, M. A. (2012). Influence of summer irrigation on soil invertebrate populations in a long-term sheep irrigation trial at Winchmore (Canterbury). *New Zealand Journal of Agricultural Research*, *55*(2), 165–180. doi:10.1080/00288233.2012.662902
177. Frazão, J., de Goede, R. G. M., Brussaard, L., Faber, J. H., Groot, J. C. J., & Pulleman, M. M. (2017). Earthworm communities in arable fields and restored field margins, as related to management practices and surrounding landscape diversity. *Agriculture, Ecosystems & Environment*, *248*, 1–8. doi:10.1016/j.agee.2017.07.014
178. Freckman, D. W., & Ettema, C. H. (1993). Assessing nematode communities in agroecosystems of varying human intervention. *Agriculture, Ecosystems & Environment*, *45*(3–4), 239–261. doi:10.1016/0167-8809(93)90074-y
179. Frimpong, J. O., Ofori, E. S. K., Yeboah, S., Marri, D., Offei, B. K., Apaatah, F., … Osae, M. (2018). Evaluating the impact of synthetic herbicides on soil dwelling macrobes and the physical state of soil in an agro-ecosystem. *Ecotoxicology and Environmental Safety*, *156*, 205–215. doi:10.1016/j.ecoenv.2018.03.034
180. Frøseth, R. B., Bakken, A. K., Bleken, M. A., Riley, H., Pommeresche, R., Thorup-Kristensen, K., & Hansen, S. (2014). Effects of green manure herbage management and its digestate from biogas production on barley yield, N recovery, soil structure and earthworm populations. *European Journal of Agronomy: The Journal of the European Society for Agronomy*, *52*, 90–102. doi:10.1016/j.eja.2013.10.006
181. Frouz, J., Hrčková, K., Lána, J., Krištůfek, V., Mudrák, O., Lukešová, A., & Mihaljevič, M. (2011). Can laboratory toxicity tests explain the pattern of field communities of algae, plants, and invertebrates along a toxicity gradient of post-mining sites? *Applied Soil Ecology: A Section of Agriculture, Ecosystems & Environment*, *51*, 114–121. doi:10.1016/j.apsoil.2011.09.007
182. Fu, S., Coleman, D. C., Hendrix, P. F., & Crossley, D. A., Jr. (2000). Responses of trophic groups of soil nematodes to residue application under conventional tillage and no-till regimes. *Soil Biology & Biochemistry*, *32*(11–12), 1731–1741. doi:10.1016/s0038-0717(00)00091-2
183. Fusilero, M. A., Mangubat, J., Ragas, R. E., Baguinon, N., Taya, H., & Rasco, E., Jr. (2013). Weed management systems and other factors affecting the earthworm population in a banana plantation. *European Journal of Soil Biology*, *56*, 89–94. doi:10.1016/j.ejsobi.2013.03.002
184. Gan, Huije, Zak, D. R., & Hunter, M. D. (2013). Chronic nitrogen deposition alters the structure and function of detrital food webs in a northern hardwood ecosystem. *Ecological Applications: A Publication of the Ecological Society of America*, *23*(6), 1311–1321. doi:10.1890/12-1895.1

185. Gan, Huijie, & Wickings, K. (2017). Soil ecological responses to pest management in golf turf vary with management intensity, pesticide identity, and application program. *Agriculture, Ecosystems & Environment*, *246*, 66–77. doi:10.1016/j.agee.2017.05.014
186. García-Álvarez, A., Arias, M., Díez-Rojo, M. A., & Bello, A. (2004). Effect of agricultural management on soil nematode trophic structure in a Mediterranean cereal system. *Applied Soil Ecology: A Section of Agriculture, Ecosystems & Environment*, *27*(3), 197–210. doi:10.1016/j.apsoil.2004.06.002
187. García-Pérez, J. A., Alarcón-Gutiérrez, E., Perroni, Y., & Barois, I. (2014). Earthworm communities and soil properties in shaded coffee plantations with and without application of glyphosate. *Applied Soil Ecology: A Section of Agriculture, Ecosystems & Environment*, *83*, 230–237. doi:10.1016/j.apsoil.2013.09.006
188. García-Ruiz, R., Ochoa, V., Viñegla, B., Hinojosa, M. B., Peña-Santiago, R., Liébanas, G., … Carreira, J. A. (2009). Soil enzymes, nematode community and selected physico-chemical properties as soil quality indicators in organic and conventional olive oil farming: Influence of seasonality and site features. *Applied Soil Ecology: A Section of Agriculture, Ecosystems & Environment*, *41*(3), 305–314. doi:10.1016/j.apsoil.2008.12.004
189. García-Segura, D., Castillo-Murrieta, I. M., Martínez-Rabelo, F., Gomez-Anaya, A., Rodríguez-Campos, J., Hernández-Castellanos, B., … Barois, I. (2018). Macrofauna and mesofauna from soil contaminated by oil extraction. *Geoderma*, *332*, 180–189. doi:10.1016/j.geoderma.2017.06.013
190. George, P. B. L., & Lindo, Z. (2015). Application of body size spectra to nematode trait-index analyses. *Soil Biology & Biochemistry*, *84*, 15–20. doi:10.1016/j.soilbio.2015.02.007
191. Georgieva, S. S., McGrath, S. P., Hooper, D. J., & Chambers, B. S. (2002). Nematode communities under stress: the long-term effects of heavy metals in soil treated with sewage sludge. *Applied Soil Ecology: A Section of Agriculture, Ecosystems & Environment*, *20*(1), 27–42. doi:10.1016/s0929-1393(02)00005-7
192. Gillet, S., & Ponge, J.-F. (2005). Species assemblages and diets of Collembola in the organic matter accumulated over an old tar deposit. *European Journal of Soil Biology*, *41*(1–2), 39–44. doi:10.1016/j.ejsobi.2005.07.001
193. Gillet, S., & Ponge, J.-F. (2003). Changes in species assemblages and diets of Collembola along a gradient of metal pollution. *Applied Soil Ecology: A Section of Agriculture, Ecosystems & Environment*, *22*(2), 127–138. doi:10.1016/s0929-1393(02)00134-8
194. Gongalsky, K. B., Zaitsev, A. S., Korobushkin, D. I., Saifutdinov, R. A., Yazrikova, T. E., Benediktova, A. I., … Shakhab, S. V. (2016). Diversity of the soil biota in burned areas of southern taiga forests (Tver oblast). *Eurasian Soil Science*, *49*(3), 358–366. doi:10.1134/s1064229316030042
195. Gongalsky, K. B., Filimonova, Z. V., Pokarzhevskii, A. D., & Butovsky, R. O. (2007). Differences in responses of herpetobionts and geobionts to impact from the Kosogorsky Metallurgical Plant (Tula region, Russia). *Russian Journal of Ecology*, *38*(1), 52–57. doi:10.1134/s1067413607010092
196. Gongalsky, K.B. (2003). Impact of Pollution Caused by Uranium Production on Soil Macrofauna. *Environ Monit Assess 89*, 197–219. https://doi.org/10.1023/A:1026031224658
197. González-Macé, O., & Scheu, S. (2018). Response of Collembola and Acari communities to summer flooding in a grassland plant diversity experiment. *PloS One*, *13*(8), e0202862. doi:10.1371/journal.pone.0202862
198. Grabau, Z. J., & Chen, S. (2016). Influence of long-term corn–soybean crop sequences on soil ecology as indicated by the nematode community. *Applied Soil Ecology: A Section of Agriculture, Ecosystems & Environment*, *100*, 172–185. doi:10.1016/j.apsoil.2015.12.016

199. Greenwood, C. M., Barbercheck, M. E., & Brownie, C. (2011). Short term response of soil microinvertebrates to application of entomopathogenic nematode-infected insects in two tillage systems. *Pedobiologia*, *54*(3), 177–186. doi:10.1016/j.pedobi.2011.02.003
200. Gremmen, N. J. M., Chown, S. L., & Marshall, D. J. (1998). Impact of the introduced grass Agrostis stolonifera on vegetation and soil fauna communities at Marion Island, sub-Antarctic. *Biological Conservation*, *85*(3), 223–231. doi:10.1016/s0006-3207(97)00178-x
201. Gruss, I., Twardowski, J. P., & Hurej, M. (2018). Influence of 90-year potato and winter rye monocultures under different fertilisation on soil mites. *Ochrana Rostlin [Plant Protection Science]*, *54*(1), 31–38. doi:10.17221/46/2016-pps
202. Gunadi, B., Edwards, C. A., & Arancon, N. Q. (2002). Changes in trophic structure of soil arthropods after the application of vermicomposts. *European Journal of Soil Biology*, *38*(2), 161–165. doi:10.1016/s1164-5563(02)01139-1
203. Gutiérrez-López, M., Ranera, E., Novo, M., Fernández, R., & Trigo, D. (2014). Does the invasion of the exotic tree Ailanthus altissima affect the soil arthropod community? The case of a riparian forest of the Henares River (Madrid). *European Journal of Soil Biology*, *62*, 39–48. doi:10.1016/j.ejsobi.2014.02.010
204. Hagner, M., Romantschuk, M., Penttinen, O.-P., Egfors, A., Marchand, C., & Augustsson, A. (2018). Assessing toxicity of metal contaminated soil from glassworks sites with a battery of biotests. *The Science of the Total Environment*, *613–614*, 30–38. doi:10.1016/j.scitotenv.2017.08.121
205. Hågvar, S., & Klanderud, K. (2009). Effect of simulated environmental change on alpine soil arthropods. *Global Change Biology*, *15*(12), 2972–2980. doi:10.1111/j.1365-2486.2009.01926.x
206. Haimi, J., & Mätäsniemi, L. (2002). Soil decomposer animal community in heavy-metal contaminated coniferous forest with and without liming. *European Journal of Soil Biology*, *38*(2), 131–136. doi:10.1016/s1164-5563(02)01134-2
207. Haimi, J., Laamanen, J., Penttinen, R., Räty, M., Koponen, S., Kellomäki, S., & Niemelä, P. (2005). Impacts of elevated CO2 and temperature on the soil fauna of boreal forests. *Applied Soil Ecology: A Section of Agriculture, Ecosystems & Environment*, *30*(2), 104–112. doi:10.1016/j.apsoil.2005.02.006
208. Haimi, J., Fritze, H., & Moilanen, P. (2000). Responses of soil decomposer animals to wood-ash fertilisation and burning in a coniferous forest stand. *Forest Ecology and Management*, *129*(1–3), 53–61. doi:10.1016/s0378-1127(99)00158-9
209. Haimi, J., Siira-Pietikäinen, A. (1996). Decomposer animal communities in forest soil along heavy metal pollution gradient. *Fresenius J Anal Chem 354*, 672–675. doi:10.1007/s0021663540672
210. Han, D., Zhang, X., Tomar Vijay Vikram, S., Li, Q., Wen, D., & Liang, W. (2009). Effects of heavy metal pollution of highway origin on soil nematode guilds in North Shenyang, China. *Journal of Environmental Sciences (China)*, *21*(2), 193–198. doi:10.1016/s1001-0742(08)62250-0x
211. Hansen, R.A., Williams, R.S., Degenhardt, D.C., & Lincoln, D. E., (2001). Non-litter effects of elevated CO2 on forest floor microarthropod abundances. *Plant and Soil 236*, 139–144. https://doi.org/10.1023/A:1012705231992
212. Harkes, P., Verhoeven, A., Sterken, M. G., Snoek, L. B., van den Elsen, S. J. J., Mooijman, P. J. W., … Helder, J. (2017). The differential impact of a native and a non-native ragwort species (Senecioneae) on the first and second trophic level of the rhizosphere food web. *Oikos (Copenhagen, Denmark)*, *126*(12), 1790–1803. doi:10.1111/oik.04530
213. Hasegawa, M., Sasaki, T., Sato, H., & Abe, S. (2015). Effects of roads on collembolan community structure in subtropical evergreen forests on Okinawa Island, southwestern Japan. *Pedobiologia*, *58*(1), 13–21. doi:10.1016/j.pedobi.2014.11.002

214. Hasegawa, M., Ito, M. T., Yoshida, T., Seino, T., Chung, A. Y. C., & Kitayama, K. (2014). The effects of reduced-impact logging practices on soil animal communities in the Deramakot Forest Reserve in Borneo. *Applied Soil Ecology: A Section of Agriculture, Ecosystems & Environment*, *83*, 13–21. doi:10.1016/j.apsoil.2013.07.008
215. Haynes, R. J., Dominy, C. S., & Graham, M. H. (2003). Effect of agricultural land use on soil organic matter status and the composition of earthworm communities in KwaZulu-Natal, South Africa. *Agriculture, Ecosystems & Environment*, *95*(2–3), 453–464. doi:10.1016/s0167-8809(02)00223-2
216. Hedde, M., van Oort, F., & Lamy, I. (2012). Functional traits of soil invertebrates as indicators for exposure to soil disturbance. *Environmental Pollution*, *164*, 59–65. doi:10.1016/j.envpol.2012.01.017
217. Heiniger, C., Barot, S., Ponge, J.-F., Salmon, S., Botton-Divet, L., Carmignac, D., & Dubs, F. (2014). Effect of habitat spatiotemporal structure on collembolan diversity. *Pedobiologia*, *57*(2), 103–117. doi:10.1016/j.pedobi.2014.01.006
218. Hendrix, P. F., Mueller, B. R., Bruce, R. R., Langdale, G. W., & Parmelee, R. W. (1992). Abundance and distribution of earthworms in relation to landscape factors on the Georgia Piedmont, U.S.A. *Soil Biology & Biochemistry*, *24*(12), 1357–1361. doi:10.1016/0038-0717(92)90118-h
219. Heneghan, L., Salmore, A., & Crossley, D. A., Jr. (2004). Recovery of decomposition and soil microarthropod communities in an Appalachian watershed two decades after a clearcut. *Forest Ecology and Management*, *189*(1–3), 353–362. doi:10.1016/j.foreco.2003.09.002
220. Henneron, L., Bernard, L., Hedde, M., Pelosi, C., Villenave, C., Chenu, C., … Blanchart, E. (2015). Fourteen years of evidence for positive effects of conservation agriculture and organic farming on soil life. *Agronomy for Sustainable Development*, *35*(1), 169–181. doi:10.1007/s13593-014-0215-8
221. Hinojosa, M. B., Carreira, J. A., García-Ruíz, R., Rodríguez-Maroto, J. M., Daniell, T. J., & Griffiths, B. S. (2010). Plant treatment, pollutant load, and soil type effects in rhizosphere ecology of trace element polluted soils. *Ecotoxicology and Environmental Safety*, *73*(5), 970–981. doi:10.1016/j.ecoenv.2010.01.013
222. Hoeksema, J. D., Lussenhop, J., & Teeri, J. A. (2000). Soil nematodes indicate food web responses to elevated atmospheric CO2. *Pedobiologia*, *44*(6), 725–735. doi:10.1078/s0031-4056(04)70085-2
223. Hoekstha, J. M., Bell, R. T., Launer, A. E., & Murphy, D. D. (1995). Soil arthropod abundance in coast redwood forest: Effect of selective timber harvest. *Environmental Entomology*, *24*(2), 246–252. doi:10.1093/ee/24.2.246
224. Hof, A. R., & Bright, P. W. (2010). The impact of grassy field margins on macro-invertebrate abundance in adjacent arable fields. *Agriculture, Ecosystems & Environment*, *139*(1–2), 280–283. doi:10.1016/j.agee.2010.08.014
225. Hoffmann, B. D., Andersen, A. N., & Hill, G. J. E. (1999). Impact of an introduced ant on native rain forest invertebrates: *Oecologia*, *120*(4), 595. doi:10.1007/s004420050895
226. Holland, T. C., Reynolds, A. G., Bowen, P. A., Bogdanoff, C. P., Marciniak, M., Brown, R. B., & Hart, M. M. (2013). The response of soil biota to water availability in vineyards. *Pedobiologia*, *56*(1), 9–14. doi:10.1016/j.pedobi.2012.08.004
227. Holmstrup, M., Sørensen, J. G., Schmidt, I. K., Nielsen, P. L., Mason, S., Tietema, A., … Ehlers, B. K. (2013). Soil microarthropods are only weakly impacted after 13 years of repeated drought treatment in wet and dry heathland soils. *Soil Biology & Biochemistry*, *66*, 110–118. doi:10.1016/j.soilbio.2013.06.023

228. Holmstrup, M., Sørensen, J. G., Maraldo, K., Schmidt, I. K., Mason, S., Tietema, A., … Ehlers, B. K. (2012). Increased frequency of drought reduces species richness of enchytraeid communities in both wet and dry heathland soils. *Soil Biology & Biochemistry*, *53*, 43–49. doi:10.1016/j.soilbio.2012.05.001

229. Holmstrup, M., Schmelz, R. M., Carrera, N., Dyrnum, K., Larsen, K. S., Mikkelsen, T. N., & Beier, C. (2015). Responses of enchytraeids to increased temperature, drought and atmospheric CO2: Results of an eight-year field experiment in dry heathland. *European Journal of Soil Biology*, *70*, 15–22. doi:10.1016/j.ejsobi.2015.06.004

230. Holmstrup, M., Maraldo, K., & Krogh, P. H. (2007). Combined effect of copper and prolonged summer drought on soil microarthropods in the field. *Environmental Pollution*, *146*(2), 525–533. doi:10.1016/j.envpol.2006.07.013

231. Holmstrup, M., Ehlers, B. K., Slotsbo, S., Ilieva-Makulec, K., Sigurdsson, B. D., Leblans, N. I. W., … Berg, M. P. (2018). Functional diversity of Collembola is reduced in soils subjected to short-term, but not long-term, geothermal warming. *Functional Ecology*, *32*(5), 1304–1316. doi:10.1111/1365-2435.13058

232. Holmstrup, M., Damgaard, C., Schmidt, I. K., Arndal, M. F., Beier, C., Mikkelsen, T. N., … Christensen, S. (2017). Long-term and realistic global change manipulations had low impact on diversity of soil biota in temperate heathland. *Scientific Reports*, *7*(1). doi:10.1038/srep41388

233. Hoyle, M., & Harborne, A. R. (2005). Mixed effects of habitat fragmentation on species richness and community structure in a microarthropod microecosystem. *Ecological Entomology*, *30*(6), 684–691. doi:10.1111/j.0307-6946.2005.00738.x

234. Hu, Z., Zhu, C., Chen, X., Bonkowski, M., Griffiths, B., Chen, F., … Liu, M. (2017). Responses of rice paddy micro-food webs to elevated CO2 are modulated by nitrogen fertilization and crop cultivars. *Soil Biology & Biochemistry*, *114*, 104–113. doi:10.1016/j.soilbio.2017.07.008

235. Hu, J., Wu, J., Ma, M., Nielsen, U. N., Wang, J., & Du, G. (2015). Nematode communities response to long-term grazing disturbance on Tibetan plateau. *European Journal of Soil Biology*, *69*, 24–32. doi:10.1016/j.ejsobi.2015.04.003

236. Hu, J., Chen, G., Hassan, W. M., Chen, H., Li, J., & Du, G. (2017). Fertilization influences the nematode community through changing the plant community in the Tibetan Plateau. *European Journal of Soil Biology*, *78*, 7–16. doi:10.1016/j.ejsobi.2016.11.001

237. Hu, C., Wang, X. H., & Qi, Y. C. (2014). Characteristics of soil nematode communities under two different land use systems. *Biological Agriculture and Horticulture*, *30*(2), 119–130. doi:10.1080/01448765.2014.880373

238. Hu, Cheng, & Qi, Y.-C. (2011). Soil biological and biochemical quality of wheat-maize cropping system in long-term fertilizer experiments. *Experimental Agriculture*, *47*(4), 593–608. doi:10.1017/s0014479711000445

239. Hu, Cheng, & Qi, Y. (2010). Effect of compost and chemical fertilizer on soil nematode community in a Chinese maize field. *European Journal of Soil Biology*, *46*(3–4), 230–236. doi:10.1016/j.ejsobi.2010.04.002

240. Hu, Cheng, Hermann, G., Pen-Mouratov, S., Shore, L., & Steinberger, Y. (2011). Mammalian steroid hormones can reduce abundance and affect the sex ratio in a soil nematode community. *Agriculture, Ecosystems & Environment*, *142*(3–4), 275–279. doi:10.1016/j.agee.2011.05.024

241. Hu, Cheng, & Cao, Z.-P. (2008). Nematode community structure under compost and chemical fertilizer management practice, in the north China plain. *Experimental Agriculture*, *44*(4), 485–496. doi:10.1017/s0014479708006716

242. Hui, N., Selonen, S., Hanzel, J., Tuomela, M., Rainio, A., ….Romantschuk, M. (2009). Influence of lead on organisms within the ditritus food web of a contaminated pine forest soil. *Boreal Environment Research, 14*, 70-85.
243. Hulugalle, N. R., Lobry de Bruyn, L. A., & Entwistle, P. (1997). Residual effects of tillage and crop rotation on soil properties, soil invertebrate numbers and nutrient uptake in an irrigated Vertisol sown to cotton. *Applied Soil Ecology: A Section of Agriculture, Ecosystems & Environment*, *7*(1), 11–30. doi:10.1016/s0929-1393(97)00027-9
244. Hurisso, T. T., Davis, J. G., Brummer, J. E., Stromberger, M. E., Stonaker, F. H., Kondratieff, B. C., … Goldhamer, D. A. (2011). Earthworm abundance and species composition in organic forage production systems of northern Colorado receiving different soil amendments. *Applied Soil Ecology: A Section of Agriculture, Ecosystems & Environment*, *48*(2), 219–226. doi:10.1016/j.apsoil.2011.03.003
245. Hutchins, M. W., Reynolds, B. C., & Patch, S. P. (2011). Prescribed fire and the abundance of soil microarthropods in northeast Georgia. *Southeastern Naturalist (Steuben, Me.)*, *10*(3), 489–500. doi:10.1656/058.010.0308
246. Hylander, K., Nilsson, C., & Gothner, T. (2004). Effects of buffer-strip retention and clearcutting on land snails in boreal riparian forests. *Conservation Biology: The Journal of the Society for Conservation Biology*, *18*(4), 1052–1062. doi:10.1111/j.1523-1739.2004.00199.x
247. Hylander, K. (2011). The response of land snail assemblages below aspens to forest fire and clear-cutting in Fennoscandian boreal forests. *Forest Ecology and Management*, *261*(11), 1811–1819. doi:10.1016/j.foreco.2011.02.003
248. Ilieva-Makulec, K., Tyburski, J., & Makulec, G. (2016). Soil nematodes in organic and conventional farming system: A comparison of the taxonomic and functional diversity. *Polish Journal of Ecology*, *64*(4), 547–563. doi:10.3161/15052249pje2016.64.4.010
249. Iordache, M., & Borza, I. (2010). Relation between chemical indices of soil and earthworm abundance under chemical fertilization. *Plant, Soil and Environment*, *56*(9), 401–407. doi:10.17221/234/2009-pse
250. Ito, T., Araki, M., Komatsuzaki, M., Kaneko, N., & Ohta, H. (2015). Soil nematode community structure affected by tillage systems and cover crop managements in organic soybean production. *Applied Soil Ecology: A Section of Agriculture, Ecosystems & Environment*, *86*, 137–147. doi:10.1016/j.apsoil.2014.10.003
251. Ivask, M., Kuu, A., & Sizov, E. (2007). Abundance of earthworm species in Estonian arable soils. *European Journal of Soil Biology*, *43*, S39–S42. doi:10.1016/j.ejsobi.2007.08.006
252. Jamatia, S. K. S., & Chaudhuri, P. S., (2017). Species diversity and community characteristics of earthworms in managed and degraded tea plantations of Tripura. *Journal of Environmental Biology*, *38*(6), 1349–1356. doi:10.22438/jeb/38/6/mrn-416
253. Jamshidian, M. K., Saboori, A., Akrami, M. A., & van Straalen, N. (2015). Oribatid mite communities in contamindated soils nearby a lead and zinc smelting plant in Zanjan, Iran. *Systematic & Applied Acarology, 20*(3), 251-262. doi:10.11158/saa.20.3.3
254. Jandl, R., Kopeszki, H., Bruckner, A., & Hager, H. (2003). Forest soil chemistry and mesofauna 20 years after an amelioration fertilization. *Restoration Ecology*, *11*(2), 239–246. doi:10.1046/j.1526-100x.2003.00179.x
255. Jiang, Y., Sun, B., Jin, C., & Wang, F. (2013). Soil aggregate stratification of nematodes and microbial communities affects the metabolic quotient in an acid soil. *Soil Biology & Biochemistry*, *60*, 1–9. doi:10.1016/j.soilbio.2013.01.006
256. Jiang, Y., Ma, N., Chen, Z., & Xie, H. (2018). Soil macrofauna assemblage composition and functional groups in no-tillage with corn stover mulch agroecosystems in a mollisol area of northeastern

China. *Applied Soil Ecology: A Section of Agriculture, Ecosystems & Environment*, *128*, 61–70. doi:10.1016/j.apsoil.2018.04.006

257. Jiang, M., Wang, X., Liusui, Y., Sun, X., Zhao, C., & Liu, H. (2015). Diversity and abundance of soil animals as influenced by long-term fertilization in grey desert soil, China. *Sustainability*, *7*(8), 10837–10853. doi:10.3390/su70810837

258. Jiang, C., Sun, B., Li, H., & Jiang, Y. (2013). Determinants for seasonal change of nematode community composition under long-term application of organic manure in an acid soil in subtropical China. *European Journal of Soil Biology*, *55*, 91–99. doi:10.1016/j.ejsobi.2012.11.003

259. Johnson-Maynard, J., Umiker, K., & Guy, S. (2007). Earthworm dynamics and soil physical properties in the first three years of no-till management. *Soil & Tillage Research*, *94*(2), 338–345. doi:10.1016/j.still.2006.08.011

260. Jones, H. D., Santoro, G., Boag, B., & Neilson, R. (2001). The diversity of earthworms in 200 Scottish fields and the possible effect of New Zealand land flatworms (Arthurdendyus triangulatus) on earthworm populations. *The Annals of Applied Biology*, *139*(1), 75–92. doi:10.1111/j.1744-7348.2001.tb00132.x

261. Jourgholami, M., Nasirian, A., & Labelle, E. (2018). Ecological restoration of compacted soil following the application of different leaf litter mulches on the skid trail over a five-year period. *Sustainability*, *10*(7), 2148. doi:10.3390/su10072148

262. Jucevica, E., & Melecis, V. (2006). Global warming affect Collembola community: A long-term study. *Pedobiologia*, *50*(2), 177–184. doi:10.1016/j.pedobi.2005.10.006

263. Kaneda, S., Yamashita, N., Uchida, T., Shimano, S., Miyoshi, N., Sasaki, M., & Enami, Y. (2006). Effects of ivermectin in dung pats on earthworm (Megascolecidae) populations and pat degradation in Japanese grassland. *Applied Soil Ecology: A Section of Agriculture, Ecosystems & Environment*, *31*(3), 280–285. doi:10.1016/j.apsoil.2005.05.001

264. Kaneda, S., Miura, S., Yamashita, N., Ohigashi, K., Yamasaki, S., Murakami, T., & Urashima, Y. (2012). Significance of litter layer in enhancing mesofaunal abundance and microbial biomass nitrogen in sweet corn-white clover living mulch systems. *Soil Science and Plant Nutrition*, *58*(4), 424–434. doi:10.1080/00380768.2012.699881

265. Kanianska, R., Jaďuďová, J., Makovníková, J., & Kizeková, M. (2016). Assessment of relationships between earthworms and soil abiotic and biotic factors as a tool in sustainable agricultural. *Sustainability*, *8*(9), 906. doi:10.3390/su8090906

266. Kapusta, P., Szarek-Łukaszewska, G., & Stefanowicz, A. M. (2011). Direct and indirect effects of metal contamination on soil biota in a Zn-Pb post-mining and smelting area (S Poland). *Environmental Pollution*, *159*(6), 1516–1522. doi:10.1016/j.envpol.2011.03.015

267. Kardol, P., Reynolds, W. N., Norby, R. J., & Classen, A. T. (2011). Climate change effects on soil microarthropod abundance and community structure. *Applied Soil Ecology: A Section of Agriculture, Ecosystems & Environment*, *47*(1), 37–44. doi:10.1016/j.apsoil.2010.11.001

268. Kardol, P., Cregger, M. A., Campany, C. E., & Classen, A. T. (2010). Soil ecosystem functioning under climate change: plant species and community effects. *Ecology*, *91*(3), 767–781. doi:10.1890/09-0135.1

269. Kautz, T., López-Fando, C., & Ellmer, F. (2006). Abundance and biodiversity of soil microarthropods as influenced by different types of organic manure in a long-term field experiment in Central Spain. *Applied Soil Ecology: A Section of Agriculture, Ecosystems & Environment*, *33*(3), 278–285. doi:10.1016/j.apsoil.2005.10.003

270. Kennedy, A. D. (1994). Simulated climate change: a field manipulation study of polar microarthropod community response to global warming. *Ecography*, *17*(2), 131–140. doi:10.1111/j.1600-0587.1994.tb00085.x
271. Khalil, M. A., Al-Assiuty, A.-N. I. M., van Straalen, N. M., & Al-Assiuty, B. A. (2016). Changes in soil oribatid communities associated with conversion from conventional to organic agriculture. *Experimental & Applied Acarology*, *68*(2), 183–196. doi:10.1007/s10493-015-9979-z
272. Klamerus-Iwan, A., Błońska, E., Lasota, J., Kalandyk, A., & Waligórski, P. (2015). Influence of oil contamination on physical and biological properties of forest soil after chainsaw use. *Water, Air, and Soil Pollution*, *226*(11), 389. doi:10.1007/s11270-015-2649-2
273. Klarner, B., Winkelmann, H., Krashevska, V., Maraun, M., Widyastuti, R., & Scheu, S. (2017). Trophic niches, diversity and community composition of invertebrate top predators (Chilopoda) as affected by conversion of tropical lowland rainforest in Sumatra (Indonesia). *PloS One*, *12*(8), e0180915. doi:10.1371/journal.pone.0180915
274. Klok, C., & Thissen, J. (2009). Are laboratory derived toxicity results informative for field situations? Case study on earthworm populations contaminated with heavy metals. *Soil Biology & Biochemistry*, *41*(2), 251–255. doi:10.1016/j.soilbio.2008.10.019
275. Koenning S. R., & Barker K.R. (2004). Influence of poultry litter applications on nematode communities in cotton agroecosystems. *The Journal of Nematology, 36*(4), 524-33.
276. KokoŘová, P., & Starý, J. (2017). Communities of oribatid mites (Acari: Oribatida) of naturally regenerating and salvage-logged montane spruce forests of Šumava Mountains. *Biologia*, *72*(4), 445–451. doi:10.1515/biolog-2017-0050
277. Konestabo, H. S., Michelsen, A., & Holmstrup, M. (2007). Responses of springtail and mite populations to prolonged periods of soil freeze-thaw cycles in a sub-arctic ecosystem. *Applied Soil Ecology: A Section of Agriculture, Ecosystems & Environment*, *36*(2–3), 136–146. doi:10.1016/j.apsoil.2007.01.003
278. Koolhaas, J.E., Van Gestel, C.A., Römbke, J., Soares, A. M. V. M., & Jones, S. E., (2004). Ring-Testing and Field-validation of a Terrestrial Model Ecosystem (TME) – An Instrument for Testing Potentially Harmful Substances: Effects of Carbendazim on Soil Microarthropod Communities. *Ecotoxicology 13*, 75–88. https://doi.org/10.1023/B:ECTX.0000012406.93004.03
279. Kools, S. A. E., Boivin, M.-E. Y., Van Der Wurff, A. W. G., Berg, M. P., Van Gestel, C. A. M., & Van Straalen, N. M. (2009). Assessment of structure and function in metal polluted grasslands using Terrestrial Model Ecosystems. *Ecotoxicology and Environmental Safety*, *72*(1), 51–59. doi:10.1016/j.ecoenv.2008.03.016
280. Kools, S. A. E., Berg, M. P., Boivin, M.-E. Y., Kuenen, F. J. A., van der Wurff, A. W. G., van Gestel, C. A. M., & van Straalen, N. M. (2008). Stress responses investigated; application of zinc and heat to Terrestrial Model Ecosystems from heavy metal polluted grassland. *The Science of the Total Environment*, *406*(3), 462–468. doi:10.1016/j.scitotenv.2008.06.057
281. Korthals, G.W., Bongers, M., Fokkema, A., Dueck T. A., & Lexmond, T. M., (2000). Joint Toxicity of Copper and Zinc to a Terrestrial Nematode Community in an Acid Sandy Soil. Ecotoxicology 9, 219–228 (2000). https://doi.org/10.1023/A:1008950905983
282. Kotiaho, J. S., & Sulkava, P. (2007). Effects of isolation, area and predators on invasion: A field experiment with artificial islands. *Applied Soil Ecology: A Section of Agriculture, Ecosystems & Environment*, *35*(1), 256–259. doi:10.1016/j.apsoil.2006.05.003
283. Kragten, S., Tamis, W. L. M., Gertenaar, E., Midcap Ramiro, S. M., Van Der Poll, R. J., Wang, J., & de Snoo, G. R. (2011). Abundance of invertebrate prey for birds on organic and conventional

arable farms in the Netherlands. *Bird Conservation International*, *21*(1), 1–11. doi:10.1017/s0959270910000079
284. Kukkonen, S., Palojärvi, A., Räkköläinen, M., & Vestberg, M. (2004). Peat amendment and production of different crop plants affect earthworm populations in field soil. *Soil Biology & Biochemistry*, *36*(3), 415–423. doi:10.1016/j.soilbio.2003.10.017
285. Kunkel, B. A., Held, D. W., & Potter, D. A. (1999). Impact of halofenozide, imidacloprid, and bendiocarb on beneficial invertebrates and predatory activity in turfgrass. *Journal of Economic Entomology*, *92*(4), 922–930. doi:10.1093/jee/92.4.922
286. Kuperman, R. G., Phillips, C. T., & Checkai, R. T. (2007). Toxicity of chemical warfare agent HD (mustard) to the soil microinvertebrate community in natural soils with contrasting properties. *Pedobiologia*, *50*(6), 535–542. doi:10.1016/j.pedobi.2006.10.004
287. Kuznetsova, N. A. (2009). Soil-dwelling Collembola in coniferous forests along the gradient of pollution with emissions from the Middle Ural Copper Smelter. *Russian Journal of Ecology*, *40*(6), 415–423. doi:10.1134/s106741360906006x
288. Lamandé, M., Eriksen, J., Krogh, P. H., & Jacobsen, O. H. (2017). Water flow in soil from organic dairy rotations. *The Journal of Agricultural Science*, *155*(7), 1113–1123. doi:10.1017/s0021859617000089
289. Lammel, D. R., Azevedo, L. C. B., Paula, A. M., Armas, R. D., Baretta, D., & Cardoso, E. J. B. N. (2015). Microbiological and faunal soil attributes of coffee cultivation under different management systems in Brazil. *Brazilian Journal of Biology*, *75*(4), 894–905. doi:10.1590/1519-6984.02414
290. Landesman, W. J., Treonis, A. M., & Dighton, J. (2011). Effects of a one-year rainfall manipulation on soil nematode abundances and community composition. *Pedobiologia*, *54*(2), 87–91. doi:10.1016/j.pedobi.2010.10.002
291. Landi, S., Papini, R., d'Errico, G., Brandi, G., Rocchini, A., Roversi, P. F., … Mocali, S. (2018). Effect of different set-aside management systems on soil nematode community and soil fertility in North, Central and South Italy. *Agriculture, Ecosystems & Environment*, *261*, 251–260. doi:10.1016/j.agee.2018.01.003
292. Landi, S., Papini, R., d'Errico, G., Barzanti, G. P., & Roversi, P. F. (2018). Nematode indicators as integrative measures of soil condition during conversion from conventional to organic rice production in Italy: a case study. *Biological Agriculture and Horticulture*, *34*(3), 141–153. doi:10.1080/01448765.2017.1399166
293. Lagerlöf, J., Pålsson, O., & Arvidsson, J. (2012). Earthworms influenced by reduced tillage, conventional tillage and energy forest in Swedish agricultural field experiments. *Acta Agriculturae Scandinavica. Section B, Soil and Plant Science*, *62*(3), 235–244. doi:10.1080/09064710.2011.602717
294. Larson, J. L., Redmond, C. T., & Potter, D. A. (2012). Comparative impact of an anthranilic diamide and other insecticidal chemistries on beneficial invertebrates and ecosystem services in turfgrass. *Pest Management Science*, *68*(5), 740–748. doi:10.1002/ps.2321
295. Lazzaro, L., Mazza, G., d'Errico, G., Fabiani, A., Giuliani, C., Inghilesi, A. F., … Foggi, B. (2018). How ecosystems change following invasion by Robinia pseudoacacia: Insights from soil chemical properties and soil microbial, nematode, microarthropod and plant communities. *The Science of the Total Environment*, *622–623*, 1509–1518. doi:10.1016/j.scitotenv.2017.10.017
296. Lees, K. J., McKenzie, A. J., Newell Price, J. P., Critchley, C. N., Rhymer, C. M., Chambers, B. J., & Whittingham, M. J. (2016). The effects of soil compaction mitigation on below-ground fauna: How earthworms respond to mechanical loosening and power harrow cultivation. *Agriculture, Ecosystems & Environment*, *232*, 273–282. doi:10.1016/j.agee.2016.07.026

297. Lemanski, K., & Scheu, S. (2015). The influence of fertilizer addition, cutting frequency and herbicide application on soil organisms in grassland. *Biology and Fertility of Soils*, *51*(2), 197–205. doi:10.1007/s00374-014-0963-2

298. Lenz, R., & Eisenbeis, G. (2000). Short-term effects of different tillage in a sustainable farming system on nematode community structure. *Biology and Fertility of Soils*, *31*(3–4), 237–244. doi:10.1007/s003740050651

299. Leroy, B. L. M., Van den Bossche, A., De Neve, S., Reheul, D., & Moens, M. (2007). The quality of exogenous organic matter: Short-term influence on earthworm abundance. *European Journal of Soil Biology*, *43*, S196–S200. doi:10.1016/j.ejsobi.2007.08.015

300. Leroy, B. L. M., Schmidt, O., Van den Bossche, A., Reheul, D., & Moens, M. (2008). Earthworm population dynamics as influenced by the quality of exogenous organic matter. *Pedobiologia*, *52*(2), 139–150. doi:10.1016/j.pedobi.2008.07.001

301. Lévêque, T., Capowiez, Y., Schreck, E., Mombo, S., Mazzia, C., Foucault, Y., & Dumat, C. (2015). Effects of historic metal(loid) pollution on earthworm communities. *The Science of the Total Environment*, *511*, 738–746. doi:10.1016/j.scitotenv.2014.11.101

302. Li, Yuanyuan, Chen, Y., Xu, C., Xu, H., Zou, X., Chen, H. Y. H., & Ruan, H. (2018). The abundance and community structure of soil arthropods in reclaimed coastal saline soil of managed poplar plantations. *Geoderma*, *327*, 130–137. doi:10.1016/j.geoderma.2018.05.004

303. Li, Yiqing, Shimabukuro-Madden, A., & Mathews, B. W. (2010). The effects of tree plantation rotation on earthworm abundance and biomass in Hawaii. *Applied Soil Ecology: A Section of Agriculture, Ecosystems & Environment*, *46*(1), 151–154. doi:10.1016/j.apsoil.2010.07.003

304. Li, Yufei, Li, J., Zheng, C., & Cao, Z. (2014). Effects of organic, low input, conventional management practices on soil nematode community under greenhouse conditions. *Acta Agriculturae Scandinavica. Section B, Soil and Plant Science*, *64*(4), 360–371. doi:10.1080/09064710.2014.911953

305. Li, X., Liu, Q., Liu, Z., Shi, W., Yang, D., & Tarasco, E. (2014). Effects of organic and other management practices on soil nematode communities in tea plantation: a case study in southern China. *Journal of Plant Nutrition and Soil Science*, *177*(4), 604–612. doi:10.1002/jpln.201300610

306. Li, X.-G., Wang, X.-X., Dai, C.-C., Zhang, T.-L., Xie, X.-G., Ding, C.-F., & Wang, H.-W. (2014). Effects of intercropping with Atractylodes lancea and application of bio-organic fertiliser on soil invertebrates, disease control and peanut productivity in continuous peanut cropping field in subtropical China. *Agroforestry Systems*, *88*(1), 41–52. doi:10.1007/s10457-013-9653-6

307. Li, Q., Yang, Y., Bao, X., Zhu, J., Liang, W., & Bezemer, T. M. (2016). Cultivar specific plant-soil feedback overrules soil legacy effects of elevated ozone in a rice-wheat rotation system. *Agriculture, Ecosystems & Environment*, *232*, 85–92. doi:10.1016/j.agee.2016.07.025

308. Li, Q., Jiang, Y., Liang, W., Lou, Y., Zhang, E., & Liang, C. (2010). Long-term effect of fertility management on the soil nematode community in vegetable production under greenhouse conditions. *Applied Soil Ecology: A Section of Agriculture, Ecosystems & Environment*, *46*(1), 111–118. doi:10.1016/j.apsoil.2010.06.016

309. Li, Q., Bao, X., Lu, C., Zhang, X., Zhu, J., Jiang, Y., & Liang, W. (2012). Soil microbial food web responses to free-air ozone enrichment can depend on the ozone-tolerance of wheat cultivars. *Soil Biology & Biochemistry*, *47*, 27–35. doi:10.1016/j.soilbio.2011.12.012

310. Li, Q., Xu, C., Liang, W., Zhong, S., Zheng, X., & Zhu, J. (2009). Residue incorporation and N fertilization affect the response of soil nematodes to the elevated CO2 in a Chinese wheat field. *Soil Biology & Biochemistry*, *41*(7), 1497–1503. doi:10.1016/j.soilbio.2009.04.006

311. Li, Q., Liang, W., Jiang, Y., Shi, Y., Zhu, J., & Neher, D. A. (2007). Effect of elevated CO2 and N fertilisation on soil nematode abundance and diversity in a wheat field. *Applied Soil Ecology: A Section of Agriculture, Ecosystems & Environment*, *36*(1), 63–69. doi:10.1016/j.apsoil.2006.11.003
312. Li, J., Hong, M., Yin, X., & Liu, J. (2010). Effects of the accumulation of the rare earth elements on soil macrofauna community. *Journal of Rare Earths*, *28*(6), 957–964. doi:10.1016/s1002-0721(09)60233-7
313. Li, J., Wang, D., Fan, W., He, R., Yao, Y., Sun, L., Zhao, X., & Wu, J. (2018). Comparative effects of different organic materials on nematode community in continuous soybean monoculture soil. *Applied Soil Ecology: A Section of Agriculture, Ecosystems & Environment*, *125*, 12–17. doi:10.1016/j.apsoil.2017.12.013
314. Li, Q., Zhong, S., Li, F., Lou, Y., & Liang, W. (2011). Nematode community structure as bioindicator of soil heavy metal pollution along an urban-rural gradient in southern Shenyang, China. *International Journal of Environment and Pollution, 45*(4), 297-309
315. Liang, W.-J., Zhong, S., Hua, J.-F., Cao, C.-Y., & Jiang, Y. (2007). Nematode faunal response to grassland degradation in horqin Sandy Land. *Pedosphere*, *17*(5), 611–618. doi:10.1016/s1002-0160(07)60072-1
316. Liang, W., Lou, Y., Li, Q., Zhong, S., Zhang, X., & Wang, J. (2009). Nematode faunal response to long-term application of nitrogen fertilizer and organic manure in Northeast China. *Soil Biology & Biochemistry*, *41*(5), 883–890. doi:10.1016/j.soilbio.2008.06.018
317. Liang, W., Li, Q., Jiang, Y., & Neher, D. A. (2005). Nematode faunal analysis in an aquic brown soil fertilised with slow-release urea, Northeast China. *Applied Soil Ecology: A Section of Agriculture, Ecosystems & Environment*, *29*(2), 185–192. doi:10.1016/j.apsoil.2004.10.004
318. Liang, W., Beattie, G. A. C., Meats, A., & Spooner-Hart, R. (2007). Impact on soil-dwelling arthropods in citrus orchards of spraying horticultural mineral oil, carbaryl or methidathion. *Australian Journal of Entomology*, *46*(1), 79–85. doi:10.1111/j.1440-6055.2007.00583.x
319. Liiri, M., Setälä, H., Haimi, J., Pennanen, T., & Fritze, H. (2002). Soil processes are not influenced by the functional complexity of soil decomposer food webs under disturbance. *Soil Biology & Biochemistry*, *34*(7), 1009–1020. doi:10.1016/s0038-0717(02)00034-2
320. Liiri, M., Haimi, J., & Setälä, H. (2002). Community composition of soil microarthropods of acid forest soils as affected by wood ash application. *Pedobiologia*, *46*(2), 108–124. doi:10.1078/0031-4056-00118
321. Lindberg, N., & Persson, T. (2004). Effects of long-term nutrient fertilisation and irrigation on the microarthropod community in a boreal Norway spruce stand. *Forest Ecology and Management*, *188*(1–3), 125–135. doi:10.1016/j.foreco.2003.07.012
322. Lindberg, N., & Bengtsson, J. (2006). Recovery of forest soil fauna diversity and composition after repeated summer droughts. *Oikos (Copenhagen, Denmark)*, *114*(3), 494–506. doi:10.1111/j.2006.0030-1299.14396.x
323. Lindo, Z. (2015). Warming favours small-bodied organisms through enhanced reproduction and compositional shifts in belowground systems. *Soil Biology & Biochemistry*, *91*, 271–278. doi:10.1016/j.soilbio.2015.09.003
324. Lindsay, E. A., & French, K. (2006). The Impact of the Weed Chrysanthemoides monilifera ssp. rotundata on Coastal Leaf Litter Invertebrates. *Biological Invasions*, *8*(2), 177–192. doi:10.1007/s10530-004-5856-0
325. Lins, V. S., Santos, H. R., & Gonçalves, M. C. (2007). The effect of the glyphosate, 2,4-D, atrazine e nicosulfuron herbicides upon the Edaphic collembola (Arthropoda: Ellipura) in a no tillage system. *Neotropical Entomology*, *36*(2), 261–267. doi:10.1590/s1519-566x2007000200013

326. Liu, W., Zhang, J., Norris, S. L., & Murray, P. J. (2016). Impact of grassland reseeding, herbicide spraying and ploughing on diversity and abundance of soil arthropods. *Frontiers in Plant Science*, *7*, 1200. doi:10.3389/fpls.2016.01200

327. Liu, T., Whalen, J. K., Shen, Q., & Li, H. (2016). Increase in soil nematode abundance due to fertilization was consistent across moisture regimes in a paddy rice–upland wheat system. *European Journal of Soil Biology*, *72*, 21–26. doi:10.1016/j.ejsobi.2015.12.001

328. Liu, S., Yang, X., Ives, A. R., Feng, Z., & Sha, L. (2017). Effects of seasonal and perennial grazing on soil fauna community and microbial biomass carbon in the subalpine meadows of Yunnan, southwest China. *Pedosphere*, *27*(2), 371–379. doi:10.1016/s1002-0160(17)60325-4

329. Liu, R.-T., Zhao, H.-L., Zhao, X.-Y., & Zhu, F. (2013). Effects of cultivation and grazing exclusion on the soil macro-faunal community of semiarid sandy grasslands in northern China. *Arid Land Research Management*, *27*(4), 377–393. doi:10.1080/15324982.2013.787470

330. Liu, M., Chen, X., Qin, J., Wang, D., Griffiths, B., & Hu, F. (2008). A sequential extraction procedure reveals that water management affects soil nematode communities in paddy fields. *Applied Soil Ecology: A Section of Agriculture, Ecosystems & Environment*, *40*(2), 250–259. doi:10.1016/j.apsoil.2008.05.001

331. Liu, M., Chen, X., Griffiths, B. S., Huang, Q., Li, H., & Hu, F. (2012). Dynamics of nematode assemblages and soil function in adjacent restored and degraded soils following disturbance. *European Journal of Soil Biology*, *49*, 37–46. doi:10.1016/j.ejsobi.2011.09.004

332. Lobe, J. W., Callaham, M. A., Jr, Hendrix, P. F., & Hanula, J. L. (2014). Removal of an invasive shrub (Chinese privet: Ligustrum sinense Lour) reduces exotic earthworm abundance and promotes recovery of native North American earthworms. *Applied Soil Ecology: A Section of Agriculture, Ecosystems & Environment*, *83*, 133–139. doi:10.1016/j.apsoil.2014.03.020

333. Loranger, G. I., Pregitzer, K. S., & King, J. S. (2004). Elevated CO2 and O3t concentrations differentially affect selected groups of the fauna in temperate forest soils. *Soil Biology & Biochemistry*, *36*(9), 1521–1524. doi:10.1016/j.soilbio.2004.04.022

334. Lukkari, T., Taavitsainen, M., Väisänen, A., & Haimi, J. (2004). Effects of heavy metals on earthworms along contamination gradients in organic rich soils. *Ecotoxicology and Environmental Safety*, *59*(3), 340–348. doi:10.1016/j.ecoenv.2003.09.011

335. Lundgren, J. G., Hesler, L. S., Clay, S. A., & Fausti, S. F. (2013). Insect communities in soybeans of eastern South Dakota: The effects of vegetation management and pesticides on soybean aphids, bean leaf beetles, and their natural enemies. *Crop Protection (Guildford, Surrey)*, *43*, 104–118. doi:10.1016/j.cropro.2012.08.005

336. Lupatini, M., Korthals, G. W., Roesch, L. F. W., & Kuramae, E. E. (2019). Long-term farming systems modulate multi-trophic responses. *The Science of the Total Environment*, *646*, 480–490. doi:10.1016/j.scitotenv.2018.07.323

337. Maceda-Veiga, A., Basas, H., Lanzaco, G., Sala, M., de Sostoa, A., & Serra, A. (2016). Impacts of the invader giant reed (Arundo donax) on riparian habitats and ground arthropod communities. *Biological Invasions*, *18*(3), 731–749. doi:10.1007/s10530-015-1044-7

338. Madritch, M. D., & Lindroth, R. L. (2009). Removal of invasive shrubs reduces exotic earthworm populations. *Biological Invasions*, *11*(3), 663–671. doi:10.1007/s10530-008-9281-7

339. Maisto, G., Santorufo, L., Milano, V., & Arena, C. (2016). Relationships between Quercus ilex L. litter characteristics and soil microarthropod community in an urban environment at different climatic conditions. *Applied Soil Ecology: A Section of Agriculture, Ecosystems & Environment*, *99*, 98–109. doi:10.1016/j.apsoil.2015.11.025

340. Makkonen, M., Berg, M. P., van Hal, J. R., Callaghan, T. V., Malcolm C. Press, & Aerts, R. (2011). Traits explain the responses of a sub-arctic Collembola community to climate manipulation. *Soil Biology & Biochemistry*, *43*(2), 377–384. doi:10.1016/j.soilbio.2010.11.004

341. Malmström, A., Persson, T., Ahlström, K., Gongalsky, K. B., & Bengtsson, J. (2009). Dynamics of soil meso- and macrofauna during a 5-year period after clear-cut burning in a boreal forest. *Applied Soil Ecology: A Section of Agriculture, Ecosystems & Environment*, *43*(1), 61–74. doi:10.1016/j.apsoil.2009.06.002

342. Malmström, A., Persson, T., & Ahlström, K. (2008). Effects of fire intensity on survival and recovery of soil microarthropods after a clearcut burning. *Canadian Journal of Forest Research*, *38*(9), 2465–2475. doi:10.1139/x08-094

343. Manono, B. O., & Moller, H. (2015). Effects of stock type, irrigation and effluent dispersal on earthworm species composition, densities and biomasses in New Zealand pastures. *Pedobiologia*, *58*(5–6), 187–193. doi:10.1016/j.pedobi.2015.09.002

344. Maraldo, K., Krogh, P. H., van der Linden, L., Christensen, B., Mikkelsen, T. N., Beier, C., & Holmstrup, M. (2010). The counteracting effects of elevated atmospheric CO2 concentrations and drought episodes: Studies of enchytraeid communities in a dry heathland. *Soil Biology & Biochemistry*, *42*(11), 1958–1966. doi:10.1016/j.soilbio.2010.07.015

345. Maraldo, K., & Holmstrup, M. (2009). Recovery of enchytraeid populations after severe drought events. *Applied Soil Ecology: A Section of Agriculture, Ecosystems & Environment*, *42*(3), 227–235. doi:10.1016/j.apsoil.2009.04.004

346. Maraldo, K., Christensen, B., Strandberg, B., & Holmstrup, M. (2006). Effects of copper on enchytraeids in the field under differing soil moisture regimes. *Environmental Toxicology and Chemistry*, *25*(2), 604–612. doi:10.1897/05-076r.1

347. Mariotte, P., Le Bayon, R.-C., Eisenhauer, N., Guenat, C., & Buttler, A. (2016). Subordinate plant species moderate drought effects on earthworm communities in grasslands. *Soil Biology & Biochemistry*, *96*, 119–127. doi:10.1016/j.soilbio.2016.01.020

348. Marra, J. L., & Edmonds, R. L. (1998). Effects of coarse woody debris and soil depth on the density and diversity of soil invertebrates on clearcut and forested sites on the Olympic peninsula, Washington. *Environmental Entomology*, *27*(5), 1111–1124. doi:10.1093/ee/27.5.1111

349. Martinez, J. G., Torres, M. A., dos Santos, G., & Moens, T. (2018). Influence of heavy metals on nematode community structure in deteriorated soil by gold mining activities in Sibutad, southern Philippines. *Ecological Indicators*, *91*, 712–721. doi:10.1016/j.ecolind.2018.04.021

350. Marwitz, A., Ladewig, E., & Märländer, B. (2012). Impact of herbicide application intensity in relation to environment and tillage on earthworm population in sugar beet in Germany. *European Journal of Agronomy: The Journal of the European Society for Agronomy*, *39*, 25–34. doi:10.1016/j.eja.2012.01.005

351. McGrath, D. A., & Binkley, M. A. (2009). Microstegium vimineumInvasion changes soil chemistry and microarthropod communities in Cumberland plateau forests. *Southeastern Naturalist (Steuben, Me.)*, *8*(1), 141–156. doi:10.1656/058.008.0113

352. Menz, L., Gibb, H., & Murphy, N. P. (2016). Dispersal-limited detritivores in fire-prone environments: persistence and population structure of terrestrial amphipods (Talitridae). *International Journal of Wildland Fire*, *25*(7), 753. doi:10.1071/wf15005

353. Migliorini, M., Pigino, G., Caruso, T., Fanciulli, P. P., Leonzio, C., & Bernini, F. (2005). Soil communities (Acari Oribatida; Hexapoda Collembola) in a clay pigeon shooting range. *Pedobiologia*, *49*(1), 1–13. doi:10.1016/j.pedobi.2004.06.009

354. Mijangos, I., Pérez, R., Albizu, I., & Garbisu, C. (2006). Effects of fertilization and tillage on soil biological parameters. *Enzyme and Microbial Technology*, *40*(1), 100–106. doi:10.1016/j.enzmictec.2005.10.043
355. Mikola, J., Yeates, G. W., Barker, G. M., Wardle, D. A., & Bonner, K. I. (2001). Effects of defoliation intensity on soil food-web properties in an experimental grassland community. *Oikos (Copenhagen, Denmark)*, *92*(2), 333–343. doi:10.1034/j.1600-0706.2001.920216.x
356. Mikola, J., Sørensen, L. I., & Kytöviita, M.-M. (2014). Plant removal disturbance and replant mitigation effects on the abundance and diversity of low-arctic soil biota. *Applied Soil Ecology: A Section of Agriculture, Ecosystems & Environment*, *82*, 82–92. doi:10.1016/j.apsoil.2014.05.013
357. Mikola, J., Nieminen, M., Ilmarinen, K., & Vestberg, M. (2005). Belowground responses by AM fungi and animal trophic groups to repeated defoliation in an experimental grassland community. *Soil Biology & Biochemistry*, *37*(9), 1630–1639. doi:10.1016/j.soilbio.2005.01.027
358. Miller, J. J., Battigelli, J. P., & Willms, W. D. (2014). Grazing protection influences soil mesofauna in ungrazed and grazed riparian and upland pastures. *Rangeland Ecology & Management*, *67*(4), 429–434. doi:10.2111/rem-d-14-00004.1
359. Miller, J. J., Battigelli, J. P., Beasley, B. W., & Drury, C. F. (2017). Response of soil mesofauna to long-term application of feedlot manure on irrigated cropland. *Journal of Environmental Quality*, *46*(1), 185–192. doi:10.2134/jeq2016.08.0318
360. Minoshima, H., Jackson, L. E., Cavagnaro, T. R., Sánchez-Moreno, S., Ferris, H., Temple, S. R., … Mitchell, J. P. (2007). Soil food webs and carbon dynamics in response to conservation tillage in California. *Soil Science Society of America Journal. Soil Science Society of America*, *71*(3), 952–963. doi:10.2136/sssaj2006.0174
361. Minor, M. A., Volk, T. A., & Norton, R. A. (2004). Effects of site preparation techniques on communities of soil mites (Acari: Oribatida, Acari: Gamasida) under short-rotation forestry plantings in New York, USA. *Applied Soil Ecology: A Section of Agriculture, Ecosystems & Environment*, *25*(3), 181–192. doi:10.1016/j.apsoil.2003.10.002
362. Minor, M. A., & Norton, R. A. (2008). Effects of weed and erosion control on communities of soil mites (Oribatida and Gamasina) in short-rotation willow plantings in central New York. *Canadian Journal of Forest Research*, *38*(5), 1061–1070. doi:10.1139/x07-207
363. Minor, M. A., & Norton, R. A. (2004). Effects of soil amendments on assemblages of soil mites (Acari: Oribatida, Mesostigmata) in short-rotation willow plantings in central New York. *Canadian Journal of Forest Research*, *34*(7), 1417–1425. doi:10.1139/x04-035
364. Mirmonsef, H., Hornum, H. D., Jensen, J., & Holmstrup, M. (2017). Effects of an aged copper contamination on distribution of earthworms, reproduction and cocoon hatchability. *Ecotoxicology and Environmental Safety*, *135*, 267–275. doi:10.1016/j.ecoenv.2016.10.012
365. Mirzaei, J. (2016). Impacts of two spatially and temporally isolated anthropogenic fire eventson soils of oak-dominated Zagros forests of Iran. *Turkish Journal of Agriculture and Forestry*, *40*, 109–119. doi:10.3906/tar-1406-61
366. Miura, F., Nakamoto, T., Kaneda, S., Okano, S., Nakajima, M., & Murakami, T. (2008). Dynamics of soil biota at different depths under two contrasting tillage practices. *Soil Biology & Biochemistry*, *40*(2), 406–414. doi:10.1016/j.soilbio.2007.09.004
367. Moldenke, A. R., & Thies, W. G. (1996). Effect on soil arthropods 1 year after application of chloropicrin to control laminated root rot. III Treatment effects on nontarget soil invertebrates. *Canadian Journal of Forest Research*, *26*(1), 120–127. doi:10.1139/x26-013

368. Moos, J. H., Schrader, S., Paulsen, H. M., & Rahmann, G. (2016). Occasional reduced tillage in organic farming can promote earthworm performance and resource efficiency. *Applied Soil Ecology: A Section of Agriculture, Ecosystems & Environment*, *103*, 22–30. doi:10.1016/j.apsoil.2016.01.017
369. Moser T., Van Gestel C. A., Jones S. E., Koolhaas J. E., Rodrigues J. M., Römbke J (2004). Ring-testing and field-validation of a terrestrial model ecosystem (TME)--an instrument for testing potentially harmful substances: effects of carbendazim on enchytraeids. *Ecotoxicology 13* (1-2):89-103. doi: 10.1023/b:ectx.0000012407.42358.3e. PMID: 14992473.
370. Motard, E., Dusz, S., Geslin, B., Akpa-Vinceslas, M., Hignard, C., Babiar, O., … Michel-Salzat, A. (2015). How invasion by Ailanthus altissima transforms soil and litter communities in a temperate forest ecosystem. *Biological Invasions*, *17*(6), 1817–1832. doi:10.1007/s10530-014-0838-3
371. Murchie, A. K., & Gordon, A. W. (2013). The impact of the 'New Zealand flatworm', Arthurdendyus triangulatus, on earthworm populations in the field. *Biological Invasions*, *15*(3), 569–586. doi:10.1007/s10530-012-0309-7
372. Murchie, A. K., Blackshaw, R. P., Gordon, A. W., & Christie, P. (2015). Responses of earthworm species to long-term applications of slurry. *Applied Soil Ecology: A Section of Agriculture, Ecosystems & Environment*, *96*, 60–67. doi:10.1016/j.apsoil.2015.07.005
373. Murphy, W. M., Barreto, A. D. M., Silman, J. P., & Dindal, D. L. (1995). Cattle and sheep grazing effects on soil organisms, fertility and compaction in a smooth-stalked meadowgrass-dominant white clover sward. *Grass and Forage Science: The Journal of the British Grassland Society*, *50*(3), 191–194. doi:10.1111/j.1365-2494.1995.tb02313.x
374. Nagy, P., Bakonyi, G., Bongers, T., Kádár, I., Fábián, M., & Kiss, I. (2004). Effects of microelements on soil nematode assemblages seven years after contaminating an agricultural field. *The Science of the Total Environment*, *320*(2–3), 131–143. doi:10.1016/j.scitotenv.2003.08.006
375. Nagy, D. D., Magura, T., Horváth, R., Debnár, Z., & Tóthmérész, B. (2018). Arthropod assemblages and functional responses along an urbanization gradient: A trait-based multi-taxa approach. *Urban Forestry & Urban Greening*, *30*, 157–168. doi:10.1016/j.ufug.2018.01.002
376. Nahar, M. S., Grewal, P. S., Miller, S. A., Stinner, D., Stinner, B. R., Kleinhenz, M. D., … Doohan, D. (2006). Differential effects of raw and composted manure on nematode community, and its indicative value for soil microbial, physical and chemical properties. *Applied Soil Ecology: A Section of Agriculture, Ecosystems & Environment*, *34*(2–3), 140–151. doi:10.1016/j.apsoil.2006.03.011
377. Nahmani, J., Lavelle, P., Lapied, E., & van Oort, F. (2003). Effects of heavy metal soil pollution on earthworm communities in the north of France. *Pedobiologia*, *47*(5–6), 663–669. doi:10.1078/0031-4056-00243
378. Nakamoto, T., Yamagishi, J., & Miura, F. (2006). Effect of reduced tillage on weeds and soil organisms in winter wheat and summer maize cropping on Humic Andosols in Central Japan. *Soil & Tillage Research*, *85*(1–2), 94–106. doi:10.1016/j.still.2004.12.004
379. Nakamoto, T., & Tsukamoto, M. (2006). Abundance and activity of soil organisms in fields of maize grown with a white clover living mulch. *Agriculture, Ecosystems & Environment*, *115*(1–4), 34–42. doi:10.1016/j.agee.2005.12.006
380. Navarro-Campos, C., Pekas, A., Moraza, M. L., Aguilar, A., & Garcia-Marí, F. (2012). Soil-dwelling predatory mites in citrus: Their potential as natural enemies of thrips with special reference to Pezothrips kellyanus (Thysanoptera: Thripidae). *Biological Control: Theory and Applications in Pest Management*, *63*(2), 201–209. doi:10.1016/j.biocontrol.2012.07.007
381. Negrete-Yankelevich, S., Fragoso, C., Newton, A. C., & Heal, O. W. (2007). Successional changes in soil, litter and macroinvertebrate parameters following selective logging in a Mexican Cloud

Forest. *Applied Soil Ecology: A Section of Agriculture, Ecosystems & Environment*, *35*(2), 340–355. doi:10.1016/j.apsoil.2006.07.006

382. Neher, D. A., Wu, J., Barbercheck, M. E., & Anas, O. (2005). Ecosystem type affects interpretation of soil nematode community measures. *Applied Soil Ecology: A Section of Agriculture, Ecosystems & Environment*, *30*(1), 47–64. doi:10.1016/j.apsoil.2005.01.002

383. Neher, D. A., Weicht, T. R., Moorhead, D. L., & Sinsabaugh, R. L. (2004). Elevated CO2 alters functional attributes of nematode communities in forest soils. *Functional Ecology*, *18*(4), 584–591. doi:10.1111/j.0269-8463.2004.00866.x

384. Neher D. A., & Weicht T. R. (2013). Nematode Genera in Forest Soil Respond Differentially to Elevated CO2. *The Journal of Nematology, 45*(3), 214-22

385. Neher, D. A., & Olson, R.K. (1999). Nematode communities in soils of four farm cropping management systems*. Pedobiologia, 43*, 430-438.

386. Nesbitt, J. E., & Adl, S. M. (2014). Differences in soil quality indicators between organic and sustainably managed potato fields in Eastern Canada. *Ecological Indicators*, *37*, 119–130. doi:10.1016/j.ecolind.2013.10.002

387. Ngosong, C., Raupp, J., Scheu, S., & Ruess, L. (2009). Low importance for a fungal based food web in arable soils under mineral and organic fertilization indicated by Collembola grazers. *Soil Biology & Biochemistry*, *41*(11), 2308–2317. doi:10.1016/j.soilbio.2009.08.015

388. Nieminen, J. K., Räisänen, M., & Haimi, J. (2012). Spot mounding and granulated wood ash increase inorganic N availability and alter key components of the soil food web in clear-cut Norway spruce forests. *Forest Ecology and Management*, *263*, 24–30. doi:10.1016/j.foreco.2011.09.023

389. Nieminen, J. K., & Räisänen, M. (2013). Effects of sewage sludge addition to Norway spruce seedlings on nitrogen availability and soil fauna in clear-cut areas. *Environmental Pollution*, *178*, 306–311. doi:10.1016/j.envpol.2013.03.045

390. Nieminen, J. K., & Pohjola, P. (2014). Labile carbon addition affects soil organisms and N availability but not cellulose decomposition in clear-cut Norway spruce forests. *Boreal Environment Research, 19*, 257-266.

391. Niklaus, P. A., Alphei, J., Kampichler, C., Kandeler, E., Körner, C., Tscherko, D., & Wohlfender, M. (2007). Interactive effects of plant species diversity and elevated CO2 on soil biota and nutrient cycling. *Ecology*, *88*(12), 3153–3163. doi:10.1890/06-2100.1

392. Niklaus, P. A., Alphei, J., Ebersberger, D., Kampichler, C., Kandeler, E., & Tscherko, D. (2003). Six years of in situ CO2 enrichment evoke changes in soil structure and soil biota of nutrient-poor grassland. *Global Change Biology*, *9*(4), 585–600. doi:10.1046/j.1365-2486.2003.00614.x

393. Norris, S. L., Blackshaw, R. P., Dunn, R. M., Critchley, N. R., Smith, K. E., Williams, J. R., … Murray, P. J. (2016). Improving above and below-ground arthropod biodiversity in maize cultivation systems. *Applied Soil Ecology: A Section of Agriculture, Ecosystems & Environment*, *108*, 25–46. doi:10.1016/j.apsoil.2016.07.015

394. Ochoa-Hueso, R., Rocha, I., Stevens, C. J., Manrique, E., & Luciañez, M. J. (2014). Simulated nitrogen deposition affects soil fauna from a semiarid Mediterranean ecosystem in central Spain. *Biology and Fertility of Soils*, *50*(1), 191–196. doi:10.1007/s00374-013-0838-y

395. Olejniczak, I., & Lenart, S. (2017). A comparison of tillage, direct drilling and lime on springtail communities in a long-term field trial in poland. *Israel Journal of Ecology & Evolution*, *63*(2), 17–24. doi:10.1163/22244662-06301004

396. Oliver, I., Garden, D., Greenslade, P. J., Haller, B., Rodgers, D., Seeman, O., & Johnston, B. (2005). Effects of fertiliser and grazing on the arthropod communities of a native grassland in south-

eastern Australia. *Agriculture, Ecosystems & Environment*, *109*(3–4), 323–334. doi:10.1016/j.agee.2005.02.022

397. Osler, G. H. R., Westhorpe, D., & Oliver, I. (2001). The short-term effects of endosulfan discharges on eucalypt floodplain soil microarthropods. *Applied Soil Ecology: A Section of Agriculture, Ecosystems & Environment*, *16*(3), 263–273. doi:10.1016/s0929-1393(00)00127-x

398. Osuji, L. C., Adesiyan, S. O., & Obute, G. C. (2004). Post-impact assessment of oil pollution in Agbada west plain of Niger Delta, Nigeria: field reconnaissance and total extractable hydrocarbon content. *Chemistry & Biodiversity*, *1*(10), 1569–1578. doi:10.1002/cbdv.200490117

399. Overstreet, L. F., Hoyt, G. D., & Imbriani, J. (2010). Comparing nematode and earthworm communities under combinations of conventional and conservation vegetable production practices. *Soil & Tillage Research*, *110*(1), 42–50. doi:10.1016/j.still.2010.06.009

400. Page, H. M., Lastra, M., Rodil, I. F., Briones, M. J. I., & Garrido, J. (2010). Effects of non-native Spartina patens on plant and sediment organic matter carbon incorporation into the local invertebrate community. *Biological Invasions*, *12*(11), 3825–3838. doi:10.1007/s10530-010-9775-y

401. Pan, F., Han, X., McLaughlin, N. B., Han, X., Li, C., Zhao, D., … Xu, Y. (2015). Effect of long-term fertilization on free-living nematode community structure in Mollisols. *Journal of Soil Science and Plant Nutrition*, (ahead), 0–0. doi:10.4067/s0718-95162015005000011

402. Pan, Fengjuan, Xu, Y., McLaughlin, N. B., Liu, W., Han, X., Zhan, L., & Zhao, D. (2013). Influence of different fertilization regimes on plant-parasitic nematodes in the black soil region of Northeast China. *Phytoparasitica; Israel Journal of Plant Protection Sciences*, *41*(4), 355–363. doi:10.1007/s12600-013-0295-z

403. Pan, Fengjuan, McLaughlin, N. B., Yu, Q., Xue, A. G., Xu, Y., Han, X., … Zhao, D. (2010). Responses of soil nematode community structure to different long-term fertilizer strategies in the soybean phase of a soybean–wheat–corn rotation. *European Journal of Soil Biology*, *46*(2), 105–111. doi:10.1016/j.ejsobi.2010.01.004

404. Pan, Fengjuan, Li, N., Zou, W., Han, X., & McLaughlin, N. B. (2016). Soil nematode community structure and metabolic footprint in the early pedogenesis of a Mollisol. *European Journal of Soil Biology*, *77*, 17–25. doi:10.1016/j.ejsobi.2016.09.004

405. Panesar, T. S., Marshall, V. G., & Barclay, H. J. (2000). The impact of clearcutting and partial harvesting systems on population dynamics of soil nematodes in coastal Douglas-fir forests. *Pedobiologia*, *44*(6), 641–665. doi:10.1078/s0031-4056(04)70079-7

406. Paoletti, M. G., Schweigl, U., & Favretto, M. R., (1995). Soil macroinvertebrates, heavy metals and organochlorines in low and high input apple orchards and coppiced woodland. *Pedobiologia, 39*(1), 20-33.

407. Papatheodorou, E. M., Kordatos, H., Kouseras, T., Monokrousos, N., Menkissoglu-Spiroudi, U., Diamantopoulos, J., … Argyropoulou, M. D. (2012). Differential responses of structural and functional aspects of soil microbes and nematodes to abiotic and biotic modifications of the soil environment. *Applied Soil Ecology: A Section of Agriculture, Ecosystems & Environment*, *61*, 26–33. doi:10.1016/j.apsoil.2012.04.002

408. Park, B.-Y., Lee, J.-K., Ro, H.-M., & Kim, Y. H. (2016). Short-term effects of low-level heavy metal contamination on soil health analyzed by nematode community structure. *The Plant Pathology Journal*, *32*(4), 329–339. doi:10.5423/PPJ.OA.12.2015.0272

409. Park, B.-Y., Lee, J.-K., Ro, H.-M., & Kim, Y. H. (2011). Effects of heavy metal contamination from an abandoned mine on nematode community structure as an indicator of soil ecosystem health. *Applied Soil Ecology: A Section of Agriculture, Ecosystems & Environment*, *51*, 17–24. doi:10.1016/j.apsoil.2011.08.006

410. Parmelee, R. W., Wentsel, R. S., Phillips, C. T., Checkai, R. T. & Simini, M. (1993). Soil microcosm for testing the effects of chemical pollutants on soil fauna communities and trophic structure. *Environmental Toxicology and Chemistry, 12*, 1477-1486. doi:10.1002/etc.5620120815
411. Paterson, E., Neilson, R., Midwood, A. J., Osborne, S. M., Sim, A., Thornton, B., & Millard, P. (2011). Altered food web structure and C-flux pathways associated with mineralisation of organic amendments to agricultural soil. *Applied Soil Ecology: A Section of Agriculture, Ecosystems & Environment*, *48*(2), 107–116. doi:10.1016/j.apsoil.2011.04.006
412. Pattison, A. B., Moody, P. W., Badcock, K. A., Smith, L. J., Armour, J. A., Rasiah, V., … Mayer, R. (2008). Development of key soil health indicators for the Australian banana industry. *Applied Soil Ecology: A Section of Agriculture, Ecosystems & Environment*, *40*(1), 155–164. doi:10.1016/j.apsoil.2008.04.002
413. Paul, B. K., Vanlauwe, B., Hoogmoed, M., Hurisso, T. T., Ndabamenye, T., Terano, Y., … Pulleman, M. M. (2015). Exclusion of soil macrofauna did not affect soil quality but increased crop yields in a sub-humid tropical maize-based system. *Agriculture, Ecosystems & Environment*, *208*, 75–85. doi:10.1016/j.agee.2015.04.001
414. Pavao-Zuckerman, M. A., & Sookhdeo, C. (2017). Nematode community response to green infrastructure design in a semiarid city. *Journal of Environmental Quality*, *46*(3), 687–694. doi:10.2134/jeq2016.11.0461
415. Peck, R. W., & Niwa, C. G. (2005). Longer-term effects of selective thinning on microarthropod communities in a late-successional coniferous forest. *Environmental Entomology*, *34*(3), 646–655. doi:10.1603/0046-225x-34.3.646
416. Peck, D. C. (2009). Long-term effects of imidacloprid on the abundance of surface- and soil-active nontarget fauna in turf. *Agricultural and Forest Entomology*, *11*(4), 405–419. doi:10.1111/j.1461-9563.2009.00454.x
417. Pelosi, C., Pey, B., Hedde, M., Caro, G., Capowiez, Y., Guernion, M., … Cluzeau, D. (2014). Reducing tillage in cultivated fields increases earthworm functional diversity. *Applied Soil Ecology: A Section of Agriculture, Ecosystems & Environment*, *83*, 79–87. doi:10.1016/j.apsoil.2013.10.005
418. Pelosi, Céline, Bertrand, M., Thénard, J., & Mougin, C. (2015). Earthworms in a 15 years agricultural trial. *Applied Soil Ecology: A Section of Agriculture, Ecosystems & Environment*, *88*, 1–8. doi:10.1016/j.apsoil.2014.12.004
419. Pen-Mouratov, S., Shukurov, N., Yu, J., Rakhmonkulova, S., Kodirov, O., Barness, G., … Steinberger, Y. (2014). Successive development of soil ecosystems at abandoned coal-ash landfills. *Ecotoxicology*, *23*(5), 880–897. doi:10.1007/s10646-014-1227-5
420. Pen-Mouratov, S., Shukurov, N., & Steinberger, Y. (2008). Influence of industrial heavy metal pollution on soil free-living nematode population. *Environmental*, *152*(1), 172–183. doi:10.1016/j.envpol.2007.05.007
421. Perdue, J. C., & Crossley, D. A., Jr. (1989). Seasonal abundance of soil mites (Acari) in experimental agroecosystems: Effects of drought in no-tillage and conventional tillage. *Soil & Tillage Research*, *15*(1–2), 117–124. doi:10.1016/0167-1987(89)90068-8
422. Petersen, H., Jucevica, E., & Gjelstrup, P. (2004). Long-term changes in collembolan communities in grazed and non-grazed abandoned arable fields in Denmark. *Pedobiologia*, *48*(5–6), 559–573. doi:10.1016/j.pedobi.2004.06.003
423. Pfiffner, L., & Luka, H. (2007). Earthworm populations in two low-input cereal farming systems. *Applied Soil Ecology: A Section of Agriculture, Ecosystems & Environment*, *37*(3), 184–191. doi:10.1016/j.apsoil.2007.06.005

424. Pižl, V., & Schlaghamerský, J. (2007). The impact of pedestrian activity on soil annelids in urban greens. *European Journal of Soil Biology*, *43*, S68–S71. doi:10.1016/j.ejsobi.2007.08.004

425. Pizl, V., & Josens, G. (1995). Earthworm communities along a gradient of urbanization. *Environmental Pollution*, *90*(1), 7–14. doi:10.1016/0269-7491(94)00097-w

426. Pokharel, R., Marahatta, S. P., Handoo, Z. A., & Chitwood, D. J. (2015). Nematode community structures in different deciduous tree fruits and grape in Colorado, USA and impact of organic peach and apple production practices. *European Journal of Soil Biology*, *67*, 59–68. doi:10.1016/j.ejsobi.2015.02.003

427. Ponge, J.-F., Bandyopadhyaya, I., & Marchetti, V. (2002). Interaction between humus form and herbicide toxicity to Collembola (Hexapoda). *Applied Soil Ecology: A Section of Agriculture, Ecosystems & Environment*, *20*(3), 239–253. doi:10.1016/s0929-1393(02)00026-4

428. Porazinska D. L., Pratt P. D., & Glblin-Davis R. M. (2007). Consequences of Melaleuca quinquenervia invasion on soil nematodes in the Florida Everglades. *The Journal of Nematology, 39*(4), 305-12.

429. Prendergast-Miller, M., Cole, L., Standen, V., Rees, R., Parker, J., Leith, I., & Sheppard, L. (2008). Are enchytraeid worms (Oligochaeta) sensitive indicators of ammonia-N impacts on an ombrotrophic bog? *European Journal of Soil Biology*, *44*(1), 101–108. doi:10.1016/j.ejsobi.2007.08.039

430. Pressler, Y., Foster, E. J., Moore, J. C., & Cotrufo, M. F. (2017). Coupled biochar amendment and limited irrigation strategies do not affect a degraded soil food web in a maize agroecosystem, compared to the native grassland. *Global Change Biology. Bioenergy*, *9*(8), 1344–1355. doi:10.1111/gcbb.12429

431. Qi, S., Zheng, H., Lin, Q., Li, G., Xi, Z., & Zhao, X. (2011). Effects of livestock grazing intensity on soil biota in a semiarid steppe of Inner Mongolia. *Plant and Soil*, *340*(1–2), 117–126. doi:10.1007/s11104-010-0463-6

432. Quist, C. W., Vervoort, M. T. W., Van Megen, H., Gort, G., Bakker, J., Van der Putten, W. H., & Helder, J. (2014). Selective alteration of soil food web components by invasive giant goldenrodSolidago giganteain two distinct habitat types. *Oikos (Copenhagen, Denmark)*, *123*(7), 837–845. doi:10.1111/oik.01067

433. Quist, C. W., Schrama, M., de Haan, J. J., Smant, G., Bakker, J., van der Putten, W. H., & Helder, J. (2016). Organic farming practices result in compositional shifts in nematode communities that exceed crop-related changes. *Applied Soil Ecology: A Section of Agriculture, Ecosystems & Environment*, *98*, 254–260. doi:10.1016/j.apsoil.2015.10.022

434. Rantalainen, M.-L., Torkkeli, M., Strömmer, R., & Setälä, H. (2006). Lead contamination of an old shooting range affecting the local ecosystem--A case study with a holistic approach. *The Science of the Total Environment*, *369*(1–3), 99–108. doi:10.1016/j.scitotenv.2006.05.005

435. Rantalainen, M.-L., Kontiola, L., Haimi, J., Fritze, H., & Setälä, H. (2004). Influence of resource quality on the composition of soil decomposer community in fragmented and continuous habitat. *Soil Biology & Biochemistry*, *36*(12), 1983–1996. doi:10.1016/j.soilbio.2004.05.017

436. Rantalainen, M.-L., Haimi, J., & Setälä, H. (2004). Testing the usefulness of habitat corridors in mitigating the negative effects of fragmentation: the soil faunal community as a model system. *Applied Soil Ecology: A Section of Agriculture, Ecosystems & Environment*, *25*(3), 267–274. doi:10.1016/j.apsoil.2003.09.004

437. Rantalainen, M.-L., Haimi, J., Fritze, H., & Setälä, H. (2006). Effects of small-scale habitat fragmentation, habitat corridors and mainland dispersal on soil decomposer organisms. *Applied Soil Ecology: A Section of Agriculture, Ecosystems & Environment*, *34*(2–3), 152–159. doi:10.1016/j.apsoil.2006.03.004

438. Rantalainen, M.-L., Fritze, H., Haimi, J., Pennanen, T., & Setälä, H. (2005). Colonisation of newly established habitats by soil decomposer organisms: the effect of habitat corridors in relation to colonisation distance and habitat size. *Applied Soil Ecology: A Section of Agriculture, Ecosystems & Environment*, *28*(1), 67–77. doi:10.1016/j.apsoil.2004.06.004

439. Rantalainen, M.-L., Fritze, H., Haimi, J., Pennanen, T. & Setälä, H. (2005). Species richness and food web structure of soil decomposer community as affected by the size of habitat fragment and habitat corridors. *Global Change Biology, 11*: 1614-1627. https://doi.org/10.1111/j.1365-2486.2005.000999.x

440. Rashid, M. I., de Goede, R. G. M., Corral Nunez, G. A., Brussaard, L., & Lantinga, E. A. (2014). Soil pH and earthworms affect herbage nitrogen recovery from solid cattle manure in production grassland. *Soil Biology & Biochemistry*, *68*, 1–8. doi:10.1016/j.soilbio.2013.09.013

441. Reid, M. L., & Emery, S. M. (2018). Scale-dependent effects of Gypsophila paniculata invasion and management on plant and soil nematode community diversity and heterogeneity. *Biological Conservation*, *224*, 153–161. doi:10.1016/j.biocon.2018.05.026

442. Reilly, K., Cullen, E., Lola-Luz, T., Stone, D., Valverde, J., Gaffney, M., … Griffiths, B. S. (2013). Effect of organic, conventional and mixed cultivation practices on soil microbial community structure and nematode abundance in a cultivated onion crop. *Journal of the Science of Food and Agriculture*, *93*(15), 3700–3709. doi:10.1002/jsfa.6206

443. Renčo, M., & Baležentiené, L. (2015). An analysis of soil free-living and plant-parasitic nematode communities in three habitats invaded by Heracleum sosnowskyi in central Lithuania. *Biological Invasions*, *17*(4), 1025–1039. doi:10.1007/s10530-014-0773-3

444. Renčo, M., Čerevková, A., Homolová, Z., & Gömöryová, E. (2015). Long-term effects on soil nematode community structure in spruce forests of removing or not removing fallen trees after a windstorm. *Forest Ecology and Management*, *356*, 243–252. doi:10.1016/j.foreco.2015.07.008

445. Rieske, L. K., & Buss, L. J. (2001). Effects of gypsy moth suppression tactics on litter- and ground-dwelling arthropods in the central hardwood forests of the Cumberland Plateau. *Forest Ecology and Management*, *149*(1–3), 181–195. doi:10.1016/s0378-1127(00)00552-1

446. Rillig, M. C., Field, C. B., & Allen, M. F. (1999). Soil biota responses to long-term atmospheric CO2 enrichment in two California annual grasslands. *Oecologia*, *119*(4), 572–577. doi:10.1007/s004420050821

447. Roarty, S., & Schmidt, O. (2013). Permanent and new arable field margins support large earthworm communities but do not increase in-field populations. *Agriculture, Ecosystems & Environment*, *170*, 45–55. doi:10.1016/j.agee.2013.02.011

448. Robson, T., Pancotto, V., Scopel, A., Flint, S., & Caldwell, M. (2005). Solar UV-B influences microfaunal community composition in a Tierra del Fuego peatland. *Soil Biology & Biochemistry*, *37*(12), 2205–2215. doi:10.1016/j.soilbio.2005.04.002

449. Römbke, J., Coors, A., Fernández, Á. A., Förster, B., Fernández, C., Jensen, J., … Liebig, M. (2010). Effects of the parasiticide ivermectin on the structure and function of dung and soil invertebrate communities in the field (Madrid, Spain). *Applied Soil Ecology: A Section of Agriculture, Ecosystems & Environment*, *45*(3), 284–292. doi:10.1016/j.apsoil.2010.05.004

450. Rota, E., Caruso, T., & Bargagli, R. (2014). Community structure, diversity and spatial organization of enchytraeids in Mediterranean urban holm oak stands. *European Journal of Soil Biology*, *62*, 83–91. doi:10.1016/j.ejsobi.2014.02.016

451. Roth, E., Samara, N., Ackermann, M., Seiml-Buchinger, R., Saleh, A., & Ruess, L. (2015). Fertilization and irrigation practice as source of microorganisms and the impact on nematodes as their

potential vectors. *Applied Soil Ecology: A Section of Agriculture, Ecosystems & Environment*, *90*, 68–77. doi:10.1016/j.apsoil.2015.02.002
452. Rowles, A. D., & O'dowd, D. J. (2009). Impacts of the invasive Argentine ant on native ants and other invertebrates in coastal scrub in south-eastern Australia. *Austral Ecology*, *34*(3), 239–248. doi:10.1111/j.1442-9993.2008.01922.x
453. Ruess, L., Michelsen, A., Schmidt, I. K., & Jonasson, S. (1999). Simulated climate change affecting microorganisms, nematode density and biodiversity in subarctic soils. *Plant and Soil 212,* 63–73. https://doi.org/10.1023/A:1004567816355
454. Rzeszowski, K., & Sterzyńska, M. (2016). Changes through time in soil Collembola communities exposed to urbanization. *Urban Ecosystems*, *19*(1), 143–158. doi:10.1007/s11252-015-0478-0
455. Sørensen, L. I., Mikola, J., Kytöviita, M.-M., & Olofsson, J. (2009). Trampling and spatial heterogeneity explain decomposer abundances in a sub-arctic grassland subjected to simulated reindeer grazing. *Ecosystems (New York, N.Y.)*, *12*(5), 830–842. doi:10.1007/s10021-009-9260-6
456. Sądej, W., Sądej, W., Rozmysłowicz (2008). Soil concentration of C and N shaped by long-term unidirectional fertilization versus noxious soil macrofauna. *Journal of Elementology, 13*(3), 381-389.
457. Šalamún, P., Renčo, M., Miklisová, D., & Hanzelová, V. (2011). Nematode community structure in the vicinity of a metallurgical factory. *Environmental Monitoring and Assessment*, *183*(1–4), 451–464. doi:10.1007/s10661-011-1932-y
458. Šalamún, P., Renčo, M., Kucanová, E., Brázová, T., Papajová, I., Miklisová, D., & Hanzelová, V. (2012). Nematodes as bioindicators of soil degradation due to heavy metals. *Ecotoxicology*, *21*(8), 2319–2330. doi:10.1007/s10646-012-0988-y
459. Šalamún, P., Kucanová, E., Brázová, T., Miklisová, D., Renčo, M., & Hanzelová, V. (2014). Diversity and food web structure of nematode communities under high soil salinity and alkaline pH. *Ecotoxicology*, *23*(8), 1367–1376. doi:10.1007/s10646-014-1278-7
460. Šalamún, P., Hanzelová, V., Miklisová, D., Šestinová, O., Findoráková, L., & Kováčik, P. (2017). The effects of vegetation cover on soil nematode communities in various biotopes disturbed by industrial emissions. *The Science of the Total Environment*, *592*, 106–114. doi:10.1016/j.scitotenv.2017.02.238
461. Šalamún, P., Hanzelová, V., Miklisová, D., & Brázová, T. (2015). Effect of heavy metals on soil nematode communities in the vicinity of a metallurgical plant in North Slovakia. *Helminthologia*, *52*(3), 252–260. doi:10.1515/helmin-2015-0040
462. Šalamún, P., Brázová, T., Miklisová, D., & Hanzelová, V. (2015). Influence of selected heavy metals (As, Cd, Cr, Cu) on nematode communities in experimental soil microcosm. *Helminthologia*, *52*(4), 341–347. doi:10.1515/helmin-2015-0054
463. Salminen, J., & Haimi, J. (1998). Responses of the soil decomposer community and decomposition processes to the combined stress of pentachlorophenol and acid precipitation. *Applied Soil Ecology: A Section of Agriculture, Ecosystems & Environment*, *9*(1–3), 475–481. doi:10.1016/s0929-1393(98)00108-5
464. Salminen, J. E., & Sulkava, P. O. (1997). Decomposer communities in contaminated soil: Is altered community regulation a proper tool in ecological risk assessment of toxicants? *Environmental Pollution*, *97*(1–2), 45–53. doi:10.1016/s0269-7491(97)00078-x
465. Sánchez-de León, Y., Wise, D. H., Lugo-Pérez, J., Norby, R. J., James, S. W., & Gonzalez-Meler, M. A. (2018). Endogeic earthworm densities increase in response to higher fine-root production in a forest exposed to elevated CO2. *Soil Biology & Biochemistry*, *122*, 31–38. doi:10.1016/j.soilbio.2018.03.027

466. Sánchez-Moreno, S., & Navas, A. (2007). Nematode diversity and food web condition in heavy metal polluted soils in a river basin in southern Spain. *European Journal of Soil Biology*, *43*(3), 166–179. doi:10.1016/j.ejsobi.2007.01.002
467. Sánchez-Moreno, S., Minoshima, H., Ferris, H., & Jackson, L. E. (2006). Linking soil properties and nematode community composition: effects of soil management on soil food webs. *Nematology: International Journal of Fundamental and Applied Nematological Research*, *8*(5), 703–715. doi:10.1163/156854106778877857
468. Sánchez-Moreno, S., Jiménez, L., Alonso-Prados, J. L., & García-Baudín, J. M. (2010). Nematodes as indicators of fumigant effects on soil food webs in strawberry crops in Southern Spain. *Ecological Indicators*, *10*(2), 148–156. doi:10.1016/j.ecolind.2009.04.010
469. Sánchez-Moreno, S., Castro, J., Alonso-Prados, E., Alonso-Prados, J. L., García-Baudín, J. M., Talavera, M., & Durán-Zuazo, V. H. (2015). Tillage and herbicide decrease soil biodiversity in olive orchards. *Agronomy for Sustainable Development*, *35*(2), 691–700. doi:10.1007/s13593-014-0266-x
470. Santonja, M., Fernandez, C., Proffit, M., Gers, C., Gauquelin, T., Reiter, I. M., … Baldy, V. (2017). Plant litter mixture partly mitigates the negative effects of extended drought on soil biota and litter decomposition in a Mediterranean oak forest. *The Journal of Ecology*, *105*(3), 801–815. doi:10.1111/1365-2745.12711
471. Santorufo, L., Van Gestel, C. A. M., Rocco, A., & Maisto, G. (2012). Soil invertebrates as bioindicators of urban soil quality. *Environmental Pollution*, *161*, 57–63. doi:10.1016/j.envpol.2011.09.042
472. Santorufo, L., Van Gestel, C. A. M., & Maisto, G. (2014). Sampling season affects conclusions on soil arthropod community structure responses to metal pollution in Mediterranean urban soils. *Geoderma*, *226–227*, 47–53. doi:10.1016/j.geoderma.2014.02.001
473. Santorufo, L., Cortet, J., Arena, C., Goudon, R., Rakoto, A., Morel, J.-L., & Maisto, G. (2014). An assessment of the influence of the urban environment on collembolan communities in soils using taxonomy- and trait-based approaches. *Applied Soil Ecology: A Section of Agriculture, Ecosystems & Environment*, *78*, 48–56. doi:10.1016/j.apsoil.2014.02.008
474. Sauvadet, M., Chauvat, M., Fanin, N., Coulibaly, S., & Bertrand, I. (2016). Comparing the effects of litter quantity and quality on soil biota structure and functioning: Application to a cultivated soil in Northern France. *Applied Soil Ecology: A Section of Agriculture, Ecosystems & Environment*, *107*, 261–271. doi:10.1016/j.apsoil.2016.06.010
475. Scheffczyk, A., Floate, K. D., Blanckenhorn, W. U., Düring, R.-A., Klockner, A., Lahr, J., … Römbke, J. (2016). Nontarget effects of ivermectin residues on earthworms and springtails dwelling beneath dung of treated cattle in four countries. *Environmental Toxicology and Chemistry*, *35*(8), 1959–1969. doi:10.1002/etc.3306
476. Scheu, S. & Schaefer, M. (1998). Bottom-up control of the soil macrofauna community in a beechwood on limestone: Manipulation of food resources. *Ecology, 79*, 1573-1585. doi:10.1890/0012-9658(1998)079[1573:BUCOTS]2.0.CO;2
477. Scheunemann, N., Maraun, M., Scheu, S., & Butenschoen, O. (2015). The role of shoot residues vs. crop species for soil arthropod diversity and abundance of arable systems. *Soil Biology & Biochemistry*, *81*, 81–88. doi:10.1016/j.soilbio.2014.11.006
478. Schlaghamerský, J., Šídová, A., & Pižl, V. (2007). From mowing to grazing: Does the change in grassland management affect soil annelid assemblages? *European Journal of Soil Biology*, *43*, S72–S78. doi:10.1016/j.ejsobi.2007.08.054
479. Schlaghamerský, J., Eisenhauer, N., & Frelich, L. E. (2014). Earthworm invasion alters enchytraeid community composition and individual biomass in northern hardwood forests of North

America. *Applied Soil Ecology: A Section of Agriculture, Ecosystems & Environment*, *83*, 159–169. doi:10.1016/j.apsoil.2013.09.005

480. Schmidt, O., Clements, R. O., & Donaldson, G. (2003). Why do cereal–legume intercrops support large earthworm populations? *Applied Soil Ecology: A Section of Agriculture, Ecosystems & Environment*, *22*(2), 181–190. doi:10.1016/s0929-1393(02)00131-2

481. Schmidt, I. K., Ruess, L., Bååth, E., Michelsen, A., Ekelund, F., & Jonasson, S. (2000). Long-term manipulation of the microbes and microfauna of two subarctic heaths by addition of fungicide, bactericide, carbon and fertilizer. *Soil Biology & Biochemistry*, *32*(5), 707–720. doi:10.1016/s0038-0717(99)00207-2

482. Schon, N. L., Mackay, A. D., Yeates, G. W., & Minor, M. A. (2010). Separating the effects of defoliation and dairy cow treading pressure on the abundance and diversity of soil invertebrates in pastures. *Applied Soil Ecology: A Section of Agriculture, Ecosystems & Environment*, *46*(2), 209–221. doi:10.1016/j.apsoil.2010.08.011

483. Schon, N. L., Mackay, A. D., Minor, M. A., Yeates, G. W., & Hedley, M. J. (2008). Soil fauna in grazed New Zealand hill country pastures at two management intensities. *Applied Soil Ecology: A Section of Agriculture, Ecosystems & Environment*, *40*(2), 218–228. doi:10.1016/j.apsoil.2008.04.007

484. Schon, N. L., Mackay, A. D., & Minor, M. A. (2011). Soil fauna in sheep-grazed hill pastures under organic and conventional livestock management and in an adjacent ungrazed pasture. *Pedobiologia*, *54*(3), 161–168. doi:10.1016/j.pedobi.2011.01.001

485. Schon, N. L., Dennis, S., Fraser, P. M., White, T. A., & Knight, T. L. (2017). Intensification of pastoral systems influences earthworm populations. *New Zealand Journal of Agricultural Research*, *60*(4), 423–436. doi:10.1080/00288233.2017.1363788

486. Schrader, S., Bender, J., & Weigel, H.-J. (2009). Ozone exposure of field-grown winter wheat affects soil mesofauna in the rhizosphere. *Environmental Pollution*, *157*(12), 3357–3362. doi:10.1016/j.envpol.2009.06.031

487. Selonen, S., Liiri, M., & Setälä, H. (2014). Can the soil fauna of boreal forests recover from lead-derived stress in a shooting range area? *Ecotoxicology*, *23*(3), 437–448. doi:10.1007/s10646-014-1210-1

488. Sepp, K., Ivask, M., Kaasik, A., Mikk, M., & Peepson, A. (2005). Soil biota indicators for monitoring the Estonian agri-environmental programme. *Agriculture, Ecosystems & Environment*, *108*(3), 264–273. doi:10.1016/j.agee.2005.02.007

489. Shao, Y., Zhang, W., Shen, J., Zhou, L., Xia, H., Shu, W., … Fu, S. (2008). Nematodes as indicators of soil recovery in tailings of a lead/zinc mine. *Soil Biology & Biochemistry*, *40*(8), 2040–2046. doi:10.1016/j.soilbio.2008.04.014

490. Shao, Y., Zhang, W., Liu, Z., Sun, Y., Chen, D., Wu, J., … Fu, S. (2012). Responses of soil microbial and nematode communities to aluminum toxicity in vegetated oil-shale-waste lands. *Ecotoxicology*, *21*(8), 2132–2142. doi:10.1007/s10646-012-0966-4

491. Shao, Y., Wang, X., Zhao, J., Wu, J., Zhang, W., Neher, D. A., … Fu, S. (2016). Subordinate plants sustain the complexity and stability of soil micro-food webs in natural bamboo forest ecosystems. *The Journal of Applied Ecology*, *53*(1), 130–139. doi:10.1111/1365-2664.12538

492. Shelton, A. L., Henning, J. A., Schultz, P., & Clay, K. (2014). Effects of abundant white-tailed deer on vegetation, animals, mycorrhizal fungi, and soils. *Forest Ecology and Management*, *320*, 39–49. doi:10.1016/j.foreco.2014.02.026

493. Shukurov, N., Kodirov, O., Peitzsch, M., Kersten, M., Pen-Mouratov, S., & Steinberger, Y. (2014). Coupling geochemical, mineralogical and microbiological approaches to assess the health of

contaminated soil around the Almalyk mining and smelter complex, Uzbekistan. *The Science of the Total Environment*, *476–477*, 447–459. doi:10.1016/j.scitotenv.2014.01.031
494. Siepel, H., Vogels, J., Bobbink, R., Bijlsma, R.-J., Jongejans, E., de Waal, R., & Weijters, M. (2018). Continuous and cumulative acidification and N deposition induce P limitation of the micro-arthropod soil fauna of mineral-poor dry heathlands. *Soil Biology & Biochemistry*, *119*, 128–134. doi:10.1016/j.soilbio.2018.01.025
495. Siepel, H. (1996). Biodiversity of soil microarthropods: the filtering of species. *Biodiversity and Conservation*, *5*(2), 251–260. doi:10.1007/bf00055834
496. Siira-Pietikäinen, A., Pietikäinen, J., Fritze, H., & Haimi, J. (2001). Short-term responses of soil decomposer communities to forest management: clear felling versus alternative forest harvesting methods. *Canadian Journal of Forest Research*, *31*(1), 88–99. doi:10.1139/cjfr-31-1-88
497. Siira-Pietikäinen, A., Haimi, J., Kanninen, A., Pietikäinen, J., & Fritze, H. (2001). Responses of decomposer community to root-isolation and addition of slash. *Soil Biology & Biochemistry*, *33*(14), 1993–2004. doi:10.1016/s0038-0717(01)00135-3
498. Silva, E., Santos, A., Korasaki, V., Evangelista, A., Bignell, D., Constantino, R., & Zanetti, R. (2016). Does fipronil application on roots affect the structure of termite communities in eucalypt plantations? *Forest Ecology and Management*, *377*, 55–60. doi:10.1016/j.foreco.2016.06.035
499. Simmons, B. L., Wall, D. H., Adams, B. J., Ayres, E., Barrett, J. E., & Virginia, R. A. (2009). Terrestrial mesofauna in above- and below-ground habitats: Taylor Valley, Antarctica. *Polar Biology*, *32*(11), 1549–1558. doi:10.1007/s00300-009-0639-9
500. Simoni, S., Gagnarli, E., Goggioli, D., Guidi, S., Tarchi, F., D'Avino, L., & Giffard, B. (2018). *Impact of agricultural management on communities of Oribatida, Gamasina and Collembola in Italian and French vineyards*. doi:10.6092/ISSN.2281-4485/7902
501. Sithole, N. J., Magwaza, L. S., Mafongoya, P. L., & Thibaud, G. R. (2018). Long-term impact of no-till conservation agriculture on abundance and order diversity of soil macrofauna in continuous maize monocropping system. *Acta Agriculturae Scandinavica. Section B, Soil and Plant Science*, *68*(3), 220–229. doi:10.1080/09064710.2017.1381276
502. Sizmur, T., Martin, E., Wagner, K., Parmentier, E., Watts, C., & Whitmore, A. P. (2017). Milled cereal straw accelerates earthworm (Lumbricus terrestris) growth more than selected organic amendments. *Applied Soil Ecology: A Section of Agriculture, Ecosystems & Environment*, *113*, 166–177. doi:10.1016/j.apsoil.2016.12.006
503. Sjursen, H., Michelsen, A., & Jonasson, S. (2005). Effects of long-term soil warming and fertilisation on microarthropod abundances in three sub-arctic ecosystems. *Applied Soil Ecology: A Section of Agriculture, Ecosystems & Environment*, *30*(3), 148–161. doi:10.1016/j.apsoil.2005.02.013
504. Skubała, P., & Zaleski, T. (2012). Heavy metal sensitivity and bioconcentration in oribatid mites (Acari, Oribatida) Gradient study in meadow ecosystems. *The Science of the Total Environment*, *414*, 364–372. doi:10.1016/j.scitotenv.2011.11.006
505. Skubała, P., & Kafel, A. (2004). Oribatid mite communities and metal bioaccumulation in oribatid species (Acari, Oribatida) along the heavy metal gradient in forest ecosystems. *Environmental Pollution*, *132*(1), 51–60. doi:10.1016/j.envpol.2004.03.025
506. Skubała, P., Rola, K., Osyczka, P., & Kafel, A. (2014). Oribatid mite communities on lichens in heavily contaminated post-smelting dumps. *Archives of Environmental Contamination and Toxicology*, *67*(4), 578–592. doi:10.1007/s00244-014-0066-y
507. Smit, C. E., Schouten, A. J., Van Den Brink, P. J., van Esbroek, M. L. P., & Posthuma, L. (2002). Effects of zinc contamination on a natural nematode community in outdoor soil

mesocosms. *Archives of Environmental Contamination and Toxicology*, *42*(2), 205–216. doi:10.1007/s00244-001-0029-y
508. Smith, R., Mcswiney, C., Grandy, A., Suwanwaree, P., Snider, R., & Robertson, G. (2008). Diversity and abundance of earthworms across an agricultural land-use intensity gradient. *Soil & Tillage Research*, *100*(1–2), 83–88. doi:10.1016/j.still.2008.04.009
509. Smith, J., Potts, S., & Eggleton, P. (2008). The value of sown grass margins for enhancing soil macrofaunal biodiversity in arable systems. *Agriculture, Ecosystems & Environment*, *127*(1–2), 119–125. doi:10.1016/j.agee.2008.03.008
510. Solomou, A. D., Sfougaris, A. I., Vavoulidou, E. M., & Csuzdi, C. (2013). Species richness and density of earthworms in relation to soil factors in Olive orchard production systems in central Greece. *Communications in Soil Science and Plant Analysis*, *44*(1–4), 301–311. doi:10.1080/00103624.2013.741904
511. Solomou, A. D., Sfougaris, A. I., Vavoulidou, E. M., & Csuzdi, C. (2012) The effects of farming practices on earthworm dynamics in olive groves of central Greece. *Zoology in the Middle East*, *58*, 119-126. doi:10.1080/09397140.2012.10648993
512. Song, Z.-W., Zhang, B., Tian, Y.-L., Deng, A.-X., Zheng, C.-Y., Islam, M. N., … Zhang, W.-J. (2014). Impacts of nighttime warming on the soil nematode community in a winter wheat field of Yangtze delta plain, China. *Journal of Integrative Agriculture*, *13*(7), 1477–1485. doi:10.1016/s2095-3119(14)60807-8
513. Song, M., Li, X., Jing, S., Lei, L., Wang, J., & Wan, S. (2016). Responses of soil nematodes to water and nitrogen additions in an old-field grassland. *Applied Soil Ecology: A Section of Agriculture, Ecosystems & Environment*, *102*, 53–60. doi:10.1016/j.apsoil.2016.02.011
514. Song, L., Liu, J., Yan, X., Chang, L., & Wu, D. (2016). Euedaphic and hemiedaphic Collembola suffer larger damages than epedaphic species to nitrogen input. *Environmental Pollution*, *208*(Pt B), 413–415. doi:10.1016/j.envpol.2015.10.008
515. Soong, J. L., Dam, M., Wall, D. H., & Cotrufo, M. F. (2017). Below-ground biological responses to pyrogenic organic matter and litter inputs in grasslands. *Functional Ecology*, *31*(1), 260–269. doi:10.1111/1365-2435.12693
516. Spurgeon, D. J., & Hopkin, S. P. (1999). Seasonal variation in the abundance, biomass and biodiversity of earthworms in soils contaminated with metal emissions from a primary smelting works. *The Journal of Applied Ecology*, *36*(1), 173–183. doi:10.1046/j.1365-2664.1999.00389.x
517. Spurgeon, D. J., & Hopkin, S. P., (1999). Seasonal variation in the abundance, biomass and biodiversity of earthworms in soils contaminated with metal emissions from a primary smelting works. *Journal of Applied Ecology 36*(1), 173-183. doi: 10.1046/j.1365-2664.1999.00389.x
518. St. John, M. G., Bellingham, P. J., Walker, L. R., Orwin, K. H., Bonner, K. I., Dickie, I. A., … Wardle, D. A. (2012). Loss of a dominant nitrogen-fixing shrub in primary succession: consequences for plant and below-ground communities. *The Journal of Ecology*, *100*(5), 1074–1084. doi:10.1111/j.1365-2745.2012.02000.x
519. St. John, M.G., Bagatto, G., Behan-Pelletier, V., Lindquist, E. E., Shorthouse, J. D., & Smith, I. M. (2002). Mite (Acari) colonization of vegetated mine tailings near Sudbury, Ontario, Canada. *Plant and Soil 245*, 295–305. doi: 10.1023/A:1020453912401
520. Steinwandter, M., Schlick-Steiner, B. C., Seeber, G. U. H., Steiner, F. M., & Seeber, J. (2017). Effects of Alpine land-use changes: Soil macrofauna community revisited. *Ecology and Evolution*, *7*(14), 5389–5399. doi:10.1002/ece3.3043

521. Steinwandter, M., Rief, A., Scheu, S., Traugott, M., & Seeber, J. (2018). Structural and functional characteristics of high alpine soil macro-invertebrate communities. *European Journal of Soil Biology*, *86*, 72–80. doi:10.1016/j.ejsobi.2018.03.006

522. Sterzyńska, M., Tajovský, K., & Nicia, P. (2015). Contrasting responses of millipedes and terrestrial isopods to hydrologic regime changes in forested montane wetlands. *European Journal of Soil Biology*, *68*, 33–41. doi:10.1016/j.ejsobi.2015.03.005

523. Sterzyńska, M., Shrubovych, J., & Nicia, P. (2017). Impact of plant invasion (Solidago gigantea L.) on soil mesofauna in a riparian wet meadows. *Pedobiologia*, *64*, 1–7. doi:10.1016/j.pedobi.2017.07.004

524. Stevnbak, K., Maraldo, K., Georgieva, S., Bjørnlund, L., Beier, C., Schmidt, I. K., & Christensen, S. (2012). Suppression of soil decomposers and promotion of long-lived, root herbivorous nematodes by climate change. *European Journal of Soil Biology*, *52*, 1–7. doi:10.1016/j.ejsobi.2012.04.001

525. Sticht, C., Schrader, S., Giesemann, A., & Weigel, H.-J. (2009). Sensitivity of nematode feeding types in arable soil to free air CO2 enrichment (FACE) is crop specific. *Pedobiologia*, *52*(5), 337–349. doi:10.1016/j.pedobi.2008.12.001

526. Sticht, C., Schrader, S., Giesemann, A., & Weigel, H.-J. (2008). Atmospheric CO2 enrichment induces life strategy- and species-specific responses of collembolans in the rhizosphere of sugar beet and winter wheat. *Soil Biology & Biochemistry*, *40*(6), 1432–1445. doi:10.1016/j.soilbio.2007.12.022

527. Sticht, C., Schrader, S., Giesemann, A., & Weigel, H.-J. (2006). Effects of elevated atmospheric CO2 and N fertilization on abundance, diversity and C-isotopic signature of collembolan communities in arable soil. *Applied Soil Ecology: A Section of Agriculture, Ecosystems & Environment*, *34*(2–3), 219–229. doi:10.1016/j.apsoil.2006.01.007

528. Stinson, K. A., Frey, S. D., Jackson, M. R., Coates-Connor, E., Anthony, M., & Martinez, K. (2018). Responses of non-native earthworms to experimental eradication of garlic mustard and implications for native vegetation. *Ecosphere (Washington, D.C)*, *9*(7), e02353. doi:10.1002/ecs2.2353

529. Sun, F., Tariq, A., Chen, H., He, Q., Guan, Y., Pan, K., … Gu, Y. (2017). Effect of nitrogen and phosphorus application on agricultural soil food webs. *Archiv Fur Acker- Und Pflanzenbau Und Bodenkunde*, *63*(8), 1176–1186. doi:10.1080/03650340.2016.1266483

530. Sun, F., Pan, K., Li, Z., Wang, S., Tariq, A., Olatunji, O. A., … Wu, X. (2018). Soybean supplementation increases the resilience of microbial and nematode communities in soil to extreme rainfall in an agroforestry system. *The Science of the Total Environment*, *626*, 776–784. doi:10.1016/j.scitotenv.2018.01.063

531. Sun, X., Zhang, X., Zhang, S., Dai, G., Han, S., & Liang, W. (2013). Soil nematode responses to increases in nitrogen deposition and precipitation in a temperate forest. *PloS One*, *8*(12), e82468. doi:10.1371/journal.pone.0082468

532. Svendsen, T. S., Grønvold, J., Holter, P., & Sommer, C. (2003). Field effects of ivermectin and fenbendazole on earthworm populations and the disappearance of dung pats from bolus-treated cattle. *Applied Soil Ecology: A Section of Agriculture, Ecosystems & Environment*, *24*(3), 207–218. doi:10.1016/s0929-1393(03)00096-9

533. Tabaglio, V., Gavazzi, C., & Menta, C. (2009). Physico-chemical indicators and microarthropod communities as influenced by no-till, conventional tillage and nitrogen fertilisation after four years of continuous maize. *Soil & Tillage Research*, *105*(1), 135–142. doi:10.1016/j.still.2009.06.006

534. Takeda, M., Nakamoto, T., Miyazawa, K., Murayama, Y., & Okada, H., (2009). Phosphorus availability and soil biological activity in an Andosol under compost application and winter cover cropping. *Applied Soil Ecology, 42* (2), 86-95. doi:10.1016/j.apsoil.2009.02.003.

535. Tammeorg, P., Parviainen, T., Nuutinen, V., Simojoki, A., Vaara, E., & Helenius, J. (2014). Effects of biochar on earthworms in arable soil: avoidance test and field trial in boreal loamy sand. *Agriculture, Ecosystems & Environment*, *191*, 150–157. doi:10.1016/j.agee.2014.02.023

536. Tanner, R. A., Varia, S., Eschen, R., Wood, S., Murphy, S. T., & Gange, A. C. (2013). Impacts of an invasive non-native annual weed, Impatiens glandulifera, on above- and below-ground invertebrate communities in the United Kingdom. *PloS One*, *8*(6), e67271. doi:10.1371/journal.pone.0067271

537. Taylor, A. R., & Wolters, V. (2005). Responses of oribatid mite communities to summer drought: The influence of litter type and quality. *Soil Biology & Biochemistry*, *37*(11), 2117–2130. doi:10.1016/j.soilbio.2005.03.015

538. TerAvest, D., Carpenter-Boggs, L., Thierfelder, C., & Reganold, J. P. (2015). Crop production and soil water management in conservation agriculture, no-till, and conventional tillage systems in Malawi. *Agriculture, Ecosystems & Environment*, *212*, 285–296. doi:10.1016/j.agee.2015.07.011

539. Thakur, M. P., Berg, M. P., Eisenhauer, N., & van Langevelde, F. (2014). Disturbance–diversity relationships for soil fauna are explained by faunal community biomass in a salt marsh. *Soil Biology & Biochemistry*, *78*, 30–37. doi:10.1016/j.soilbio.2014.06.021

540. Thomason, J. E., Savin, M. C., Brye, K. R., & Gbur, E. E. (2017). Native earthworm population dominance after seven years of tillage, burning, and residue level management in a wheat-soybean, double-crop system. *Applied Soil Ecology: A Section of Agriculture, Ecosystems & Environment*, *120*, 211–218. doi:10.1016/j.apsoil.2017.08.014

541. Timmerman, A., Bos, D., Ouwehand, J., & de Goede, R. G. M. (2006). Long-term effects of fertilisation regime on earthworm abundance in a semi-natural grassland area. *Pedobiologia*, *50*(5), 427–432. doi:10.1016/j.pedobi.2006.08.005

542. Tom, N., Raman, A., Hodgkins, D. S., & Nicol, H. (2006). Populations of soil organisms under continuous set stocked and high intensity-short duration rotational grazing practices in the central tablelands of New South Wales (Australia). *New Zealand Journal of Agricultural Research*, *49*(3), 261–272. doi:10.1080/00288233.2006.9513717

543. Tomar, V., Zhang, X., Li, Q., Jiang, Y., & Liang, W. (2009). Distribution of soil nematode communities along a section of Shen-Ha Highway. *Helminthologia*, *46*(4), 241–246. doi:10.2478/s11687-009-0044-5

544. Tosza, E., Dumnicka, E., Niklińska, M., & Rożen, A. (2010). Enchytraeid and earthworm communities along a pollution gradient near Olkusz (southern Poland). *European Journal of Soil Biology*, *46*(3–4), 218–224. doi:10.1016/j.ejsobi.2010.04.001

545. Towns, D. R., Wardle, D. A., Mulder, C. P. H., Yeates, G. W., Fitzgerald, B. M., Richard Parrish, G., … Bonner, K. I. (2009). Predation of seabirds by invasive rats: multiple indirect consequences for invertebrate communities. *Oikos (Copenhagen, Denmark)*, *118*(3), 420–430. doi:10.1111/j.1600-0706.2008.17186.x

546. Treonis, A. M., Unangst, S. K., Kepler, R. M., Buyer, J. S., Cavigelli, M. A., Mirsky, S. B., & Maul, J. E. (2018). Characterization of soil nematode communities in three cropping systems through morphological and DNA metabarcoding approaches. *Scientific Reports*, *8*(1), 2004. doi:10.1038/s41598-018-20366-5

547. Treonis, A. M., Austin, E. E., Buyer, J. S., Maul, J. E., Spicer, L., & Zasada, I. A. (2010). Effects of organic amendment and tillage on soil microorganisms and microfauna. *Applied Soil Ecology: A Section of Agriculture, Ecosystems & Environment*, *46*(1), 103–110. doi:10.1016/j.apsoil.2010.06.017

548. Tsiafouli, M. A., Thébault, E., Sgardelis, S. P., de Ruiter, P. C., van der Putten, W. H., Birkhofer, K., … Hedlund, K. (2015). Intensive agriculture reduces soil biodiversity across Europe. *Global Change Biology*, *21*(2), 973–985. doi:10.1111/gcb.12752
549. Tsiafouli, M. A., Kallimanis, A. S., Katana, E., Stamou, G. P., & Sgardelis, S. P. (2005). Responses of soil microarthropods to experimental short-term manipulations of soil moisture. *Applied Soil Ecology: A Section of Agriculture, Ecosystems & Environment*, *29*(1), 17–26. doi:10.1016/j.apsoil.2004.10.002
550. Twardowski, J., Gruss, I., & Kordas, L. (2017). Effects of soil regeneration methods on beneficial mesofauna in a spring triticale field. *Journal of Central European Agriculture*, *18*(3), 616–631. doi:10.5513/jcea01/18.3.1935
551. Ulyshen, M. D., Klooster, W. S., Barrington, W. T., & Herms, D. A. (2011). Impacts of emerald ash borer-induced tree mortality on leaf litter arthropods and exotic earthworms. *Pedobiologia*, *54*(5–6), 261–265. doi:10.1016/j.pedobi.2011.05.001
552. Vacht, P., Puusepp, L., & Koff, T., (2018). The use of oribatid mites and diatoms as combined indicators of contaminations from multiple origins in riparian zone forest soils in Estonia*. Baltic Forestry, 24*(1), 24-35.
553. Van Aarde, R. J., Ferreira, S. M., & Wassenaar, T. D. (2004). Do feral house mice have an impact on invertebrate communities on sub-Antarctic Marion Island? *Austral Ecology*, *29*(2), 215–224. doi:10.1111/j.1442-9993.2004.01341.x
554. van Diepeningen, A. D., de Vos, O. J., Korthals, G. W., & van Bruggen, A. H. C. (2006). Effects of organic versus conventional management on chemical and biological parameters in agricultural soils. *Applied Soil Ecology: A Section of Agriculture, Ecosystems & Environment*, *31*(1–2), 120–135. doi:10.1016/j.apsoil.2005.03.003
555. van Gestel, C. A. M., Koolhaas, J. E., Hamers, T., van Hoppe, M., van Roovert, M., Korsman, C., & Reinecke, S. A. (2009). Effects of metal pollution on earthworm communities in a contaminated floodplain area: Linking biomarker, community and functional responses. *Environmental Pollution*, *157*(3), 895–903. doi:10.1016/j.envpol.2008.11.002
556. van Schaik, L., Palm, J., Klaus, J., Zehe, E., & Schröder, B. (2016). Potential effects of tillage and field borders on within-field spatial distribution patterns of earthworms. *Agriculture, Ecosystems & Environment*, *228*, 82–90. doi:10.1016/j.agee.2016.05.015
557. Vandecasteele, B., Samyn, J., Quataert, P., Muys, B., & Tack, F. M. G. (2004). Earthworm biomass as additional information for risk assessment of heavy metal biomagnification: a case study for dredged sediment-derived soils and polluted floodplain soils. *Environmental Pollution*, *129*(3), 363–375. doi:10.1016/j.envpol.2003.12.007
558. van Vliet, P. C. J., Coleman, D. C., & Hendrix, P. F. (1997). Population dynamics of Enchytraeidae (Oligochaeta) in different agricultural systems. *Biology and Fertility of Soils*, *25*(2), 123–129. doi:10.1007/s003740050291
559. Veen, G. F., Olff, H., Duyts, H., & van der Putten, W. H. (2010). Vertebrate herbivores influence soil nematodes by modifying plant communities. *Ecology*, *91*(3), 828–835. doi:10.1890/09-0134.1
560. Veen, G. F. C., De Long, J. R., Kardol, P., Sundqvist, M. K., Snoek, L. B., & Wardle, D. A. (2017). Coordinated responses of soil communities to elevation in three subarctic vegetation types. *Oikos (Copenhagen, Denmark)*, *126*(11), 1586–1599. doi:10.1111/oik.04158
561. Verble-Pearson, R. M., & Yanoviak, S. P. (2014). Effects of fire intensity on litter arthropod communities in Ozark oak forests, Arkansas, U.S.A. *The American Midland Naturalist*, *172*(1), 14–24. doi:10.1674/0003-0031-172.1.14

562. Verhoef, H. A., Verspagen, J. M. H., & Zoomer, H. R. (2000). Direct and indirect effects of ultraviolet-B radiation on soil biota, decomposition and nutrient fluxes in dune grassland soil systems. *Biology and Fertility of Soils*, *31*(5), 366–371. doi:10.1007/s003749900181
563. Vestergård, M., Dyrnum, K., Michelsen, A., Damgaard, C., & Holmstrup, M. (2015). Long-term multifactorial climate change impacts on mesofaunal biomass and nitrogen content. *Applied Soil Ecology: A Section of Agriculture, Ecosystems & Environment*, *92*, 54–63. doi:10.1016/j.apsoil.2015.03.002
564. Villenave, C., Saj, S., Pablo, A.-L., Sall, S., Djigal, D., Chotte, J.-L., & Bonzi, M. (2010). Influence of long-term organic and mineral fertilization on soil nematofauna when growing Sorghum bicolor in Burkina Faso. *Biology and Fertility of Soils*, *46*(7), 659–670. doi:10.1007/s00374-010-0471-y
565. Vincent, Q., Leyval, C., Beguiristain, T., & Auclerc, A. (2018). Functional structure and composition of Collembola and soil macrofauna communities depend on abiotic parameters in derelict soils. *Applied Soil Ecology: A Section of Agriculture, Ecosystems & Environment*, *130*, 259–270. doi:10.1016/j.apsoil.2018.07.002
566. Vincent, Q., Auclerc, A., Beguiristain, T., & Leyval, C. (2018). Assessment of derelict soil quality: Abiotic, biotic and functional approaches. *The Science of the Total Environment*, *613–614*, 990–1002. doi:10.1016/j.scitotenv.2017.09.118
567. Virtanen, R., Salminen, J., & Strömmer, R. (2008). Soil and decomposer responses to grazing exclusion are weak in mountain snow-beds. *Acta Oecologica (Montrouge, France)*, *33*(2), 207–212. doi:10.1016/j.actao.2007.10.009
568. Virto, I., Imaz, M. J., Enrique, A., Hoogmoed, W., & Bescansa, P. (2007). Burning crop residues under no-till in semi-arid land, Northern Spain—effects on soil organic matter, aggregation, and earthworm populations. *Soil Research*, *45*(6), 414. doi:10.1071/sr07021
569. Visioli, G., Menta, C., Gardi, C., & Conti, F. D. (2013). Metal toxicity and biodiversity in serpentine soils: application of bioassay tests and microarthropod index. *Chemosphere*, *90*(3), 1267–1273. doi:10.1016/j.chemosphere.2012.09.081
570. Wagner, D., Eisenhauer, N., & Cesarz, S. (2015). Plant species richness does not attenuate responses of soil microbial and nematode communities to a flood event. *Soil Biology & Biochemistry*, *89*, 135–149. doi:10.1016/j.soilbio.2015.07.001
571. Wall-Freckman, D., & Huang, S. P. (1998). Response of the soil nematode community in a shortgrass steppe to long-term and short-term grazing. *Applied Soil Ecology: A Section of Agriculture, Ecosystems & Environment*, *9*(1–3), 39–44. doi:10.1016/s0929-1393(98)00051-1
572. Wang, K., Qiao, Y., Zhang, H., Yue, S., Li, H., Ji, X., & Crowley, D. (2018). Influence of cadmium-contaminated soil on earthworm communities in a subtropical area of China. *Applied Soil Ecology: A Section of Agriculture, Ecosystems & Environment*, *127*, 64–73. doi:10.1016/j.apsoil.2018.02.026
573. Wang, X., Nielsen, U. N., Yang, X., Zhang, L., Zhou, X., Du, G., … Xiao, S. (2018). Grazing induces direct and indirect shrub effects on soil nematode communities. *Soil Biology & Biochemistry*, *121*, 193–201. doi:10.1016/j.soilbio.2018.03.007
574. Wang, S., Tan, Y., Fan, H., Ruan, H., & Zheng, A. (2015). Responses of soil microarthropods to inorganic and organic fertilizers in a poplar plantation in a coastal area of eastern China. *Applied Soil Ecology: A Section of Agriculture, Ecosystems & Environment*, *89*, 69–75. doi:10.1016/j.apsoil.2015.01.004
575. Wang, S., Chen, H. Y. H., Tan, Y., Fan, H., & Ruan, H. (2016). Fertilizer regime impacts on abundance and diversity of soil fauna across a poplar plantation chronosequence in coastal Eastern China. *Scientific Reports*, *6*(1), 20816. doi:10.1038/srep20816

576. Wang, Y., Qiao, J., He, C., Wang, Z., Luo, W., & Sheng, L. (2015). Towards multi-level biomonitoring of nematodes to assess risk of nitrogen and phosphorus pollution in Jinchuan Wetland of Northeast China. *Ecotoxicology*, *24*(10), 2190–2199. doi:10.1007/s10646-015-1550-5

577. Ward, S. E., Orwin, K. H., Ostle, N. J., Briones, J. I., Thomson, B. C., Griffiths, R. I., … Bardget, R. D. (2015). Vegetation exerts a greater control on litter decomposition than climate warming in peatlands. *Ecology*, *96*(1), 113–123. doi:10.1890/14-0292.1

578. Wei, X., Cao, R., Wu, X., Eisenhauer, N., & Sun, S. (2018). Effect of water table decline on the abundances of soil mites, springtails, and nematodes in the Zoige peatland of eastern Tibetan Plateau. *Applied Soil Ecology: A Section of Agriculture, Ecosystems & Environment*, *129*, 77–83. doi:10.1016/j.apsoil.2018.05.006

579. Wei, C., Zheng, H., Li, Q., Lü, X., Yu, Q., Zhang, H., … Han, X. (2012). Nitrogen addition regulates soil nematode community composition through ammonium suppression. *PloS One*, *7*(8), e43384. doi:10.1371/journal.pone.0043384

580. Whitford, W. G., & Steinberger, Y. (2012). Effects of seasonal grazing, drought, fire, and carbon enrichment on soil microarthropods in a desert grassland. *Journal of Arid Environments*, *83*, 10–14. doi:10.1016/j.jaridenv.2012.03.021

581. Wickings, K., & Grandy, A. S. (2013). Management intensity interacts with litter chemistry and climate to drive temporal patterns in arthropod communities during decomposition. *Pedobiologia*, *56*(2), 105–112. doi:10.1016/j.pedobi.2013.01.001

582. Wigginton, R. D., Pearson, J., & Whitcraft, C. R. (2014). Invasive plant ecosystem engineer facilitates community and trophic level alteration for brackish marsh invertebrates. *Ecosphere (Washington, D.C)*, *5*(4), art40. doi:10.1890/es13-00307.1

583. Wikars, L.-O., & Schimmel, J. (2001). Immediate effects of fire-severity on soil invertebrates in cut and uncut pine forests. *Forest Ecology and Management*, *141*(3), 189–200. doi:10.1016/s0378-1127(00)00328-5

584. Winkler, D., Bidló, A., Bolodár-Varga, B., Erdő, Á., & Horváth, A. (2018). Long-term ecological effects of the red mud disaster in Hungary: Regeneration of red mud flooded areas in a contaminated industrial region. *The Science of the Total Environment*, *644*, 1292–1303. doi:10.1016/j.scitotenv.2018.07.059

585. Winkler, D. (2014). Collembolan response to red mud pollution in Western Hungary. *Applied Soil Ecology: A Section of Agriculture, Ecosystems & Environment*, *83*, 219–229. doi:10.1016/j.apsoil.2013.07.006

586. Wu, P., Zhang, H., & Wang, Y. (2015). The response of soil macroinvertebrates to alpine meadow degradation in the Qinghai–Tibetan Plateau, China. *Applied Soil Ecology: A Section of Agriculture, Ecosystems & Environment*, *90*, 60–67. doi:10.1016/j.apsoil.2015.02.006

587. Wu, P., Zhang, H., Cui, L., Wickings, K., Fu, S., & Wang, C. (2017). Impacts of alpine wetland degradation on the composition, diversity and trophic structure of soil nematodes on the Qinghai-Tibetan Plateau. *Scientific Reports*, *7*(1). doi:10.1038/s41598-017-00805-5

588. Wu, T., Su, F., Han, H., Du, Y., Yu, C., & Wan, S. (2014). Responses of soil microarthropods to warming and increased precipitation in a semiarid temperate steppe. *Applied Soil Ecology: A Section of Agriculture, Ecosystems & Environment*, *84*, 200–207. doi:10.1016/j.apsoil.2014.07.003

589. Xiao, H. F., Tian, Y. H., Zhou, H. P., Ai, X. S., Yang, X. D., & Schaefer, D. A. (2014). Intensive rubber cultivation degrades soil nematode communities in Xishuangbanna, southwest China. *Soil Biology & Biochemistry*, *76*, 161–169. doi:10.1016/j.soilbio.2014.05.012

590. Xiong, Y., Shao, Y., Xia, H., Li, Z., & Fu, S. (2008). Selection of selective biocides on soil microarthropods. *Soil Biology & Biochemistry*, *40*(10), 2706–2709. doi:10.1016/j.soilbio.2008.07.014

591. Xu, J., Lie, G., & Xue, L. (2016). Effects of gap size on diversity of soil fauna in a Cunninghamia lanceolata stand damaged by an ice storm in southern China. *Journal of Forestry Research*, *27*(6), 1427–1434. doi:10.1007/s11676-016-0241-2
592. Xu, G.-L., Schleppi, P., Li, M.-H., & Fu, S.-L. (2009). Negative responses of Collembola in a forest soil (Alptal, Switzerland) under experimentally increased N deposition. *Environmental Pollution*, *157*(7), 2030–2036. doi:10.1016/j.envpol.2009.02.026
593. Xu, G.-L., Mo, J.-M., Zhou, G.-Y., & Fu, S.-L. (2006). Preliminary response of soil fauna to simulated N deposition in three typical subtropical forests. *Pedosphere*, *16*(5), 596–601. doi:10.1016/s1002-0160(06)60093-3
594. Xu, G.-L., Mo, J.-M., Fu, S.-L., Gundersen, P., Zhou, G.-Y., & Xue, J.-H. (2007). Response of soil fauna to simulated nitrogen deposition: a nursery experiment in subtropical China. *Journal of Environmental Sciences (China)*, *19*(5), 603–609. doi:10.1016/s1001-0742(07)60100-4
595. Xu, G.-L., Kuster, T. M., Günthardt-Goerg, M. S., Dobbertin, M., & Li, M.-H. (2012). Seasonal exposure to drought and air warming affects soil Collembola and mites. *PloS One*, *7*(8), e43102. doi:10.1371/journal.pone.0043102
596. Xu, G.-L., Fu, S.-L., Schleppi, P., & Li, M.-H. (2013). Responses of soil Collembola to long-term atmospheric CO2 enrichment in a mature temperate forest. *Environmental Pollution*, *173*, 23–28. doi:10.1016/j.envpol.2012.10.007
597. Yan, X., Wang, K., Song, L., Wang, X., & Wu, D. (2017). Daytime warming has stronger negative effects on soil nematodes than night-time warming. *Scientific Reports*, *7*, 44888. doi:10.1038/srep44888
598. Yan, X., Ni, Z., Chang, L., Wang, K., & Wu, D. (2015). Soil warming elevates the abundance of Collembola in the songnen plain of China. *Sustainability*, *7*(2), 1161–1171. doi:10.3390/su7021161
599. Yan, D., Yan, D., Song, X., Yu, Z., Peng, D., Ting, X., & Weng, B. (2018). Community structure of soil nematodes under different drought conditions. *Geoderma*, *325*, 110–116. doi:10.1016/j.geoderma.2018.03.028
600. Yang, Z., Nolte, S., & Wu, J. (2017). Tidal flooding diminishes the effects of livestock grazing on soil micro-food webs in a coastal saltmarsh. *Agriculture, Ecosystems & Environment*, *236*, 177–186. doi:10.1016/j.agee.2016.12.006
601. Yang, Y.-R., Li, X.-G., Zhou, Z.-G., Zhang, T.-L., & Wang, X.-X. (2016). Differential responses of soil nematode community to pig manure application levels in Ferric Acrisols. *Scientific Reports*, *6*, 35334. doi:10.1038/srep35334
602. Yang, X., Warren, M., & Zou, X. (2007). Fertilization responses of soil litter fauna and litter quantity, quality, and turnover in low and high elevation forests of Puerto Rico. *Applied Soil Ecology: A Section of Agriculture, Ecosystems & Environment*, *37*(1–2), 63–71. doi:10.1016/j.apsoil.2007.03.012
603. Yong-zhong, S., Xue-fen, W., Rong, Y., Xiao, Y., & Wen-jie, L. (2012). Soil fertility, salinity and nematode diversity influenced by Tamarix ramosissima in different habitats in an arid desert oasis. *Environmental Management*, *50*(2), 226–236. doi:10.1007/s00267-012-9872-z
604. Yardirn, E. N., & Edwards, C. A. (1998). The effects of chemical pest, disease and weed management practices on the trophic structure of nematode populations in tomato agroecosystems. *Applied Soil Ecology: A Section of Agriculture, Ecosystems & Environment*, *7*(2), 137–147. doi:10.1016/s0929-1393(97)00036-x
605. Yin, X., Ma, C., He, H., Wang, Z., Li, X., Fu, G., … Zheng, Y. (2018). Distribution and diversity patterns of soil fauna in different salinization habitats of Songnen Grasslands, China. *Applied Soil Ecology: A Section of Agriculture, Ecosystems & Environment*, *123*, 375–383. doi:10.1016/j.apsoil.2017.09.034

606. Zaller, J. G., Cantelmo, C., Santos, G. D., Muther, S., Gruber, E., Pallua, P., … Faber, F. (2018). Herbicides in vineyards reduce grapevine root mycorrhization and alter soil microorganisms and the nutrient composition in grapevine roots, leaves, xylem sap and grape juice. *Environmental Science and Pollution Research International*, *25*(23), 23215–23226. doi:10.1007/s11356-018-2422-3

607. Zaller, J. G., Caldwell, M. M., Flint, S. D., Ballaré, C. L., Scopel, A. L., & Sala, O. E. (2009). Solar UVB and warming affect decomposition and earthworms in a fen ecosystem in Tierra del Fuego, Argentina. *Global Change Biology*, *15*(10), 2493–2502. doi:10.1111/j.1365-2486.2009.01970.x

608. Zaller, J., & Arnone, J. (2011). Earthworm responses to plant species' loss and elevated CO2 in calcareous grassland. *Plant and Soil 208*, 1–8. https://doi.org/10.1023/A:1004424720523

609. Zhan, L., Li, S., Xu, Y., Zhang, X., Pei, X., Pan, F., … Chen, P. (2014). Soil fauna community in the black soil of Northeast China under different tillage systems. *Acta Agriculturae Scandinavica. Section B, Soil and Plant Science*, *64*(5), 462–469. doi:10.1080/09064710.2014.920906

610. Zhang, Z., Zhang, X., Mahamood, M., Zhang, S., Huang, S., & Liang, W. (2016). Effect of long-term combined application of organic and inorganic fertilizers on soil nematode communities within aggregates. *Scientific Reports*, *6*(1). doi:10.1038/srep31118

611. Zhang, Z.-Y., Zhang, X.-K., Jhao, J.-S., Zhang, X.-P., & Liang, W.-J. (2015). Tillage and rotation effects on community composition and metabolic footprints of soil nematodes in a black soil. *European Journal of Soil Biology*, *66*, 40–48. doi:10.1016/j.ejsobi.2014.11.006

612. Zhang, X., Li, Q., Zhu, A., Liang, W., Zhang, J., & Steinberger, Y. (2012). Effects of tillage and residue management on soil nematode communities in North China. *Ecological Indicators*, *13*(1), 75–81. doi:10.1016/j.ecolind.2011.05.009

613. Zhang, X. K., Li, Q., Wang, S. B., Jiang, Y., & Liang, W. (2006). Effect of zinc addition to soil on nematode community structure. *Bulletin of Environmental Contamination and Toxicology*, *76*(4), 589–594. doi:10.1007/s00128-006-0960-8

614. Zhang, X., Ferris, H., Mitchell, J., & Liang, W. (2017). Ecosystem services of the soil food web after long-term application of agricultural management practices. *Soil Biology & Biochemistry*, *111*, 36–43. doi:10.1016/j.soilbio.2017.03.017

615. Zhang, W., Wang, X., Li, Q., Jiang, Y., & Liang, W. (2007). Soil nematode responses to heavy metal stress. *Helminthologia*, *44*(2), 87–91. doi:10.2478/s11687-007-0009-5

616. Zhang, S., Li, Q., Lü, Y., Zhang, X., & Liang, W. (2013). Contributions of soil biota to C sequestration varied with aggregate fractions under different tillage systems. *Soil Biology & Biochemistry*, *62*, 147–156. doi:10.1016/j.soilbio.2013.03.023

617. Zhang, S., Li, Q., Lü, Y., Sun, X., Jia, S., Zhang, X., & Liang, W. (2015). Conservation tillage positively influences the microflora and microfauna in the black soil of Northeast China. *Soil & Tillage Research*, *149*, 46–52. doi:10.1016/j.still.2015.01.001

618. Zhang, F., Gao, C., Wang, J., Lu, Y., Shen, Z., Liu, T., … Shen, Q. (2017). Coupling sugarcane yield to soil nematodes: Implications from different fertilization regimes and growth stages. *Agriculture, Ecosystems & Environment*, *247*, 157–165. doi:10.1016/j.agee.2017.06.020

619. Zhao, J., Wang, X., Shao, Y., Xu, G., & Fu, S. (2011). Effects of vegetation removal on soil properties and decomposer organisms. *Soil Biology & Biochemistry*, *43*(5), 954–960. doi:10.1016/j.soilbio.2011.01.010

620. Zhao, J., Wang, F., Li, J., Zou, B., Wang, X., Li, Z., & Fu, S. (2014). Effects of experimental nitrogen and/or phosphorus additions on soil nematode communities in a secondary tropical forest. *Soil Biology & Biochemistry*, *75*, 1–10. doi:10.1016/j.soilbio.2014.03.019

621. Zhao, H.-L., Li, J., Liu, R.-T., Zhou, R.-L., Qu, H., & Pan, C.-C. (2014). Effects of desertification on temporal and spatial distribution of soil macro-arthropods in Horqin sandy grassland, Inner Mongolia. *Geoderma*, *223–225*, 62–67. doi:10.1016/j.geoderma.2014.01.026
622. Zhao, C., Fu, S., Mathew, R. P., Lawrence, K. S., & Feng, Y. (2015). Soil microbial community structure and activity in a 100-year-old fertilization and crop rotation experiment. *Journal of Plant Ecology*, rtv007. doi:10.1093/jpe/rtv007
623. Zhong, S., Zeng, H., & Jin, Z. (2015). Responses of soil nematode abundance and diversity to long-term crop rotations in tropical China. *Pedosphere*, *25*(6), 844–852. doi:10.1016/s1002-0160(15)30065-5
624. Zhu, X., & Zhu, B. (2015). Diversity and abundance of soil fauna as influenced by long-term fertilization in cropland of purple soil, China. *Soil & Tillage Research*, *146*, 39–46. doi:10.1016/j.still.2014.07.004
625. Zolda, P. (2006). Nematode communities of grazed and ungrazed semi-natural steppe grasslands in Eastern Austria. *Pedobiologia*, *50*(1), 11–22. doi:10.1016/j.pedobi.2005.08.002
626. Zou, X. (1993). Species effects on earthworm density in tropical tree plantations in Hawaii. *Biology and Fertility of Soils*, *15*(1), 35–38. doi:10.1007/bf00336285

Data S2: Data Extraction Protocol:

Overview of steps in the meta-analysis

- Review the titles and abstracts of all papers gathered from the literature review
    - This will be done alongside Abstraktr
- For the papers that seem suitable from the titles and abstracts, the full text will be reviewed
- Papers that are suitable based on the full text will have data extracted

Title and abstract screening - General Overview

In order to be suitable for the meta-analysis the abstract should mention data that looks at the effect of a global change driver on soil invertebrates diversity.

A global change (GC) should belong to one (or more) of the following six categories:

- Land use intensification
- Habitat loss or fragmentation
- Pollution
- Nutrient enrichment
- Climate change
- Invasive species

A soil invertebrate includes many things, but not microbes.
Microbes are:

- Bacteria
- Archaea
- Fungi

We are also not including protozoa

Soil invertebrates (in this stage – the full text screening stage has a different protocol) *can* include (but is not limited to):

- Ants
- Termites
- Beetles
- Spiders
- Earthworms
- Larvae (of many invertebrates)
- Collembola
- Nematodes

- Mites
- Isopoda
- Diplopoda
- Chilopoda
- Molluscs (Gastropods)

The diversity data needs to include the diversity of all animals within the group(s), i.e., not just one or a couple of species.
The data will need to include diversity data, such as:
- Species richness
- Abundance
- Biomass
- Shannon diversity

Global Changes

We are expecting papers that contain a control (i.e., less intensive) sites compared to one (or more) more intensive sites. At each site they would have measured the diversity of the soil invertebrates.

Land Use Intensification

As we don't want too much overlap between the global change categories, it is likely that 'land use intensification' will only be applied to sites that have an increase in *mechanical* inputs. For example, a change in tillage, removal of yield (i.e., grass, wood), grazing. Changes in fertilizing rates, pesticide use will be in other GCD categories. A change from an organic system to an inorganic system will also be considered a land use intensification if the exact changes is not given (or can't be split apart).
A study may have data from different systems (i.e., a forest, a grazed grassland, an urban site), but cases must be within at least one/each system there is a change in intensification. We will only use the systems that have a change in intensification.

A note on Land Use Change

We will not be using any land use change studies in this meta-analysis. But will still be identifying them at this stage, so we could at a later date.
A land use change study is where multiple sites only exist in multiple types of systems. For example, a grazed grassland versus a forest versus an urban site. There is no change in intensification measured within any of the systems.

Habitat loss or fragmentation

The studies will have sites in habitats (of any system) that change in their size, isolation or disturbance.
They will (most likely) fall into three main categories:

- Changes in habitat amount across sites
- Changes in distance to the edge of the habitat across sites
- Changes in the connectivity of the habitat to other habitats

Pollution

Pollution studies will have multiple sites that vary in the amount (or type) of pollution.
Broadly, pollutions may be:

- Pesticides (targeted or non-targeted)
- PAHs
- Plastics
- Antibiotics
- Metals (Cd, Pb, Zn etc.)
- Asbestos
- radiation

Nutrient enrichment

As with pollution, nutrient enrichment studies will have sites that vary within or between two main types of nutrient enrichment - organic and inorganic.
Organic:

- Manure
- Mulch
- Sludge
- Slurry

Synthetic (i.e. chemicals including specific elements):

- N, P, K (and any mixture of them)
- Ca
- S

Climate change

Climate change studies will cover a whole range of treatments. Sites will often have a change in the intensity, frequency or amount of the treatment:
Treatments includes:

- Gas (N_2O, CO_2, CH_4)
- Fire (only in relation to climate change, not natural burning)
- Precipitation

- Temperature
- Mixture of climate treatment
- Extreme events

Irrigation is not a climate change, as this is assumes that crops get the water that is the normal amount. The treatment needs to be a manipulation away from the normal amount/frequency.

Invasive species

We foresee three main types of invasive species studies:
- Those where plants are the invasive species
- Those where animals are invasive (the animals may be aboveground or belowground
- Where pathogens are invasive

Studies will either be testing where sites have no invasive species versus where they do, and sites that have less invasive species than other sites.

Full Text Screening – General Overview

In order to be suitable the main text needs to present data (either in the text, table or figure), show appropriate methodology, and an appropriate global change driver.

1. Methods

The appropriate methods are as following:
- Soil core (any size)
- Soil\Leaf litter quadrats
- Soil pit
- Chemical expulsion (hot mustard, mustard, formalin, AITC)
- Berlese–Tullgren funnel sampling (technically done using a soil core, but may only mention this part)
- Pitfall traps only in combination with one of the above methods

The data will not be considered ‘soil biodiversity’ if *only* pitfall traps are used.

Mesocosms:
If the mesocosms have been taken from the field, and the community not manipulated or the soil sterilised, then it is fine to be included. If the experimental design had a fully manipulated communities (i.e., “we added four species”), and therefore the soil was probably sterilized, then we do not want these studies included.

2. Available data

Look through the paper for figures, tables, supplementary material, and data in the text.

We need means/averages from the different treatments, and standard deviation (SD) or standard error (SE(M)) or confidence intervals (CIs), and the sample size in the different treatments. For metrics related to species composition (Shannon, Simpsons, Evenness) we do not require the variances, so do not exclude papers that only include these.

We do not want PCA/MCA/CCA/multivariate analyses.

3. Global changes

It is fine for a study to have more than one global change driver.

Temporal versus spatial contrasts
We will accept studies that look at sites that vary in space as well as samples that vary in time (i.e., a site that is sampled when not polluted and again later when it has been polluted.)

Restoration (after pollution)
In an study that's investigating the impact of active restoration (or a study comparing different types of restorations), such as planting trees, this should not be included, as it's a land use change study. This include those with are occurring after mining activities (i.e. pollution studies). In area where there was mining (as an example of a pollution) and there is active restoration (i.e., planting trees), this study will be considered as a pollution study and a land use change study.

Compaction studies
Only included if in relation to another global change as the underlying cause, e.g., compaction due to grazing animals versus non, or other agricultural practices (in this case, it would be an intensification).
If they only look at the effect of compaction on diversity, then this is not suitable.

Climate studies
Most likely this will be experiments where e.g., rainfall or temperature is manipulated. Spatial sites with different in temperature or precipitation will probably be excluded, unless, the authors specifically state they are trying to reproduce climate change with a gradient.
However, some studies may be temporal sampling (over many years) to try and understand changes in climate, these are suitable.

Fire
Most of the time, fire studies are prescribed burning. In which case they are an intensification study. However, if the paper is framed in the context of climate change, then it would be included in climate change.

Altitudinal studies
Similar to climate studies, if the author is using elevation to reproduce a climate change scenario, we will include them. If it is just to look at the effect of elevation, exclude.

Wetlands: include them (including paddy fields too)
As long as they have sampled the soil under the water, these can be included.

GMOs
Exclude if only investigating the effect of the GMO crop. But can be included if, for example, they include land use intensification impacts in a GMO-crop field and non-GMO-crop field.

Sludge application
Unless they quantify the chemicals within the sludge, it is only nutrient enrichment. Otherwise it can also be a pollution study.

Vermicomposting
If vermicompost had been applied to the soil, it is nutrient enrichment. If they are just looking at the process of vermicomposting, then exclude.

Acidification
Changes in pH are not considered as a global change in this meta-analysis. Acidification can be nutrient enrichment or pollution depending on the study design and focus:
- studies that report addition of N or S (e.g. H2SO4) will be nutrient enrichment (S is a nutrient)
- Studies that focus on the increased solubility in toxic heavy metals (Al and Fe) resulting from acidification will be tagged pollution

Data Extraction Protocol – General Overview

1) Check the paper and look at the figures and tables: if the paper does not report standard deviations or errors for abundance and richness data, the paper can be readily excluded (but not for Shannon or other diversity metrics).

2) If the paper is relevant, start data collection: Create an excel sheet in the Screening/Studies folder, named with the ID of the paper (also, name the folder the same).

3) Identify the different cases by reading methods and figures (different drivers, metrics of diversity and so on), and report them in the excel sheet.

4) Collect the data: using WebplotDigitizer for the figures (WebPlotDigitizer), for the tables you can try using pdftoexcel (that does not always work though: https://www.pdftoexcel.com/), All the data collected that way need to be pasted to the excel sheet associated with the paper in the Studies folder, in a folder named with the ID (this folder should also contain the PDF of the study and anything else needed during data extraction). If the study has multiple drivers, collect all the cases.

5) Fill the data extraction table with the data collected.

Data Extraction Protocol – General Information

Means and SDs

If a study does not have SDs (or SEs or CIs) for species richness, abundance or biomass values, then we are excluding the data.

For Shannons and evenness values do not exclude data if SDs etc. are missing.

If a case gives an error bar, but does not say what it is, then mark it as ‘Unknown’.

Definition of Case

Within each paper, there can be any number of cases. These can corresponded to :

- Different taxonomic groups
- Different biodiversity measurements (e.g. shannon vs. taxa richness)
- Different types of global change (e.g. CO_2 and temperature treatments, each with a control)
- Different land use covers/habitat cover/plant species richness/cover crops
- Separated lab and field experiments
- Data from geographically separated areas (different countries, different landscapes if the primary studies also have them separated)

How to deal with multiple treatment levels

When paper reports several global change intensities (like a gradient) we only keep the highest intensities (i.e., the control and the most extreme treatment).

If a paper has a replicated design (i.e., a control for every treatment plot), then multiple comparisons can be made (this design is unlikely to appear in the literature often). The comparisons made depend on the number of different “treatments”

How to deal with multiple cases within and across global change types

Take the following experiment design, where an author manipulated temperature and CO_2. There would be two cases in this; *a* - where CO_2 was manipulated and the other “treatment” is at its control, and *b* - where temperature is increased and the other “treatment” is at the control.

	Ambient temperature	Increased temperature

Ambient CO2	a, b	b
Increased CO2	a	

So the rule is: when there are more than one treatment, each treatment should be a case, where all other treatments are at their lowest intensity. This also applies when the treatments are in different global change categories. It also applies if there are more than two GCs.

How to deal with multiple sampling times

When paper only reports several sampling dates: Only keep the last point in time for both the control and treatment. This includes recovery studies where the treatment stopped but authors followed soil fauna recovery over time.

Relative abundance data

We only extract the total abundance, not the relative abundance.

Taxonomic level

We extract biodiversity data at the highest level of taxonomic resolution possible but above family level data (i.e., we don’t want abundances of a single species), enchytraeids are the exception to this rule.

If the paper reports the response of ALL the taxonomic groups they considered (for example they considered macrofauna, and reported the means and variance at this level) but they also report the response of *some but not all* of the taxonomic groups belonging to the macrofauna category they found (earthworms, millipedes, etc.), then we would extract only the lower resolution (for example paper looking at macrofauna response, reporting the overall abundance of macrofauna, and the abundance of earthworms only, we extract only the macrofauna data).

If the data is split into, e.g., “nematodes, collembola and other mesofauna”?: If there is a total mean abundance, then just use the mean total abundance. If there is no mean total abundance, then discard the “others” group, and just use, in this case, nematodes and collembola.

We don’t extract at the lowest functional group reported. For example, if the author reports for different feeding groups of nematodes, this should be pooled. Unless there is strong reason to believe that not all nematodes were reported.

Functional/feeding groups

Pool across different functional/feeding/r-k-strategies/taxonomic groups, if all of them are reported. If only some are reported, they will need to be excluded (for example, a study might study all earthworm ecological groups, but only present the epigeics).

To pool:
Take the average of the mean values of different functional groups for control and treatment levels.
If errors are reported in SE you have to transform to SD first.
SD = SE * SQRT(n)
Where *n* is the number of samples that calculated that SE.

Then use the formula below to calculate the pooled standard deviation for each of the control and the treatment levels

$$s_{\text{pooled}} = \sqrt{\frac{s_1^{\,2} + s_2^{\,2} + \ldots + s_k^{\,2}}{k}}$$

Where s1, s2, … are the standard deviation associated to each functional group,
And k is the number of functional groups

In excel: SQRT(SUM(s1^2, s2^2, S3^2….)/[the number of S's])

Depth profile

When authors report diversities for different depth profiles (i.e., 0-10cm, 10-20cm), then only use the data for the top/shallowest depth. But always use the aggregated data if it is given.

Soils with different land covers/uses?

We only focus on comparisons within similar land cover/uses (forests/grasslands/croplands). But there can of course be cases across different land covers etc.

Decimal values

Please use American/English conventions for inputting decimal points (i.e., a fullstop [.] not a comma).

Coordinates

Use d/m/s format in the sheet.

Pitfall trap results

We don't collect those data, except if the authors report combined pitfall+soil samplings

Duplicated data and studies

When the paper reports different times only use the last point in time.

However, when reported in separate studies (e.g. one study published in 2013 reports data for 2013, and another paper about the same experiment/site reports data in 2015), extract both papers.

Boxplots

Only consider boxplots if it states it's the mean.

Unclear control sites

If different treatments are compared and it’s not clear which is the least intense (i.e. the control) or the authors don’t state, the paper is excluded (for example if there are several crop rotation treatments and none of them can be clearly identified as the least intense compared to monoculture treatment, this would be excluded). A control is needed.

Confidence interval

Use the value of the upper limit of the confidence interval in the “_SD” columns.

Plant diversity experiments

If a paper has data across a manipulated plant diversity gradient, then only use all the data, but have the different diversity groups as different cases.

Data Extraction Protocol – datasheet information

Information on the data collected in the data sheets.

**Column_Name**	**Description**
ID	Unique ID (number) given to each paper produced from the literature search
Author	The surname of the first author on the paper
Title	The title of the paper
year	The year the paper was published
DOI	The DOI of the paper
Case_ID	A unique identifier (character) for each case within the paper. A case is a comparison between a control and treatment. Different taxa groups, different measurements, different habitat types will each be different cases
driver	Which broad Global Change category is being measured
UniqueID	A unique identifier based on the paper ID, driver, and case ID

GCDType	Within the broad global change category, which stressor is being studied
System	What system is the study performed in
Harmonised	The taxa group being studied. The names have been harmonised across all papers (correct spelling errors, capitalisations etc)
GSBA	The taxa group being studied based on the categories presented in the Global Soil Biodiversity Atlas
Body.Size	The size of the group being studied. Either given in the paper or determined based on the GSBA classification
Control_mean	The mean of the biodiversity in the control sites
Control_SD	The standard deviation of the mean in the control sites. This has been harmonised from all variances given in papers.
Control_N	The number of samples that were used to calculate the mean in the control sites
Treatment_mean	The mean of the biodiversity in the treatment sites
Treatment_SD	The standard deviation of the mean in the treatment sites. This has been harmonised from all variances given in papers.
Treatment_N	The number of samples that were used to calculate the mean in the treatment sites
Measurement	What type of biodiversity measurement is being measured in this case
Error	What type of error (for the means) is being measured
yi	The effect size of the case (Hedges)
vi	The variance of the effect size for the case
effect	The effect size for the case (Hedges) - this is the same as yi, just a different column name
var	The variance of the effect size of the case - this is the same as vi, just a different column name
sei	The standard error of the effect size of the case (i.e., the square root of the sampling variance of the effect size)
year.c	The year the paper was published but centred on the mean
Data_Source	Where the data for this case was located in the original publication
